# The architecture of an empirical genotype‐phenotype map

**DOI:** 10.1111/evo.13487

**Published:** 2018-05-25

**Authors:** José Aguilar‐Rodríguez, Leto Peel, Massimo Stella, Andreas Wagner, Joshua L. Payne

**Affiliations:** ^1^ Department of Evolutionary Biology and Environmental Studies University of Zurich Zurich Switzerland; ^2^ Swiss Institute of Bioinformatics Lausanne Switzerland; ^3^ Current Address: Department of Biology, Stanford University, Stanford CA, USA; Department of Chemical and Systems Biology, Stanford University Stanford CA USA; ^4^ Institute of Information and Communication Technologies, Electronics and Applied Mathematics Université Catholique de Louvain Louvain‐la‐Neuve Belgium; ^5^ Namur Center for Complex Systems University of Namur Namur Belgium; ^6^ Institute for Complex Systems Simulation, Department of Electronics and Computer Science University of Southampton Southampton United Kingdom; ^7^ The Santa Fe Institute Santa Fe New Mexico USA; ^8^ Institute for Integrative Biology, ETH Zurich Switzerland

**Keywords:** Transcription factors, molecular evolution, mutations, evolvability

## Abstract

Recent advances in high‐throughput technologies are bringing the study of empirical genotype‐phenotype (GP) maps to the fore. Here, we use data from protein‐binding microarrays to study an empirical GP map of transcription factor (TF) ‐binding preferences. In this map, each genotype is a DNA sequence. The phenotype of this DNA sequence is its ability to bind one or more TFs. We study this GP map using genotype networks, in which nodes represent genotypes with the same phenotype, and edges connect nodes if their genotypes differ by a single small mutation. We describe the structure and arrangement of genotype networks within the space of all possible binding sites for 525 TFs from three eukaryotic species encompassing three kingdoms of life (animal, plant, and fungi). We thus provide a high‐resolution depiction of the architecture of an empirical GP map. Among a number of findings, we show that these genotype networks are “small‐world” and assortative, and that they ubiquitously overlap and interface with one another. We also use polymorphism data from *Arabidopsis thaliana* to show how genotype network structure influences the evolution of TF‐binding sites *in vivo*. We discuss our findings in the context of regulatory evolution.

Evolution can be abstracted as an exploration of genotype space—the space of all possible genotypes (Maynard Smith 1970). This space is populated by intersecting sets of genotypes that each correspond to a distinct phenotype. The organization of genotype space into such genotype sets is described by the genotype‐phenotype (GP) map (Burns 1970; Alberch 1991), an object of central importance in developmental and evolutionary biology, with important implications for medicine (Pigliucci 2010; Wagner and Zhang 2011; Lehner 2013).

Most of what we know about GP maps comes from computational models of biological systems (Lipman and Wilbur 1991; Schuster et al. 1994; Ciliberti et al. 2007; Rodrigues and Wagner 2009; Cotterell and Sharpe 2010; Salazar‐Ciudad and Marín‐Riera 2013; Greenbury et al. 2014). These include models that map RNA sequence genotypes onto secondary structure phenotypes (Schuster et al. 1994; Aguirre et al. 2011), simplified amino acid sequence genotypes onto lattice‐based, structural phenotypes (Lipman and Wilbur 1991; Bornberg‐Bauer and Chan 1999), regulatory circuit genotypes onto gene expression phenotypes (Ciliberti et al. 2007), and metabolic genotypes onto nutrient utilization phenotypes (Rodrigues and Wagner 2009). GP maps have also been studied in nonbiological systems, including self‐replicating computer programs (Fortuna et al. 2017), evolutionary algorithms (Hu et al. 2012), and field programmable gate arrays (Raman and Wagner 2011). Despite all that differentiates these systems, their GP maps have much in common (Ahnert 2017). First, they are many‐to‐one, meaning that multiple genotypes have the same phenotype. Second, the distribution of genotypes per phenotype is heavily skewed, such that most phenotypes are realized by few genotypes, and a few phenotypes are realized by many genotypes. Third, genotypes with the same phenotype tend to be mutationally interconnected, meaning that it is possible to transform any one of these genotypes into any other via a series of small mutations that preserve the phenotype. Such sets of mutationally interconnected genotypes are known as genotype networks (aka neutral networks (Schuster et al. 1994)). A fourth commonality is that the genotype networks of different phenotypes tend to overlap and interface with one another (Wagner 2008; Barve and Wagner 2013; Payne and Wagner 2013; Wagner 2014). We refer to the comprehensive description of the structure and arrangement of genotype networks within genotype space as the architecture of a GP map (Ferrada 2014).

The architecture of a GP map has important implications for evolution, influencing the rate of adaptation (Draghi et al. 2010; Manrubia and Cuesta 2015), the “findability” of genotypes and phenotypes in evolutionary searches (Cowperthwaite et al. 2008; McCandlish 2013; Schaper and Louis 2014), as well as their robustness and evolvability (Wagner 2008). It is therefore important to move beyond the study of GP maps derived from computational models, and to begin to study the architecture of GP maps that are derived from experimental data.

We currently know very little about the architecture of such empirical GP maps. The reason is that the genotype spaces of most biological systems are so large that it is not possible to experimentally assay a phenotype for all possible genotypes (Louis 2016). This is especially problematic when studying the architecture of a GP map, where it is necessary to assay a large number of phenotypes. Recent advances in high‐throughput sequencing and chip‐based technologies are beginning to mitigate this problem by providing localized descriptions of GP maps for macromolecules such as RNA and proteins (Fowler et al. 2010; Rowe et al. 2010; Hinkley et al. 2011; Jiménez et al. 2013; Melamed et al. 2013; Buenrostro et al. 2014; Findlay et al. 2014; Olson et al. 2014; Podgornaia and Laub 2015; Julien et al. 2016; Li et al. 2016; Puchta et al. 2016; Qiu et al. 2016; Sarkisyan et al. 2016). While insightful, these empirical GP maps still only describe a small subset of the genotype networks of a small number of phenotypes, and therefore cannot be used to characterize the architecture of a GP map.

In contrast, protein binding microarrays (Berger et al. 2006) provide comprehensive descriptions of transcription factor (TF) binding preferences to all possible, short DNA sequences (eight nucleotides in length), and such data are available for a large number of TFs (Weirauch et al. 2014). These data can therefore be used to describe the architecture of an empirical GP map at high resolution, in which each genotype is a DNA sequence (TF binding site), and the phenotype of this DNA sequence is its ability to bind one or more TFs. These are biologically important phenotypes, because TF binding is integral to the transcriptional regulation of gene expression, which underlies fundamental developmental, behavioral, and physiological processes in species as different as bacteria and humans (Ptashne and Gann 2002). What is more, DNA mutations that affect transcriptional regulation, including those in TF binding sites, may lead to evolutionary adaptations and innovations (Prudhomme et al. 2007; Wray 2007). Examples include binding site mutations that affect body plans in snakes (Guerreiro et al. 2013) and the discrimination of optical stimuli in fruit flies (Rister et al. 2015).

Characterizing the architecture of a GP map helps us to understand how such adaptations and innovations may arise. For example, genotype network structure provides information about how genetic diversity accumulates in an evolving population (van Nimwegen et al. 1999). Combined with an understanding of how genotype networks interface with one another, this information provides insight into how mutations may bring forth new phenotypes. Similarly, by characterizing the overlap of genotype networks with one another, it is possible to study biological phenomena as different as exaptation (Barve and Wagner 2013), plasticity (West‐Eberhard 2003), and multifunctionality (Payne and Wagner 2013). In the context of TF binding sites, such overlap is indicative of “crosstalk,” a phenomenon in which multiple TFs compete for the same binding site, which may lead to incorrect gene activation or repression, as well as the titration of TFs away from their target sites (Friedlander et al. 2016).

In previous work, protein binding microarray data were used to characterize the topologies and topographies of genotype networks of TF binding sites (Payne and Wagner 2014; Aguilar‐Rodríguez et al. 2017). The goals of these studies were to characterize the relationship between robustness and evolvability in TF binding sites (Payne and Wagner 2014), and to understand how mutation and natural selection might navigate such networks toward high‐affinity binding sites (Aguilar‐Rodríguez et al. 2017). To accomplish these goals, the genotype networks of TF binding sites were constructed and studied individually, providing localized characterizations of genotype space. Here, we extend this earlier work by providing a global and more detailed characterization of this genotype space for hundreds of TFs across three kingdoms of life, thus describing the architecture of an empirical GP map at high resolution. Moreover, we do so at two levels of granularity: that of individual TFs and of entire DNA‐binding domain structural classes.

## Materials and Methods

### *IN VITRO* DATA

We studied data from protein binding microarrays (Berger et al. 2006), a chip‐based technology that measures the *in vitro* binding preferences of a TF to all possible 32,896 double‐stranded DNA sequences of length eight. There are $\frac{4^{8}-4^{4}}{2}+4^{4}=32,896$ such sequences, because each is merged with its reverse complement and because there are 4^4^ sequences that are identical to their reverse complement and therefore cannot be merged. We refer to these DNA sequences as TF binding sites (or simply “sites”) because we study the capacity of these sequences to bind TFs. The binding preferences of a TF are reported as a list of protein binding microarray enrichment scores (*E*‐scores), one per binding site (Berger et al. 2006). The *E*‐score is a nonparametric, rank‐based variant of the Wilcoxon–Mann–Whitney statistic that ranges from −0.5 to 0.5. It correlates with a TF's relative dissociation constant, and is therefore used as a proxy for relative binding affinity (Berger et al. 2006; Badis et al. 2009). We used this proxy to discriminate sites that specifically bind a TF via hydrogen bond donors and acceptors (*E*‐score > 0.35), from unbound sites or sites that bind a TF non‐specifically, for example via the TF's affinity for the DNA backbone (*E*‐score ⩽ 0.35). We chose the threshold τ=0.35, which has been used in previous studies (Nakagawa et al. 2013; Payne and Wagner 2014; Aguilar‐Rodríguez et al. 2017), because it corresponds to a low false discovery rate (below 0.001 for 104 mouse TFs (Badis et al. 2009)). To assess the robustness of our results to this choice of affinity threshold, we also carried out a sensitivity analysis by varying τ within the interval (0.35, 0.45).

We consider 525 TFs from three kingdoms of life: animal, plant, and fungi (Table 1, Table S1). Specifically, we downloaded *E*‐scores from the CIS‐BP database for 86 TFs from *Mus musculus*, 217 TFs from *Arabidopsis thaliana*, and 118 TFs from *Neurospora crassa* (Weirauch et al. 2014). We downloaded *E*‐scores of 104 additional *M. musculus* TFs from from the UniPROBE database (Badis et al. 2009; Newburger and Bulyk 2009). We chose to study these three species because they have more TFs characterized in the CIS‐BP database than any other in their respective kingdoms. The TFs we study collectively represent 45 unique DNA‐binding domains, which can be thought of as distinct biophysical mechanisms by which TFs interact with DNA. A Venn diagram of the DNA‐binding domains in the three species is shown in Fig. S1A. In our dataset, several domains are common to all three species, whereas others are unique to one species. For example, *Homeodomain* TFs are found in all three species, but the family of *Zinc cluster* TFs is exclusive to *N. crassa*. This feature of our dataset provides an opportunity to discern whether the architecture of a GP map is governed by the peculiarities of particular binding domains or by the commonalities of TF–DNA interactions across binding domains. This is particularly useful in the context of TF‐DNA interactions, because the set of TFs studied in a given species partly depends upon the interests of the field (e.g., cancer‐associated TFs in humans vs stress‐responsive TFs in plants). By studying multiple species, we can ameliorate the potential effects of this bias.

**Table 1:** Data analyzed in this study

Species	Number of TFs	Number of DNA‐binding domains
*Arabidopsis thaliana*	217	25
*Neurospora crassa*	118	16
*Mus musculus*	190	25

A Venn diagram of the sites that bind TFs from the three species is shown in Fig. S1B. While many sites bind at least one TF in all three species (21.6%), many others bind TFs from just a single species. Specifically, 8.5%, 13.2%, and 9.6% of sites uniquely bind TFs from *A. thaliana*, *M. musculus*, and *N. crassa*, respectively. The TFs bound by such sites do not preferentially belong to binding domains that are exclusive to a single species (Fig. S1C). In total, 14.4% of the 32,896 sites do not bind any of the TFs in our dataset.

### *IN VIVO* DATA

We studied nucleotide diversity in putative TF binding sites in *Arabidopsis thaliana*. To do so, we gathered digital footprints from a DNase I hypersensitivity assay applied to root tissue (Sullivan et al. 2014). These data demarcate protein‐bound open chromatin regions of the genome at single‐nucleotide resolution, and can therefore be used to predict TF binding sites. We filtered the footprints to only include those that are at least eight nucleotides in length and that overlap the promoter regions of the 27,416 annotated protein‐coding genes in the TAIR10 build of the *A. thaliana* genome, where a promoter is defined as the 500 bp upstream of a gene's transcription start site (Sullivan et al. 2014). This resulted in 123,330 footprints, which range in length from 8 to 40 bps (mean 16.56 bps). We used protein binding microarray data to determine whether the DNA sequence of each footprint has the potential to bind any of the 217 *A. thaliana* TFs. Specifically, for each TF, we determined whether the footprint contained a DNA sequence with an *E*‐score >0.35. If it did, we assigned the sequence to the TF as a putative binding site. If the footprint contained more than one binding site for a TF, we randomly chose one of the sites and assigned it to the TF. The number of binding sites thus assigned to a TF ranged from 142 for the TF LEC2 to 55,167 for the TF HMGA.

### NOMENCLATURE

We consider a *genotype space* of TF binding sites for each of the three species we study. This space comprises the set of all possible 32,896 double‐stranded DNA sequences of length eight. The structure of this space can be described as a network, in which nodes represent TF binding sites and edges connect nodes if their corresponding sites differ by a single small mutation, specifically by a point mutation or by an indel (Payne and Wagner 2014). We refer to this network, which contains all possible genotypes, as Ω. If two nodes are connected by an edge in Ω, we refer to them as *neighbors*.

Within this genotype space, we study a GP map in which each genotype is a DNA sequence (TF binding site), and the phenotype of this DNA sequence is its ability to bind one or more TFs (Payne and Wagner 2014). The set of genotypes with a particular phenotype is a *genotype set*. A single genotype may belong to multiple genotype sets, if the site binds multiple TFs. Each genotype set comprises one or more *genotype networks*, in which nodes are genotypes from the genotype set, and edges connect nodes that differ in a single small mutation, as in Ω. If a genotype set is fragmented into multiple genotype networks (connected components), it is usually the case that one network is much larger than the others (Payne and Wagner 2014; Aguilar‐Rodríguez et al. 2017). We refer to this network as the *dominant genotype network* (Fig. S2).

Genotype networks are subnetworks of Ω, in which all genotypes have the same phenotype. We refer to mutations that do not change the phenotype as *neutral*, and to mutations that do change the phenotype as *non‐neutral*. Thus, neutral mutations define the edges within genotype networks, whereas non‐neutral mutations define the edges between genotype networks, or between a genotype network and unbound sequences. If two nodes are connected by an edge in a genotype network, we refer to them as *neutral neighbors*. We emphasize that we use the term “neutral” with respect to a specific phenotype, knowing full well that such mutations may not be neutral with respect to fitness.

Non‐neutral mutations bridge the genotype networks of distinct phenotypes, thus helping to form the edges of a *phenotype network*. In such a network, each node represents the dominant genotype network of a specific TF, and edges connect nodes if (*i*) the associated genotype networks can be reached from one another by at least one non‐neutral mutation, or (*ii*) these genotype networks share at least one genotype. In the latter case, we also say that the genotype networks *overlap*.

### GENOTYPE NETWORKS

To construct each genotype network of TF binding sites, we followed the same procedure as Payne and Wagner (2014). First, for each TF, we determined the set of sites that bind the TF (*E*‐score >0.35). Second, we used an alignment algorithm to calculate the mutational distance between all pairs of bound sites. Third, we used these mutational distances to define the edges of the genotype network by connecting two sites if they have a mutational distance of one.

We considered two kinds of mutations: point mutations, and indels that shift an entire, contiguous binding site by one base (Fig. S3). Two DNA sequences of length eight can differ by a single point mutation in 3×8=24 different ways, because each of the sequence's nucleotides can mutate into any one of the three other nucleotides (Fig. S3A and B). In addition, there are 4×2=8 possible indels that can separate two DNA sequences of length eight. The reason is that the indels we consider can cause a shift in either the 5′ or 3′ direction, and in both cases the unaligned nucleotide can comprise any one of the four possible bases (Fig. S3C and D). There is therefore a maximum of 24+8=32 single mutations that can separate two DNA sequences of length eight.

We determined the mutational distance between two DNA sequences using the Smith–Waterman alignment algorithm, prohibiting gaps in all alignments. For two sequences *s*_1_ and *s*_2_, we calculated the number of mismatches $m(s_{1},s_{2})$ and $m(s_{1},s_{2}^{{}^{\prime }})$, where $s_{2}^{{}^{\prime }}$ is the reverse complement of *s*_2_. We then took the minimum of $m(s_{1},s_{2})$ and $m(s_{1},s_{2}^{{}^{\prime }})$ as the mutational distance between *s*_1_ and *s*_2_.

### INTRANETWORK MEASURES

We used several measures to characterize the internal structure of genotype networks (Newman 2010). The *diameter* of a genotype network is the longest of the shortest mutational paths between any pair of genotypes. The *characteristic path length* is the average of the shortest paths.

The *clustering coefficient* *c* measures the fraction of a genotype's neighbors that are also neighbors themselves, averaged across all genotypes in a genotype network (Watts and Strogatz 1998). Formally, the clustering coefficient is calculated as

(1) $$c=\frac{1}{n}\sum_{i=1}^{n}\left(\frac{2}{k_{i}\left(k_{i}-1\right)}\sum_{j,k}A_{ij}A_{ik}A_{jk}\right),$$

where *n* is the number of genotypes, $k_{i}$ is the degree of node *i*, *A* is the adjacency matrix of the genotype network, and *j* and *k* are the neighbors of node *i*.

The *degree assortativity* *r* of a genotype network measures the propensity for genotypes with a similar number of neighbors (i.e., vertex degree) to share an edge in a genotype network (Newman 2002). It corresponds to the Pearson correlation coefficient of the degrees of connected nodes, and therefore ranges in value from −1 to 1. When r<0, the network is disassortative; when r=0 it is uncorrelated; and when r>0, it is assortative. Assortativity is calculated as

(2) $$r=\frac{M^{-1}\sum_{i}j_{i}k_{i}-{\left[M^{-1}\sum_{i}\frac{1}{2}\left(j_{i}+k_{i}\right)\right]}^{2}}{M^{-1}\sum_{i}\frac{1}{2}\left(j_{i}^{2}+k_{i}^{2}\right)-{\left[M^{-1}\sum_{i}\frac{1}{2}\left(j_{i}+k_{i}\right)\right]}^{2}},$$

where $j_{i}$ and $k_{i}$ are the degrees of the genotypes at the ends of the *i*th edge, and *M* is the number of edges in the genotype network.

The *route factor* *q* of a genotype network measures the average “directness” of the shortest mutational paths to a target genotype from all other genotypes in the network, relative to the shortest mutational paths to the target in Ω (the network used to describe genotype space). It is calculated as

(3) q=1n−1∑i=1n−1li, target di, target ,

where *n* is the number of nodes in the network, $l_{i,\mathrm{target}}$ is the shortest mutational path between genotype *i* and the target genotype in the genotype network, and $d_{i,\mathrm{target}}$ is the shortest mutational path between genotype *i* and the target genotype in Ω (Gastner and Newman 2006). We used the highest affinity binding site as the target genotype. When q=1, the genotype network is optimally distributed in Ω, in the sense that all paths to the target genotype are the shortest possible paths. When q>1, the genotype network possesses paths to the target genotype that are longer than those in Ω, indicating deviations from an optimal distribution.

### INTERNETWORK MEASURES

We characterized the arrangement of genotype networks in genotype space by measuring overlap and mutation probabilities $\varphi_{qp}$ among all pairs of phenotypes *q* and *p*. We applied these measures at two levels of phenotypic granularity. In the first, the phenotype of a binding site genotype is its ability to bind one or more TFs. In the second, the phenotype of a binding site genotype is its ability to bind at least one TF in a class of TFs that share the same DNA‐binding domain. Regardless of the definition of phenotype, we applied the measures to the corresponding genotype networks in the same way.

The *overlap* $O_{qp}$ of dominant genotype networks $G_{q}$ and $G_{p}$, corresponding to phenotypes *p* and *q*, is defined as

(4) $$O_{qp}=\frac{|S\left(G_{q}\right)\cap S\left(G_{p}\right)|}{\left|S(G_{p})\right|},$$

where $S(G_{q})$ is the set of genotypes in genotype network $G_{q}$, and $\left|S(G_{q})\right|$ is the number of genotypes in this set. Note that overlap is an asymmetric measure due to the normalization factor corresponding to the number of binding sites in $G_{p}$.

The fraction $\varphi_{qp}$ of mutations to binding sites in genotype network $G_{p}$ that create binding sites in genotype network $G_{q}$ is defined as

(5) φqp=1|S(Gp)|∑i∈S(Gp)φq local (i),

where

(6) φq local (i)=niqki,

$n_{i}^{q}$ is the number of neighbors of genotype *i* that have phenotype *q*, and $k_{i}$ is the number of neighbors of genotype *i* in Ω. Thus, φq local (i) is the fraction of genotype *i*'s neighbors that have phenotype *q*. We used equations (5) and (6) to calculate the mutational connectivity $\Phi_{q}$ of the genotype network of phenotype *q* from the genotype networks of all other phenotypes in genotype space as

(7) $$\Phi_{q}=\sum_{p}\varphi_{qp}.$$

The measure $\varphi_{qp}$ is similar to the phenotypic accessibility $A_{qp}$ of phenotype *q* from phenotype *p*, which is measured as

(8) $$A_{qp}=\frac{|S\left(G_{q}\right)\cap \partial S(G_{p})|}{|\partial S(G_{p})|},$$

where $S(G_{q})$ is the set of genotypes in the dominant network of phenotype *q* and $\partial S(G_{p})$ is the set of 1‐mutant neighbors of the set $S(G_{p})$ (Stadler et al. 2001; Cowperthwaite et al. 2008). We computed this measure as a point of comparison with $\varphi_{qp}$.

We complemented these global internetwork comparisons by comparing the phenotypic compositions of the local mutational neighborhoods of genotype pairs (i,j), using the Bhattacharyya coefficient (Greenbury et al. 2016):

(9) BC(i,j)=∑qφq local (i)×φq local (j).

This coefficient quantifies the overlap of two distributions and therefore ranges from a minimum of zero when the phenotypic compositions of the mutational neighborhoods of genotypes *i* and *j* are maximally dissimilar to a maximum of one when they are identical. To quantify whether the phenotypic compositions of mutational neighborhoods are more similar among pairs of genotypes (i,j) that are neutral neighbors than among pairs of genotypes (i,k) that are not neutral neighbors, but are from the same genotype network, we computed the *similarity ratio* of the Bhattacharyya coefficients BC(i,j)/BC(i,k). A ratio greater than 1 indicates that the phenotypic compositions of mutational neighborhoods of pairs of genotypes are more similar if those genotypes are connected by a neutral mutation than if they are not, and vice versa. Neighbors that are shared among genotypes *i* and *j*, and among *i* and *k*, were excluded from this analysis to provide a more conservative measure.

### NULL MODEL

We compared our intra‐ and internetwork measures to those from GP maps constructed using the null model of Payne and Wagner (2014). Specifically, we randomly reassigned binding sites to TFs, such that the number of binding sites per TF did not change. We then constructed genotype networks from the reassigned binding sites, and calculated the intra‐ and internetwork measures. We repeated this process 1000 times for the GP maps of the three species that we study.

### SHANNON'S DIVERSITY INDEX

We assessed the amount of nucleotide diversity in each of a TF's putative binding sites in extant *A. thaliana* populations, using single nucleotide polymorphism data from the 1001 Genomes project (1001 Genomes Consortium 2016). Specifically, we calculated Shannon's diversity index *D* of each binding site as

(10) $$D=-\frac{1}{8}\sum_{i=1}^{8}\sum_{j\in A,C,G,T}p_{ij}\log_{2}\left(p_{ij}\right),$$

where $p_{ij}$ is the frequency of allele *j* at position *i* in the binding site. This measure takes on its minimum value of 0 when there is no diversity in the binding site. It takes on its maximum value of 2 when each of the four nucleotides occurs with equal frequency in all eight positions of the binding site.

### DETERMINING THE NUMBER OF TRANSCRIPTION FACTORS PER DNA‐BINDING DOMAIN CLASS

We compared the number of TFs per DNA‐binding domain in our dataset to the same number in the proteomes of *A. thaliana* (UP000006548), *N. crassa* (UP000001805), and *M. musculus* (UP000000589), which we obtained from UniProt (The UniProt Consortium 2015). To find the number of proteins in each proteome with a match to a DNA‐binding domain, we employ the program hmmsearch from the software package HMMER (v3.1b2) (*http://hmmer.org/*). We used a cutoff of 0.01 for both the sequence e‐value and the domain conditional e‐value. We downloaded the hidden Markov models of each DNA‐binding domain from the Pfam database (v27.0) (Finn et al. 2014).

### STATEMENT ON REAGENT AND DATA AVAILABILITY

All data used in this study are freely available in the UniPROBE and CIS‐BP databases. Table S1 provides the necessary information to retrieve these data.

## Results

### GENOTYPE SPACE

We begin with a description of the network of all genotypes Ω, as this is the substrate of the genotype networks that we study in the subsequent sections. This network comprises 32,896 nodes and 523,728 edges. Its degree distribution is shown in Fig. S1D. The vast majority (96%) of genotypes have 32 neighbors, indicating that the network is nearly regular. The remaining 4% of sites possess peculiar features that are detailed in the Supplementary Material. Its diameter—the longest of the shortest paths between any two nodes—is eight, which corresponds to the maximum alignment distance between two sites. On average, however, pairs of TF binding sites are separated by only 4.385 mutations. The clustering coefficient of the network is 0.122, indicating that very few of a site's neighbors are neighbors themselves. This occurs because a site's neighbors can only be neighbors themselves if they differ in the same nucleotide position. For example, the sequence ATATATAT has the neighbors ATATATAA and ATATATAG, which are neighbors themselves, but it also has neighbors such as CTATATAT and ACATATAT, which cannot be neighbors because they differ in two nucleotide positions. The network also lacks any meaningful assortativity by degree (indicated by an assortativity value of r=0.006), which can be attributed in part to the low variance of the degree distribution.

### INTRANETWORK ANALYSES

We first make some general observations about sets of genotypes that bind different TFs. The sizes of these genotype sets vary both within and across species, from a minimum of two sites for the *A. thaliana* TF Abf3 to 1186 sites for the *M. musculus* TF Sp110. Across the three species, the average genotype set size is 374 sites. A total of 53% of these genotype sets comprise a single genotype network, whereas the remaining 47% comprise between 2 and 15 genotype networks. Despite such fragmentation, for 90% of the TFs, more than 95% of the genotype set belongs to the dominant genotype network (Table S1). We therefore carry out all of our analyses on the dominant genotype networks, as in previous work (Payne and Wagner 2014; Aguilar‐Rodríguez et al. 2017). To simplify the presentation of our results, we focus on data from *M. musculus* in the main text, as it is representative of the data from *A. thaliana* and *N. crassa*, which we present in the Supplementary Material.

**Figure 1:**
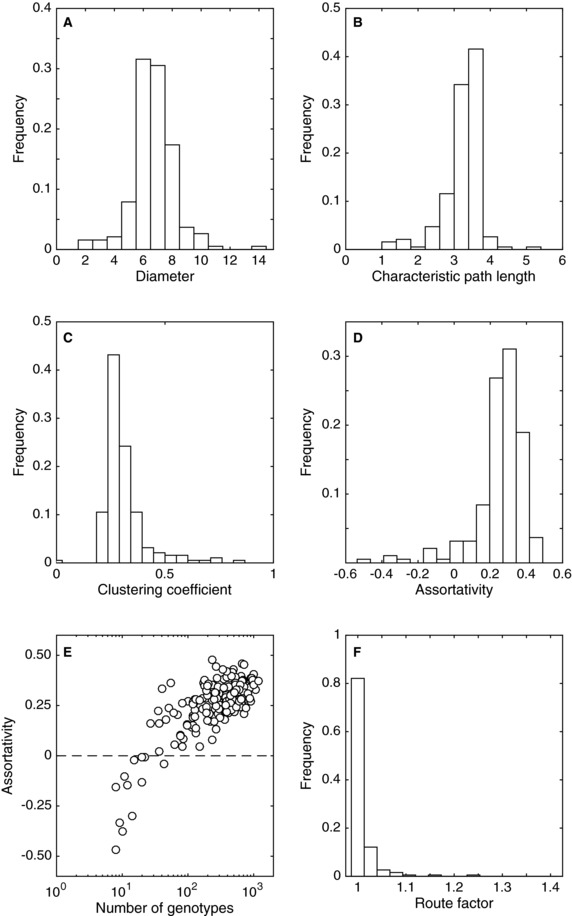
**Intranetwork statistics for 190 TFs from** *M. musculus*. The distributions of genotype network (A) diameter, (B) characteristic path length, (C) clustering coefficient, and (D) assortativity. (E) Assortativity (vertical axis) and its relationship to the number of genotypes in the dominant genotype network (horizontal axis). The horizontal dashed line indicates an uncorrelated (nonassortative) mixing pattern. (F) The distribution of the genotype network route factor.

For the 190 *M. musculus* TFs, the average genotype network diameter is 6.7, varying from a minimum of 2 to a maximum of 14 (Fig. 1A; Table S1). In contrast, the characteristic path length—i.e., the average shortest distance between any pair of genotypes in a genotype network—is 3.2, less than half of the average network diameter (Fig. 1B; Table S1). These genotype networks are highly clustered, with an average clustering coefficient of 0.312 (Fig. 1C; Table S1). Taken together, the short characteristic path length relative to the diameter, and the high clustering coefficients, indicate that genotype networks of TF binding sites tend to fall within the family of “small world” networks (Watts and Strogatz 1998). These are networks that can be traversed in very few steps, like a random network, yet are highly clustered, like a regular lattice. In the context of TF binding sites, the “small world” property has two implications. First, it implies that binding sites are highly evolvable, because only very few mutations are required to travel across the network and potentially access new binding phenotypes. Second, it implies that binding sites are mutationally robust, because they may accumulate multiple mutations and still bind their cognate TF. Qualitatively similar results are obtained for the *A. thaliana* and *N. crassa* TFs (Figs. S4A– C and S5A– C), indicating the consistency of these properties across three branches of the tree of life.

A recent numerical study suggests that assortativity (*r*) may influence evolutionary dynamics on genotype networks (Manrubia and Cuesta 2015). This measure, which ranges from −1≤r≤1, captures the propensity with which nodes of similar degree connect with one another (Newman 2002). Evolutionary dynamics on genotype networks that are assortative by degree (r>0) may result in *phenotypic entrapment*, where the probability that an evolving population leaves a genotype network decreases with the time spent on it (Manrubia and Cuesta 2015). We find that most genotype networks exhibit a moderate amount of degree assortativity, possessing on average a value of r=0.25 (Fig. 1D; Table S1). Degree assortativity is positively correlated with the size of the dominant genotype network (Spearman's r=0.57, $P=1.33\times 10^{-17}$), such that disassortative genotype networks (r<0) are always small (Fig. 1E). This likely reflects finite‐size effects. Figures S4D and E and S5D and E show that the same trends also exists in *A. thaliana* and *N. crassa* TFs. Assortativity is also positively correlated with characteristic path length (Spearman's r=0.56, $P=3.47\times 10^{-19}$), indicating that as genotype networks become less “small‐world,” the potential for phenotypic entrapment increases. Finally, we emphasize that these trends in assortativity do not simply arise from the assortativity of Ω, because Ω shows very little assortativity (r=0.006).

We next describe the structure of genotype networks using the *route factor* *q*. Figure 1F shows the distribution of *q* for the dominant genotype networks of the 190 *M. musculus* TFs, where the target genotype is chosen to have the highest *E*‐score. The distribution is heavily skewed toward q=1, with an average route factor of q=1.01 (Table S1). This indicates that genotype networks of TF binding sites are almost optimally distributed in Ω, meaning that almost all of the mutational paths in a genotype network that lead to the highest affinity sequence are the shortest possible mutational paths. Indeed, 38% of the genotype networks are optimally distributed, with q=1. These results are consistent across the three species we study, as shown in Figs. S4F and S5F.

Figures S6– S8 show that these intranetwork statistics consistently differ from the null expectation. Specifically, the genotype networks constructed from the empirical data have longer diameters and characteristic path lengths, but shorter route factors, as well as higher clustering coefficients and assortativity values than the genotype networks constructed using the null model. Thus, the “small‐worldness” of these genotype networks, as well as their efficient layout in genotype space, and assortative mixing patterns, are not expected structural properties according to the null model. Figures S9– S11 show how these intranetwork statistics change as the binding affinity threshold is increased. In sum, they become more small‐world (i.e., their characteristic path length decreases and their clustering coefficient increases) and slightly less assortative, with a more efficient layout in genotype space. We also study the community structure of these genotype networks, but it is not clear whether and how the network partitions we detect relate to dual modes of binding specificity (Badis et al. 2009), or to other facets of TF‐DNA interactions (Supplementary Material).

**Figure 2:**
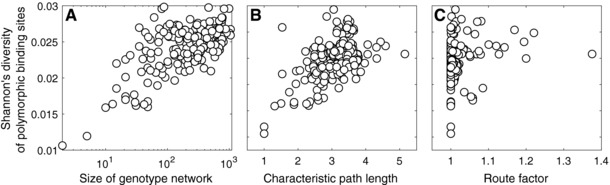
**The structural properties of genotype networks are indicative of binding site diversity in extant populations of** *A. thaliana*. Shannon's diversity of a TF's polymorphic binding sites is shown in relation to (A) the number of nodes, (B) characteristic path length, and (C) route factor of its genotype network. The label of the y‐axis applies to all panels.

Finally, we ask what these intranetwork measures tell us about the evolution of TF binding sites. In particular, we test a series of hypotheses about how the structural properties of genotype networks impact binding site diversity. To do so, we focus on *A. thaliana*, because two important sources of data are available for this species: Digital footprints from DNase I hypersensitivity assays (Sullivan et al. 2014), which can be used to predict TF binding sites, and high‐quality single nucleotide polymorphism data (1001 Genomes Consortium 2016), which can be used to measure binding site diversity in extant populations. Our first hypothesis is that binding site diversity will increase as the number of binding sites in a genotype network increases. Our reasoning is that there are simply more sequences capable of binding a TF in a large genotype network than in a small genotype network, so these binding sites should exhibit more diversity. We find that this is indeed the case. The diversity of polymorphic TF binding sites exhibits a strong positive correlation with the size of the TF's genotype network (Fig. 2A; Spearman's correlation $\rho =0.42,P=1.71\times 10^{-10}$). The second hypothesis is that binding site diversity will increase as the characteristic path length of a genotype network increases. Our reasoning is that genotype networks with large characteristic path lengths are more “spread out” in genotype space and will therefore permit the accumulation of more diversity than genotype networks with short characteristic path lengths. To test this hypothesis, we need to control for genotype network size, because this is positively correlated with characteristic path length (Spearman's correlation $\rho =0.76,P=4.46\times 10^{-42}$). We find that even after controlling for genotype network size, binding site diversity increases with characteristic path length (Fig. 2B; Spearman's partial correlation $\rho =0.23,P=6.38\times 10^{-4}$). The third hypothesis is that binding site diversity will increase as the route factor increases. Our reasoning is that genotype networks with high route factors are less “efficient” than those with short route factors, meaning that there are more sequences in the shortest paths between the highest affinity sequence and other sequences in the network. This reduced efficiency should result in greater binding site diversity, which is indeed what we observe, even after controlling for genotype network size (Fig. 2C; Spearman's partial correlation $\rho =0.21,P=1.70\times 10^{-3}$). The final hypothesis is that binding site diversity will decrease as assortativity increases. Our reasoning is that highly connected nodes are “visited” more frequently by a population evolving on a genotype network (van Nimwegen et al. 1999), and in highly assortative networks, such nodes preferentially connect to one another, which may make it difficult for an evolving population to escape the network's dense core, and thus lead to reduced diversity. However, after controlling for genotype network size, we find no evidence for this phenomenon (Spearman's partial correlation P=0.09), possibly because the genotype networks we study exhibit limited variation in assortativity (−0.47≤r≤0.51 as compared to the full range of −1≤r≤1). Taken together, these analyses indicate that several of the structural properties of genotype networks affect the evolution of TF binding sites *in vivo*, particularly the extent to which binding sites accumulate genetic diversity.

### INTERNETWORK ANALYSES

We now shift the scale of our analysis from local to global, transitioning from descriptions of individual genotype networks to descriptions of how these genotype networks overlap and interface with one another in Ω.

#### Overlap

**Figure 3:**
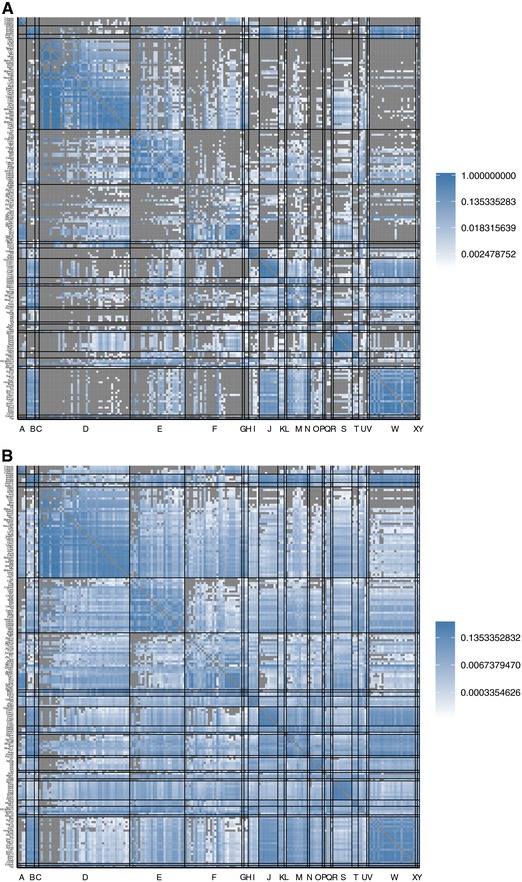
**Matrices of internetwork relationships for the genotype networks of TF binding sites from** *M. musculus*. Heatmaps of log10‐transformed (A) overlap and (B) $\varphi_{qp}$, the probability of mutating from the genotype network of phenotype *p* to the genotype network of phenotype *q*. The rows and columns are grouped according to binding domain, which are ordered alphabetically on the horizontal axis: A, AP‐2; B, ARID/BRIGHT; C, AT hook; D, bHLH; E, bZIP; F, C2H2 ZF; G, CxxC; H, E2F; I, Ets; J, Forkhead; K, GATA; L, GCM; M, Homeodomain; N, Homeodomain + POU; O, IRF; P, MADS box; Q, Myb/SANT; R, Ndt80/PhoG; S, Nuclear receptor; T, RFX; U, SAND; V, SMAD; W, Sox; X, T‐Box; Y: TBP. Within the DNA‐binding domain groups, the rows and columns are ordered by the size of each TF's dominant genotype network, such that network size increases from top to bottom and from left to right. Labels on the vertical axis indicate the name of the TFs, which can be read on the computer by zooming in. Cells colored in gray indicate either N/A values (on the diagonal) or values equal to zero (off‐diagonal).

Some TFs have similar binding preferences, especially if they are products of duplicate (paralogous) genes (Badis et al. 2009; Weirauch et al. 2014). The genotype networks of such TFs will therefore overlap, which has potential implications for TF crosstalk (Friedlander et al. 2016). Figure 3A shows the overlap for all pairs (p,q) of TFs in the mouse dataset. Rows and columns correspond to individual TFs, and are arranged by DNA‐binding domain. The shading of matrix elements depicts overlap as the fraction of binding sites that are common to the genotype networks of two TFs. The matrix is asymmetric, because overlap is normalized by the genotype network size of TF *q*. Similar values of overlap are found in *A. thaliana* and *N. crassa* (Figs. S12A, S13A).

Paralogous TFs exhibit a high level of overlap in their genotype networks, as indicated by the block structure of the main diagonal in Fig. 3A. Even TFs with a *C2H2 ZF* binding domain, which exhibit the lowest levels of overlap, still share 9.14% of their binding sites on average. At the other end of the spectrum are two TFs with an *E2F* binding domain (E2F2 and E2F3), which share 92.73% of their binding sites. Overlap is not restricted to TFs from the same binding domain, as indicated by the blue shading off the main diagonal. For example, *ARID/BRIGHT* and *Sox* TFs share on average 16.5% of their binding sites. In fact, every single TF in the *M. musculus* dataset exhibits overlap in its genotype network with at least one other TF from a different binding domain. TFs with the same DNA‐binding domain tend to share on average 27.2% of their binding sites, while TFs with different binding domains only share 1.88% on average (Wilcoxon rank‐sum test, $P<10^{-6}$). Figure S14 compares genotype network overlap in the empirical data to the null model, and Fig. S15 shows that overlap gradually decreases as the binding affinity threshold increases. In sum, these results suggest that cognate and noncognate TFs may often compete for the same binding sites, especially if the TFs are paralogs.

#### Interface

To characterize how the genotype networks of TF binding sites interface with one another, we calculated the fraction $\varphi_{qp}$ of mutations to binding sites in the genotype network of TF *p* that create binding sites in the genotype network of TF *q* (Greenbury et al. 2016). The matrix in Fig. 3B shows $\varphi_{qp}$ for all TFs in the mouse dataset. It is arranged as in Fig. 3A. Similar values of $\varphi_{qp}$ are found in *A. thaliana* and *N. crassa* (Figs. S12B, S13B).

Of the 35,910 pairwise comparisons depicted in Fig. 3B, 31,548 (87.9%) have $\varphi_{qp}>0$ (as compared to 33.2% in the null model). This means that genotype networks of TF binding sites interface with one another to such an extent that it is usually possible to evolve at least one of a TF's binding sites via a single small mutation to a binding site of nearly any other TF. On average, the $\varphi_{qp}$ between the genotype networks of TFs with the same binding domain is higher than that of TFs with different binding domains (0.139 compared to 0.016; Wilcoxon rank‐sum test, $P<10^{-6}$). Figure S17 compares $\varphi_{qp}$ in the empirical data to that of the null model, and Fig. S18 shows how $\varphi_{qp}$ decreases as the binding affinity threshold increases.

Some pairs of TFs with different binding domains have high $\varphi_{qp}$. For example, the genotype networks of TFs with a *SAND* binding domain have a higher $\varphi_{qp}$, on average, with the genotype networks of TFs with a *bZIP* binding domain than they do with the genotype networks of TFs with the same binding domain. To investigate this further, we compare $\varphi_{qp}$ to the null expectation (Payne and Wagner 2014), which is equivalent to the fraction $f_{q}$ of genotypes with phenotype *q* (Schaper and Louis 2014; Greenbury et al. 2016). We consider the TF Hes7 as phenotype *q*, because it has the largest genotype network of the TFs with a bHLH binding domain, which is the domain with the largest number of TFs in our dataset. We find that the null model does not provide a reasonable approximation to the empirical data (Fig. S16), in contrast with earlier observations in computational models of GP maps (Greenbury et al. 2016). This means that the overall frequency of a phenotype—i.e., the fraction of genotypes with that phenotype—is not a good indicator of the probability that a randomly chosen non‐neutral mutation leads to that phenotype. We find that for TFs with the same binding domain as the focal TF Hes7, $\varphi_{qp}$ is typically larger than the null expectation (Fig. S16, filled circles). Since such TFs often bind similar sets of sites (Weirauch et al. 2014), this observation corroborates the intuition that their genotype networks interface more than expected by chance. However, such TFs do not fully account for the observed deviation from the null model, because removing them from the linear fit of $\varphi_{qp}$ to $f_{q}$ barely improves the coefficient of determination ($R^{2}=0.1337$, as compared to $R^{2}=0.082$). In sum, the arrangement of genotype networks in Ω deviates substantially from the null expectation, and this deviation is not explained by TF paralogs binding similar sets of sequences; even the arrangement of genotype networks of nonparalogous TFs deviates from the null expectation.

GP maps often exhibit correlations in their local mutational neighborhoods (Greenbury et al. 2016). We therefore sought to determine if the composition of such neighborhoods—in terms of the phenotypes that occur in them—might deviate from the null expectation. To do so, we compared the composition of the mutational neighborhoods of pairs of neighboring genotypes on a genotype network to the mutational neighborhoods of randomly selected pairs of nonneighboring genotypes from the same genotype network, removing neighbors that are shared by the genotypes being compared. We used this comparison to compute a similarity ratio that is greater than unity when neighboring genotypes have more similar sets of phenotypes in their mutational neighborhoods than do nonneighboring genotypes (Greenbury et al. 2016). Figure S19 shows a histogram of this similarity ratio for all possible pairs of neighboring genotypes in the genotype network for the mouse TF Sp110, which we have chosen to exemplify this result because it has the largest genotype network in the *M. musculus* dataset. The mean is 1.465 ± 0.006, which deviates significantly from the null expectation of unity (one‐sample *t*‐test, *t* = 79.87, $P<10^{-6}$). We made similar observations in the *A. thaliana* and *N. crassa* data (Figs. S20, S21). Figure S22 shows that the similarity ratio is higher than expected under the null model, and Fig. S23 shows that although the similarity ratio decreases as the binding affinity threshold increases, it always remains above unity.

So far, we have only considered how genotype networks interface with one another. Since mutations that abrogate TF binding are also important for regulatory evolution (Guerreiro et al. 2013), we now turn our attention to the interface of genotype networks with the regions of Ω that do not bind any TF. Such unbound regions are not small: They comprise 51%, 48%, and 39% of Ω in *A. thaliana*, *N. crassa*, and *M. musculus*, respectively. For each TF *P*, we calculate the fraction φ unbound ,p of mutations to binding sites in the genotype network for TF *p* that create unbound sites—i.e., sites that do not bind any TF in our dataset, for the respective species. We then divide this number by the fraction *f*_unbound_ of unbound sites, which is the null expectation for φ unbound ,p (Greenbury et al. 2016). Thus, this ratio will equal unity when the empirical data is well represented by the null model. Figure S24 shows this ratio for all of the mouse TFs. It is consistently below unity. This indicates that unbound sites occur less frequently in the mutational neighborhoods of bound sites than is expected under the null model. Thus, the interface of genotype networks with unbound sites in Ω is qualitatively different from the interface of genotype networks with one another. We made similar observations in the *A. thaliana* and *N. crassa* data (Figs. S25, S26), and these findings are insensitive to the binding affinity threshold (Fig. S27).

Finally, we sum across the columns of Fig. 3B to obtain a global measure $\Phi_{q}$ of the mutational connectivity of the genotype network of phenotype *q* with the genotype networks of all other phenotypes in genotype space. This measure is related to, and highly correlated with, a popular measure called *phenotypic accessibility* (Stadler et al. 2001; Cowperthwaite et al. 2008) (Spearman's r=0.95, $P<10^{-6}$; Fig. S28). The main difference is that $\Phi_{q}$ accounts for genotype network overlap. We find that $\Phi_{q}$ increases with genotype network size (Spearman's r=0.64, $P<10^{-6}$; Fig. S29), indicating that non‐neutral mutations to TF binding sites are more likely to create binding sites for low‐specificity TFs than for high‐specificity TFs, because low‐specificity TFs have larger genotype networks (Payne and Wagner 2014). We also find that $\Phi_{q}$ increases with genotype network size in *A. thaliana* (Fig. S30) and *N. crassa* (Fig. S31).

#### Phenotype space covering

To further characterize how genotype networks of TF binding sites overlap and interface with one another, we calculated the average fraction of phenotypes found within *n* mutations of each binding site, for each TF. We refer to this measure, which has been introduced in a different context as *shape space covering* (Schuster et al. 1994), as *phenotype space covering*, and we call a phenotype “covered” if it is found within a mutational radius of a genotype. We again use the mouse TF Sp110 to exemplify our findings.

**Figure 4:**
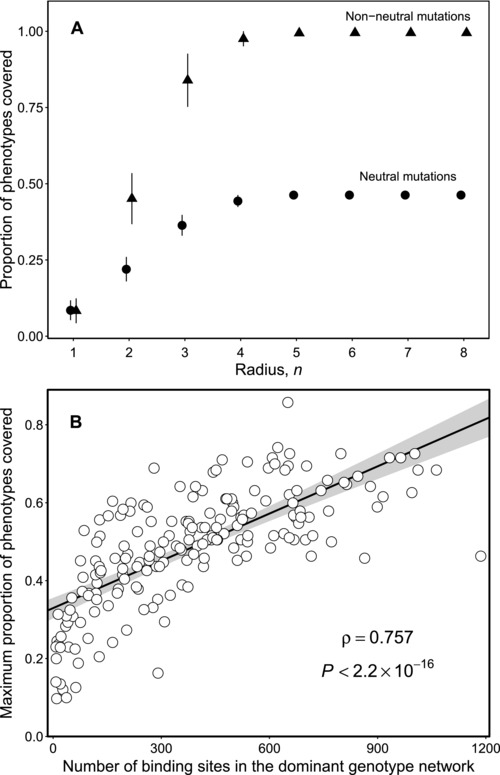
**Phenotype space covering**. (A) The proportion of phenotypes covered as a function of the mutational radius *n* from a given binding site, averaged across all binding sites of the murine TF Sp110. The maximum proportion of phenotypes covered plateaus at a much lower level when considering just neutral mutations than when considering non‐neutral mutations. Error bars are the standard deviations of the mean. (B) The maximum proportion of phenotypes covered by neutral mutations as a function of the number of binding sites in the dominant genotype network, for all 190 murine TFs. The black line shows the fitted linear regression to the data ($R^{2}=0.516$) and the shaded gray area denotes 95% confidence intervals. The figure also shows the Spearman's correlation and its associated *P*‐value.

We consider two variants of phenotype space covering. In the first, we determine the phenotypes of all genotypes within a mutational radius of *n*, such that all mutations are neutral (i.e., the binding sites are part of the same genotype network). This analysis is therefore a further characterization of genotype network overlap. We find for the murine TF Sp110 that within just a single mutation (n=1), an average of 8.51% of the phenotypes are covered, and that within a mutational radius of n>4, a total of 46.31% of the phenotypes are covered (Fig. 4A). The genotype network for Sp110 therefore overlaps with the genotype networks of nearly half of the mouse TFs in our dataset. Figure S32A shows that this does not happen under the null model where phenotype space covering is close to zero for all *n*. We then asked how the maximum proportion of phenotypes covered (e.g., 46.31% for Sp110) relates to the size of a genotype network. Figure 4 B shows that this maximum proportion is largely determined by the size of the dominant genotype network (Spearman's r=0.76, $P<10^{-6}$), such that larger dominant genotype networks cover more phenotypes. Figures S33A –S35A show how this maximum proportion decreases as the binding affinity threshold increases.

In the second variant of phenotype space covering, we consider all genotypes within a mutational radius of *n*, such that all mutations are non‐neutral. The proportion of phenotypes covered within a mutational radius of n=1 does not differ from the first variant, but it increases more rapidly with *n*, such that all phenotypes are covered within a mutational radius of n>4 (Fig. 4A). Figure S32B shows that this increase is reduced under the null model. Moreover, there is no variation in this measure when n>4, meaning that all phenotypes are covered within this mutational radius from any binding site of Sp110. Across all of the mouse TFs, n=4.5 is the average mutational radius for which the coefficient of variance (σ/μ) in the proportion of phenotypes covered becomes smaller than 1%. There are 33 TFs that cover more than 99% of all phenotypes within a radius of n≤4. Remarkably, five of these networks are extremely small, comprising between 8 and 11 binding sites (TFs Arnt2, Fosl1, Hes2, Jun, and Olig3). These binding repertoires are therefore exceptionally evolvable. Figures S33B– S35B show how the radius at which all phenotypes are covered increases as the binding affinity threshold increases.

### GENOTYPE NETWORKS OF DNA‐BINDING DOMAINS

The GP map we study can be analyzed at multiple levels of granularity. We have so far considered a fine‐grained analysis, in which each genotype is a DNA sequence, and the phenotype is the sequence's ability to bind one or more TFs. We now consider a more coarse‐grained analysis, in which each genotype is a DNA sequence, and the phenotype is the sequence's ability to bind at least one TF in a class of TFs that share the same DNA‐binding domain. Studying the overlap and interface of such genotype networks complements our previous analyses by describing how TFs with different binding domains may compete for the same sites, and how DNA mutations may transfer regulatory control from a TF with one DNA‐binding domain to a TF with a different binding domain.

**Figure 5:**
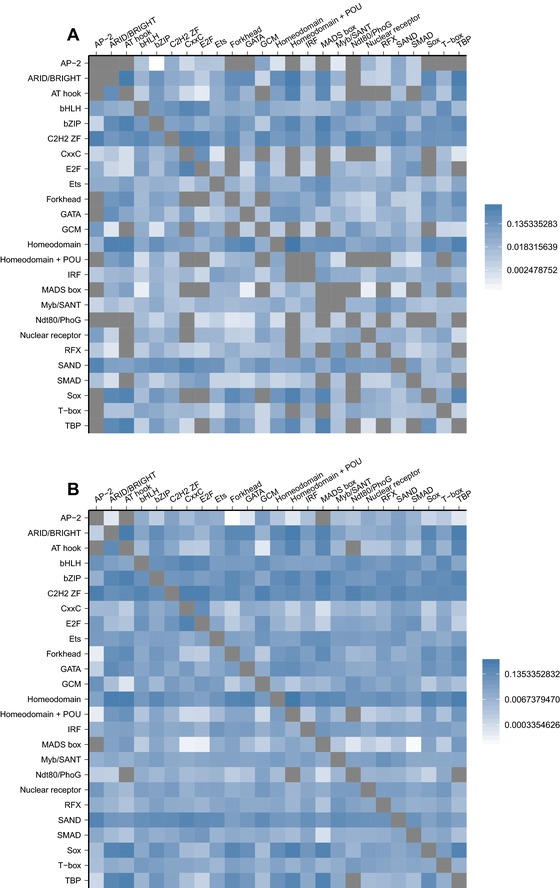
**Matrices of internetwork relationships for the genotype networks of binding domains from** *M. musculus*. Heatmaps of log10‐transformed (A) overlap and (B) $\varphi_{qp}$, the probability of mutating from the genotype network of phenotype *p* to the genotype network of phenotype *q*. Each row and column represents a different genotype network. Domains are ordered alphabetically. Cells colored in gray indicate either N/A values (on the diagonal) or values equal to zero (off‐diagonal).

Figure 5A shows the extent of overlap among all pairs of genotype networks for the 25 DNA‐binding domains in the *M. musculus* dataset. Such overlap is pervasive. For example, there are six binding domains with genotype networks that overlap the genotype networks of every other binding domain in the dataset (*bHLH*, *bZIP*, *C2H2* *ZFs*, *Ets*, *Homeodomain*, *SAND*). Even the *AP‐2* and *Ndt80/PhoG* binding domains, which exhibit the lowest levels of overlap, still overlap with 14 (56%) of the other domains. In total, 504 of the 600 pairs of binding domains exhibit overlap in their genotype networks. It is therefore common for TFs with different binding domains to recognize some of the same sites, further highlighting the potential for crosstalk in transcriptional regulation (Friedlander et al. 2016). Similar patterns hold in *A. thaliana* and *N. crassa* (Figs. S36A, S37A), even though these species have several binding domains that are not present in the *M. musculus* dataset. Such overlap therefore appears to be a general consequence of the low specificity with which eukaryotic TFs interface with DNA, rather than a consequence of the binding preferences of any particular binding domain.

Figure 5B shows $\varphi_{qp}$ for all pairs of the 25 DNA‐binding domains in the *M. musculus* dataset. As with overlap, we observe an increase in $\varphi_{qp}$ as we shift the level of analysis from TFs to DNA‐binding domains. A total of 590 (98.3%) binding domain pairs exhibit nonzero $\varphi_{qp}$. Mutations in TF binding sites could thus commonly transfer regulatory control among TFs with different binding domains. Similar observations are made for *A. thaliana* and *N. crassa* (Figs. S36B, S37B). We also studied how the different genotype networks of DNA‐binding domains interface with one another through the visualization of phenotype networks (Figs. S38– S40).

Finally, $\Phi_{q}$ scales with genotype network size at the level of DNA‐binding domains, just as it did at the level of individual TFs (compare Figs. S41– S43 with Figs. S29– S31). However, since the number of TFs per binding domain in the *M. musculus* dataset also scales with genotype network size (Fig. S44A), we were concerned that these trends may stem from ascertainment bias. This could occur if the number of TFs per binding domain in the *M. musculus* dataset was not representative of the number of TFs per binding domain in the *M. musculus* genome. Figures S44B,C show that this is not the case. Both the number of TFs per binding domain in the *M. musculus* dataset, and the size of the corresponding genotype network, scale with the number of TFs per binding domain in the *M. musculus* genome. We made similar observations in *A. thaliana* and *N. crassa* (Figs. S45, S46).

## Discussion

The concept of a genotype‐phenotype (GP) map can be traced back to the work of Sewall Wright (Wright 1932), Conrad H. Waddington (Waddington 1959), and John Maynard Smith (Maynard Smith 1970). However, the term GP map (“genotype‐phenotype mapping”) was only coined in 1970 by Jim Burns (Burns 1970), who recognized the importance of incorporating a mechanistic perspective into the evolutionary framework of population genetics, thus outlining the research programme that has come to be known as evolutionary systems biology (Soyer and O'Malley 2013). The term was re‐introduced in 1991 by the developmental biologist Pere Alberch (Alberch 1991), who was interested in macroscopic phenotypes arising from complex developmental processes. The study of GP maps is currently shifting away from the conceptual and computational models that shaped the thinking of the founders of the field, toward empirical data derived from high‐throughput assays (Rowe et al. 2010; Fowler et al. 2010; Hinkley et al. 2011; Jiménez et al. 2013; Melamed et al. 2013; Buenrostro et al. 2014; Findlay et al. 2014; Olson et al. 2014; Podgornaia and Laub 2015; Julien et al. 2016; Li et al. 2016; Puchta et al. 2016; Qiu et al. 2016; Sarkisyan et al. 2016). Our study is part of this shift. We have used experimental data from protein binding microarrays to analyze the architecture of an empirical GP map, in which each genotype is a short DNA sequence, and the phenotype of the sequence is its ability to bind one or more TFs. This study expands upon previous analyses of this map (Payne and Wagner 2014; Aguilar‐Rodríguez et al. 2017) by providing more nuanced descriptions of individual genotype networks, detailed characterizations of how these networks overlap and interface with one another, and does so at two levels of phenotypic granularity.

Our analyses of individual genotype networks provide two new insights into their structure. First, they tend to be “small‐world” (Watts and Strogatz 1998), an observation that furthers our understanding of the “robust‐yet‐evolvable” nature of TF binding sites (Payne and Wagner 2014): While binding sites tend to be highly clustered in their genotype network (robustness), it remains possible to traverse the network with just a few mutations, thus providing efficient access to adjacent genotype networks (evolvability). Indeed, the route factor of these genotype networks indicates that they are almost optimally distributed in genotype space, in the sense that almost all genotypes are connected to a central target genotype through the shortest mutational paths. These structural properties have implications for the evolution of TF binding sites. Specifically, we find that they affect the accumulation of genetic diversity in extant populations of *A. thaliana*, such that binding site diversity increases as a genotype network's characteristic path length or route factor increases.

The second new insight is that genotype networks are assortative, meaning that robust (i.e., highly connected) binding sites are likely to neighbor other robust binding sites. The potential implication for the evolution of TF binding sites is reduced diversity, because an evolving population tends to accumulate in such densely connected regions of genotype networks (van Nimwegen et al. 1999), which may lead to “phenotypic entrapment” (Manrubia and Cuesta 2015), a phenomenon in which an evolving population becomes less likely to leave its genotype network the more time it spends on it. However, we do not find evidence of this in our analysis of polymorphism data from *A. thaliana*, inasmuch as we do not find a significant relationship between assortativity and binding site diversity. One possible explanation is that the genotype networks we study exhibit limited variation in assortativity.

A sometimes underappreciated feature of GP maps is that genotypes may have more than one phenotype (West‐Eberhard 2003; Wagner and Zhang 2011), which means that genotype networks may overlap. Even if we restrict our examples to the molecular realm, they are numerous: An RNA transcript can be translated into different proteins (Bratulic et al. 2015), an amino acid sequence can fold into different conformational structures (Bloom et al. 2006), and a promiscuous enzyme can catalyze different reactions (Nam et al. 2012). In the GP map studied here, such overlap is also pervasive, both at the level of individual TFs and of DNA‐binding domains. It implies competition for binding sites among cognate and noncognate TFs, a phenomenon known as “crosstalk.” Recent modeling work suggests that crosstalk is an inevitable feature of transcriptional regulation in species that employ limited‐specificity TFs (Friedlander et al. 2016), such as the three eukaryotic species studied here. This is important because crosstalk places constraints on the function and evolution of transcriptional regulatory networks. Our results provide an empirical complement to these earlier theoretical findings, by providing estimates of how much crosstalk can occur among TFs and binding domains. However, it is worth highlighting that these estimates are based on *in vitro* measurements of TF binding preferences. The myriad complexities of *in vivo* TF–DNA interactions (Siggers and Gordân 2014), including epigenetic marks, local sequence, and chromatin context, as well as interactions with protein partners, will certainly affect these estimates. Our ability to interrogate the effects of these complexities on TF–DNA interactions is continuing to advance (Hu et al. 2013; Levo et al. 2015; Isakova et al. 2017; Levo et al. 2017), and we believe that genotype networks will provide a useful framework for studying how such complexities mitigate crosstalk in transcriptional regulation. Of particular interest is the role played by chromatin silencing (Beisel and Paro 2011), which may mitigate crosstalk by making binding sites unavailable in the presence of noncognate TFs.

Our analysis of how genotype networks interface with one another has implications for the emergence of evolutionary innovations, because mutations in cis‐regulatory regions may produce novel gene expression patterns (Wray 2007; Prudhomme et al. 2007). In particular, single‐base pair mutations in TF binding sites can shift the regulatory control of a gene from one TF to another, and this may cause profound phenotypic change. For example, such mutations led to the differential expression of Rhodopsin genes in different subsets of *Drosophila* photoreceptors (Rister et al. 2015), which facilitated the discrimination of a wide spectrum of optical stimuli, and thus drastically changed how flies perceive their environment. In the GP map studied here, it has been previously shown that genotype networks are so intertwined that it is usually possible to mutate at least one of a TF's binding sites to a binding site of nearly any other TF (Payne and Wagner 2014). This means that mutation can readily shift the regulatory control of a gene from one TF to another, a shift that may lead to an adaptive change in gene expression. Here, we provide a more detailed and nuanced view of TF binding site evolvability. At the most local scale, evolvability is relatively low because neutral neighbors tend to have highly similar mutational neighborhoods, which decreases the diversity of novel phenotypes that may arise via a single point mutation to any one binding site (Greenbury et al. 2016). However, only very few mutations are required to shift regulatory control from the cognate TF to nearly any other TF in our dataset. At even this intermediate scale, TF binding sites are therefore remarkably evolvable.

An important challenge in the biological sciences is to provide a comprehensive description of the architecture of an empirical GP map. The hyper‐astronomical size of genotype space renders this challenge impossible for most biological systems of interest, including macromolecules, regulatory circuits, and metabolisms (Louis 2016). Even for the relatively small genotype space studied here, we fall short of a comprehensive description. The reason is that we do not have data describing the binding preferences of every TF from each of our three study species. However, the data we do have are a representative sampling of each species' TF repertoire. There are two reasons for this. The first is that the assayed TFs were intentionally selected to exhibit an even balance among DNA‐binding domains and to survey different levels of sequence similarity (Weirauch et al. 2014). The second is that the number of TFs per binding domain in our dataset is correlated with the number of TFs per binding domain in the genomes of each species (The UniProt Consortium 2015). This study therefore provides a high‐resolution depiction of the architecture of an empirical GP map.

Associate Editor: J. Jensen

Handling Editor: P. Tiffin

## Supporting information


**Figure S1**. Data.
**Figure S2**. Genotype network of TF binding sites.
**Figure S3**. Two forms of mutation.
**Figure S4**. Intra network statistics for 217 TFs from *A. thaliana*.
**Figure S5**. Intra network statistics for 118 TFs from *N. crassa*.
**Figure S6**. Comparison of intra network statistics to those of a null model for 190 TFs from *M. musculus*.
**Figure S7**. Comparison of intra network statistics to those of a null model for 217 TFs from *A. thaliana*.
**Figure S8**. Comparison of intra network statistics to those of a null model for 118 TFs from *N. crassa*.
**Figure S9**. Intra network statistics with different binding affinity thresholds for 190 TFs from *M. musculus*.
**Figure S10**. Intra network statistics with different binding affinity thresholds for 217 TFs from *A. thaliana*.
**Figure S11**. Intra network statistics with different binding affinity thresholds for 118 TFs from *N. crassa*.
**Figure S12**. Matrices of inter network relationships for the genotype networks of TF binding sites from *A. thaliana*.
**Figure S13**. Matrices of inter network relationships for the genotype networks of TF binding sites from *N. crassa*.
**Figure S14**. Comparison of overlap in the empirical data to that in the null model.
**Figure S15**. Genotype network overlap in relation to the binding affinity threshold.
**Figure S16**. A simple null model does not provide a reasonable approximation to $\varphi_{qp}$.
**Figure S17**. Comparison of phenotype mutation probabilities in the empirical data to those of a null model.
**Figure S18**. Phenotype mutation probabilities in relation to binding affinity threshold.
**Figure S19**. In *M. musculus*, the phenotypes found in the mutational neighborhoods of neutral neighbors are more similar than those of neutral pairs that are not neighbors.
**Figure S20**. In *A. thaliana*, the phenotypes in the mutational neighborhoods of neutral neighbors are more similar than those of neutral pairs that are not neighbors.
**Figure S21**. In *N. crassa*, the phenotypes in the mutational neighborhoods of neutral neighbors are more similar than those of neutral pairs that are not neighbors.
**Figure S22**. Comparison of similarity ratios in the empirical data to those of a null model.
**Figure S23**. Similarity ratios with different binding affinity thresholds.
**Figure S24**. In *M. musculus*, unbound sites are underrepresented in the neighborhoods of bound sites.
**Figure S25**. In *A. thaliana*, unbound sites are underrepresented in the neighborhoods of bound sites.
**Figure S26**. In *N. crassa*, unbound sites are underrepresented in the neighborhoods of bound sites.
**Figure S27**. Unbound sites are underrepresented in the neighborhoods of bound sites for all binding affinity thresholds.
**Figure S28**. Phenotypic accessibility $A_{qp}$ is strongly correlated with $\varphi_{qp}$.
**Figure S29**. In *M. musculus*, the global mutational connectivity of a phenotype increases with the size of its dominant genotype network.
**Figure S30**. In *A. thaliana*, the global mutational connectivity of a phenotype increases with the size of its dominant genotype network.
**Figure S31**. In *N. crassa*, the global mutational connectivity of a phenotype increases with the size of its dominant genotype network.
**Figure S32**. Comparison of phenotype space covering in the empirical data to that in the null model.
**Figure S33**. Phenotype space covering with different binding affinity thresholds for 190 TFs from *M. musculus*.
**Figure S34**. Phenotype space covering with different binding affinity thresholds for 217 TFs from *A. thaliana*.
**Figure S35**. Phenotype space covering with different binding affinity thresholds for 118 TFs from *N. crassa*.
**Figure S36**. Matrices of inter network relationships for the genotype networks of DNA‐binding domains from *A. thaliana*.
**Figure S37**. Matrices of inter network relationships for the genotype networks of DNA‐binding domains from *N. crassa*.
**Figure S38**. Phenotype network for 25 DNA‐binding domains from *M. musculus*.
**Figure S39**. Phenotype network for 25 DNA‐binding domains from *A. thaliana*.
**Figure S40**. Phenotype network for 16 DNA‐binding domains from *N. crassa*.
**Figure S41**. In *M. musculus*, the global mutational connectivity of a phenotype increases with the size of its dominant genotype network.
**Figure S42**. In *A. thaliana*, the global mutational connectivity of a phenotype increases with the size of its dominant genotype network.
**Figure S43**. In *N. crassa*, the global mutational connectivity of a phenotype increases with the size of its dominant genotype network.
**Figure S44**. Binding domains with more TFs have larger genotype networks in *M. musculus*.
**Figure S45**. Binding domains with more TFs have larger genotype networks in *A. thaliana*.
**Figure S46**. Binding domains with more TFs have larger genotype networks in *N. crassa*.
**Table S1**. Information about all the transcription factors analyzed in this study.
**Table S2**. The number of genotype networks that have a partition that exhibits a particular group structure according to a partitioning method based on a stochastic block model.
**Table S3**. The number of genotype networks that have a binding affinity partition that exhibits a particular group structure.
