# Supplementary material for: The architecture of an empirical genotype‐phenotype map
Source: Evolution. 2018 May 25;72(6):1242–60. doi: 10.1111/evo.13487 (PMC6055911; doi:10.1111/evo.13487)
Supplement: Supplementary file 1 — Figure S1. Data. Figure S2. Genotype network of TF binding sites. Figure S3. Two forms of mutation. Figure S4. Intra network statistics for 217 TFs from A. thaliana. Figure S5. Intra network statistics for 118 TFs from N. crassa. Figure S6. Comparison of intra network statistics to those of a null model for 190 TFs from M. musculus. Figure S7. Comparison of intra network statistics to those of a null model for 217 TFs from A. thaliana. Figure S8. Comparison of intra network statistics to those of a null model for 118 TFs from N. crassa. Figure S9. Intra network statistics with different binding affinity thresholds for 190 TFs from M. musculus. Figure S10. Intra network statistics with different binding affinity thresholds for 217 TFs from A. thaliana. Figure S11. Intra network statistics with different binding affinity thresholds for 118 TFs from N. crassa. Figure S12. Matrices of inter network relationships for the genotype networks of TF binding sites from A. thaliana. Figure S13. Matrices of inter network relationships for the genotype networks of TF binding sites from N. crassa. Figure S14. Comparison of overlap in the empirical data to that in the null model. Figure S15. Genotype network overlap in relation to the binding affinity threshold. Figure S16. A simple null model does not provide a reasonable approximation to φqp. Figure S17. Comparison of phenotype mutation probabilities in the empirical data to those of a null model. Figure S18. Phenotype mutation probabilities in relation to binding affinity threshold. Figure S19. In M. musculus, the phenotypes found in the mutational neighborhoods of neutral neighbors are more similar than those of neutral pairs that are not neighbors. Figure S20. In A. thaliana, the phenotypes in the mutational neighborhoods of neutral neighbors are more similar than those of neutral pairs that are not neighbors. Figure S21. In N. crassa, the phenotypes in the mutational neighborhoods of neutral neighbors are more similar than th [file EVO-72-1242-s001.pdf]

Supplementary information

Supplementary methods

The stochastic block model for network partitioning

The stochastic block model (SBM) is a probabilisitic generative model for networks (Holland et al. 1983; Nowicki and Snijders 2001). Under the SBM, all nodes are assigned to one of k groups, and the probability of an edge connecting any pair of nodes depends only upon the nodes' group memberships. The pattern of edges can therefore be described by a single $k \times k$ "mixing matrix," in which each element p_{rs} gives the interaction probability between groups r and s (i.e., the probability that an edge exists between a node from group r and a node from group s.)

Using statistical inference (Peixoto 2014), we determined the maximum likelihood group assignment for each of the nodes in each genotype network. For a given assignment, the maximum likelihood interaction probability between groups r and s is given by the observed number of edges between the groups divided by the number of possible edges between the groups. That is,

$$p_{rs} = \frac{e_{rs}}{n_r n_s}, \tag{11}$$

where e_{rs} is the number of edges connecting nodes in group r to nodes in group s, and n_r and n_s are the number of nodes in groups r and s, respectively. Because we set the number of groups to $k = 2$, we have just three group interaction probabilities p_{11}, p_{12} and p_{22}, because the network is undirected. By comparing these probabilities, we can determine the type of structure the groups represent. For the two-group case there are three possibilities: $p_{11} > p_{12} < p_{22}$ (assortative), $p_{11} < p_{12} > p_{22}$ (disassortative), and $p_{11} > p_{12} > p_{22}$ (core-periphery) (Zhang et al. 2015).

Introduced in Peel et al. (2017), the block model entropy significance test provides a means for identifying whether node attributes are distributed randomly across a network. The test works by partitioning a network into groups of nodes that have the same node attribute value (for continuous-valued attributes, we form groups by discretizing the values into bins). Using this partition $\mathcal{M}$, we calculate the mixing matrix using Eq. (11). As a test statistic, we calculate the SBM entropy $\mathcal{H}$,

$$\mathcal{H}(\mathcal{M}) = -\left[\sum_{rs} e_{rs} \log p_{rs} + (n_r n_s - e_{rs} \log(1 - p_{rs})\right]. \tag{12}$$

High entropy indicates that node attributes are not correlated with network structure. Low entropy indicates that there is a correlation between the node attributes and the network structure. To determine if this correlation is statistically significant, we compare the observed entropy against a null distribution of entropy values. We obtain this distribution by randomly permuting node attributes, resulting in new partitions $\{\pi\}$ and corresponding mixing matrices. Importantly, this choice of null model preserves both the observed network structure and the relative frequencies of attribute values, but removes any correlation between the two. The result is a standard empirical p-value, defined as

$$p = Pr[H(\pi) \leq H(\mathcal{M})]. \tag{13}$$

Smaller p-values indicate a lower plausibility that a random permutation of the node attributes could describe the network structure as well as the observed distribution of node attributes.

Binding affinity partitions

We used the SBM partitions as a baseline for building node partitions that are based on binding affinities. For each genotype network, we attributed a categorical label to every node, indicating its SBM group. We chose "0" for nodes in the most assortative group and "1" for other nodes. This labeling also induces a partitioning of the binding affinities into two groups.

For each genotype network, we performed a logistic regression of the SBM partitioning of binding affinities. Using $b_{\min}$ and $b_{\max}$ to denote the minimum and maximum binding affinity values within a given genotype network, the regression resulted in a classifier $C : [b_{\min}, b_{\max}] \to [0, 1]$ that we trained on the empirical data. This classifier provided the likelihood that a given binding affinity value belonged to one SBM group or the other. In order to distinguish between "high" and "low" binding affinities, we chose the critical value b^* to be the binding affinity at which the classifier distinguished between groups, i.e. $C(b^*) = 0.5$. We used b^* in order to obtain a binding affinity partition, with nodes having binding affinities less than or equal to b^* in a group labeled g_{low}, and nodes with binding affinities greater than or equal to b^* in another group labeled g_{high}.

To test the statistical significance of binding affinity with respect to the structure of a genotype network, we again used the block model entropy significance test, such that in Eqs. 11 and 12, groups r and s were replaced with groups g_{low} and g_{high}.

Table S2. We show the number of genotype networks that have a partition that exhibits a particular group structure according to a partitioning method based on a stochastic block model.

Species	Group structure		
	Core-periphery	Assortative	Disassortative
A. thaliana	1 (0.46%)	213 (98.16%)	3 (1.38%)
N. crassa	1 (0.85%)	117 (99.15%)	0 (0.00%)
M. musculus	1 (0.53%)	186 (97.89%)	3 (1.58%)

Supplementary results

Genotype network partitions

Some TFs exhibit dual modes of binding specificity, such that they have a primary preference to one set of binding sites, as well as a secondary preference to a different set of binding sites (Badis et al. 2009). In some cases, these sets of sequences bind the TF with similar affinity, whereas in others the primary set binds the TF with higher affinity than the secondary set (Badis et al. 2009). These observations motivated us to perform an exploratory analysis of genotype network partitions — distinct groups of nodes that have more edges within them than between them (Zhang et al. 2015) — as these may reflect dual modes of binding specificity, or other nuances of TF-DNA interactions, such as DNA shape readout (Rohs et al. 2009).

To determine if partitions exist for genotype networks of TF binding sites, we took two approaches. In the first, we used a partitioning method that is based on a stochastic block model (Zhang et al. 2015). This method assigns each genotype in a genotype network to one of two groups (labeled g_1 and g_2), and uses a 2×2 "mixing matrix" to describe the structure of the network. This symmetric matrix contains the probabilities of observing edges between genotypes from the same group ($p_{g_1 g_1}$ and $p_{g_2 g_2}$) and between different groups ($p_{g_1 g_2}$). The method uses maximum likelihood to find the partition and mixing matrix that best explain the structure of the genotype network (Materials and Methods). The resulting probabilities of the mixing matrix can be used to classify each genotype network as exhibiting an assortative group structure ($p_{g_1 g_1} > p_{g_1 g_2} < p_{g_2 g_2}$), a disassortative group structure ($p_{g_1 g_1} < p_{g_1 g_2} > p_{g_2 g_2}$), or a core-periphery group structure ($p_{g_1 g_1} > p_{g_1 g_2} > p_{g_2 g_2}$) (Zhang et al. 2015). This approach is related to modularity optimization, and even produces the same results under certain conditions — specifically when the partitions are assortative and the edge densities of the groups are the same (Newman 2016). In contrast, the stochastic block model faciltates the discovery of a broader

range of significant partitions, including core-periphery and disassortative group structures, in addition to assortative group structures.

We find that the vast majority of genotype networks in the mouse dataset (97.9%) exhibit an assortative group structure (Table S2). Thus, not only are these networks globally assortative by degree ($r > 0$, Fig. 1C), they are also partitionable into two groups that each have more edges within them than between them. The same is true for the *A. thaliana* and *N. crassa* TFs, of which 98% and 99% exhibit an assortative group structure, respectively (Table S2).

We next asked whether similar trends in group structure exist if we manually partition each genotype network according to binding affinity, rather than relying on the maximum likelihood approach described above. Our motivation is that TFs with dual modes of binding specificity sometimes bind the sequences in the primary set with higher affinity than those in the secondary set (Badis et al. 2009). We used the structural partition of each genotype network into the two groups g_1 and g_2 to find an affinity threshold that best separates the binding affinities of these groups (Materials and Methods). We used this threshold to label the genotypes as belonging to a high-affinity group g_{high} or to a low-affinity group g_{low}. We then constructed a mixing matrix that contains the probabilities of observing edges within groups ($p_{g_{\mathrm{low}},g_{\mathrm{low}}}$ and $p_{g_{\mathrm{high}},g_{\mathrm{high}}}$) and between groups ($p_{g_{\mathrm{low}},g_{\mathrm{high}}}$), calculated directly from each genotype network. We used this mixing matrix to test the null hypothesis H_0 that binding affinity is distributed uniformly at random with respect to the structure of the genotype network (Materials and Methods) (Peel et al. 2017). Thus, rejection of H_0 indicates that the binding affinity partition provides meaningful information about genotype network structure. Table S3 shows that H_0 is almost always rejected. On the rare occasion that H_0 is accepted, the genotype network is small (≤ 72 nodes), which again likely indicates finite-size effects. Additionally, we find in *M. musculus* that 62.1% of the binding affinity partitions exhibit a core-periphery group structure ($p_{g_{\mathrm{high}},g_{\mathrm{high}}} > p_{g_{\mathrm{high}},g_{\mathrm{low}}} > p_{g_{\mathrm{low}},g_{\mathrm{low}}}$), while 34.7% exhibit an assortative group structure ($p_{g_{\mathrm{high}},g_{\mathrm{high}}} > p_{g_{\mathrm{high}},g_{\mathrm{low}}} < p_{g_{\mathrm{low}},g_{\mathrm{low}}}$). Similar results are obtained for the *A. thaliana* and *N. crassa* TFs (Table S3). In sum, genotype networks of TF binding sites can be partitioned in multiple meaningful ways, and the resulting group structure depends upon how the partition is defined. An assortative group structure is uncovered by a structure-based partition, whereas a core-periphery group structure can be uncovered by an affinity-based partition. Whether and how these partitions relate to dual modes of binding specificity, or to other facets of TF-DNA interactions, requires further investigation. It is our hope that by making these partitions publicly available, other researchers may use them to improve

Table S3. The number of genotype networks that have a binding affinity partition that exhibits a particular group structure. We also test the null hypothesis H_0 that binding affinity is random with respect to genotype network structure, rejecting H_0 if $p < 0.05$.

Species	Group structure			H_0 rejections
	Core-periphery	Assortative	Disassortative	
A. thaliana	134 (61.75%)	79 (36.41%)	4 (1.84%)	209 (96.31%)
N. crassa	67 (56.78%)	49 (41.53%)	2 (1.69%)	113 (95.76%)
M. musculus	118 (62.11%)	66 (34.74%)	4 (2.11%)	181 (95.26%)

our understanding of TF binding specificity, perhaps via the development of more sophisticated binding models.

Some sequences have fewer than 32 neighbors in genotype space

Of the 32,896 sequences in genotype space, 1,312 have fewer than 32 neighbors (Fig. S1D). This occurs when two different mutations to a sequence yield the same mutated sequence, forcing the prioritization of one mutation over another. If the mutations are of different types (i.e., a point mutation and an indel), we always prioritize the point mutation over indels because laboratory evolution experiments indicate that they occur more frequently than indels (Cartwright 2009; Chen et al. 2009).

The 1,312 genotypes with fewer than 32 neighbors fall into the following five groups:

1. There are $4^4 = 256$ sequences that are identical to their reverse complements, and 252 of these have 16 neighbors. Due to the symmetry of these sequences, the number of possible point mutations in them is reduced from 24 to 12. The reason is that a point mutation in position $i \leq 4$ is equivalent to a point mutation to the Watson-Crick pair in position $8-i+1$, after taking the reverse complement. For example, consider the point mutation `A` $\rightarrow$ `C` in the 1st position of `ACGTACGT`. This yields the same sequence (`CCGTACGT`) as a point mutation `T` $\rightarrow$ `G` in the 8th position, after taking the reverse complement of the mutated sequence. The symmetry of these sequences also reduces the number of possible indels from 8 to 4. For example, an indel separates the sequence `ACGTACGT` from `CGTACGTA`, such that an alignment will leave the 1st position of the former sequence and the 8th position of the latter sequence unaligned. An indel also separates the sequence `ACGTACGT` from `TACGTACG`, such that an alignment will leave the 8th position of the former sequence and the 1st position of the latter sequence unaligned. Since the sequences `CGTACGTA` and `TACGTACG` are reverse complements of one another, it is not possible for `ACGTACGT` to have both of these mutational neighbors. In sum,

these 252 sequences only have $12 + 4 = 16$ neighbors.

2. Of the 256 sequences that are identical to their reverse complements, four have 15 neighbors: AAAATTTT, CCCCGGGG, GGGGCCCC, TTTTAAAA. The reasons are the same as for the other 252 sequences, except that the number of possible indels is further reduced to 3. To understand why, consider aligning the sequence AAAATTTT with AAATTTTT. This alignment could either include a point mutation in the 4th position, or an indel that leaves the 1st position of the former sequence and 8th position of the latter sequence unaligned. For this reason, these four sequences have 15 neighbors.

3. There are two sequences with 24 neighbors: AAAAAAAA and CCCCCCCC. They have 24 neighbors because we prioritize point mutations: We consider that any mutation that might be caused by an indel is more likely to be caused by a point mutation.

4. There are 46 sequences with 30 neighbors. 41 of these are of the form AAAAAAAC, AAAAAACC, AAAAACCC, ... ,ACCCCCCC, for which the number of possible indels is reduced from 8 to 6 because 2 indels are superseded by point mutations. For example, consider the sequence AAAAAAAC, which can be aligned to the sequences AAAAAACC and AAAAAAAA using either a point mutation or an indel. The remaining five sequences are ACACTGTG, AGAGTCTC, ATATTATA, CTCTGAGA, GTGTCACA. These sequences also have the number of indels reduced from 8 to 6, but for a more complicated reason. As an example, consider the sequence ACACTGTG, which is separated by a single point mutation from ACACAGTG. The reverse complement of ACACAGTG is CACTGTGT, which can be aligned to ACACTGTG with an indel, a mutation that is superseded by the point mutation from ACACTGTG to ACACAGTG.

5. There are 1008 sequences with 31 neighbors. These sequences have one indel that is superseded by a point mutation. For example, consider the sequence AAAACTTT. A point mutation C $\rightarrow$ G in the 5th position results in the sequence AAAAGTTT, whose reverse complement AAACTTTT can be aligned to AAAACTTT via an indel. This indel is therefore not included in the neighborhood of AAAACTTT, reducing the number of neighbors to 31.

Supplementary figures

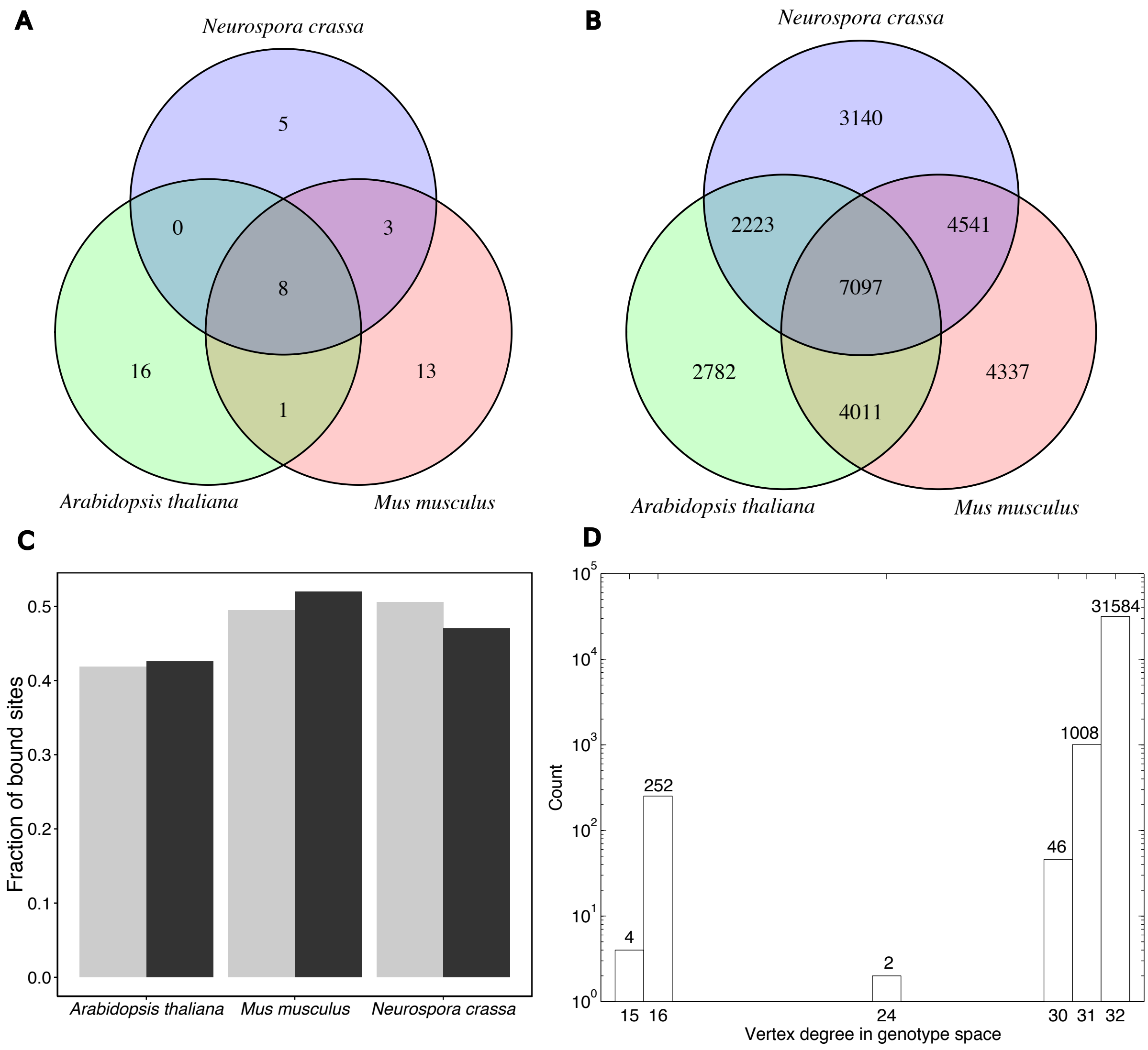

Figure S1. Data. (A) Venn diagram of the DNA-binding domains in the three species analyzed in this study (Table S1). (B) Venn diagram of the binding repertoires of the three species. (C) Amongst all of the sites that bind at least one TF in a given species, the gray bars show the fraction that bind TFs with binding domains that are unique to the species, and the black bars show the fraction that bind TFs with binding domains that are not unique to the species. Bar heights do not sum to one because there are sites bound by both types of TFs. (D) Genotype space is nearly regular. Bar plot of the degree distribution of Ω. Note the logarithmic scale of the y-axis and the counts above each bar.

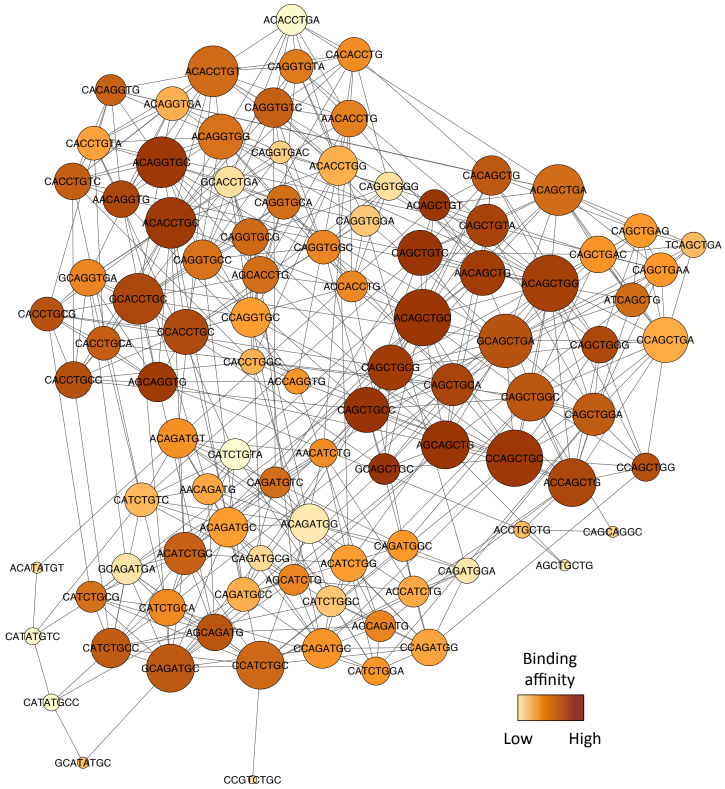

Figure S2. Genotype network of TF binding sites. (A) The dominant genotype network for the murine TF Ascl2. Each vertex corresponds to a DNA sequence that binds Ascl2 (E-score > 0.35). The color of a vertex indicates its binding affinity (darker = higher), while its size corresponds to the number of neighboring sequences (bigger = more). Two sequences are connected by an edge if they are separated by a single small mutation. This mutation may be a point mutation or an indel that shifts the entire binding site by a single position in either the $5'$ or $3'$ direction (Fig. S3).

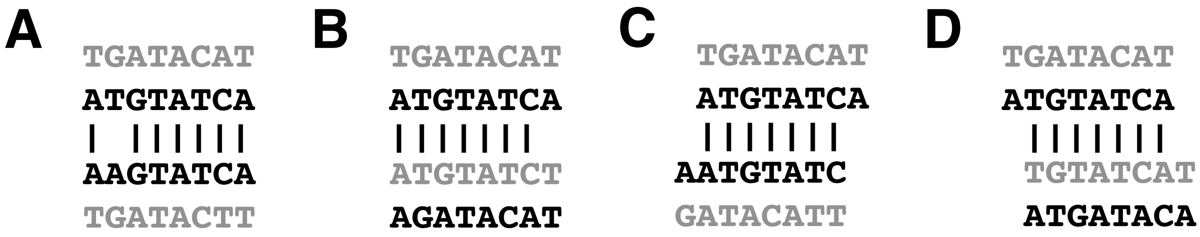

Figure S3. Two forms of mutation. We consider (A,B) point mutations and (C,D) indels that shift an entire, contiguous binding site by a single base. These mutations are illustrated by aligning four different sequences with `ATGTATCA` (top bold-font sequence in each panel). Since every sequence is merged with its reverse complement (gray font), the $4^8 = 65,536$ possible sequences of length eight can be represented by a library of only 32,896 sequences. Sequences that are members of this library are represented in bold font, while their reverse complements are represented in gray font.

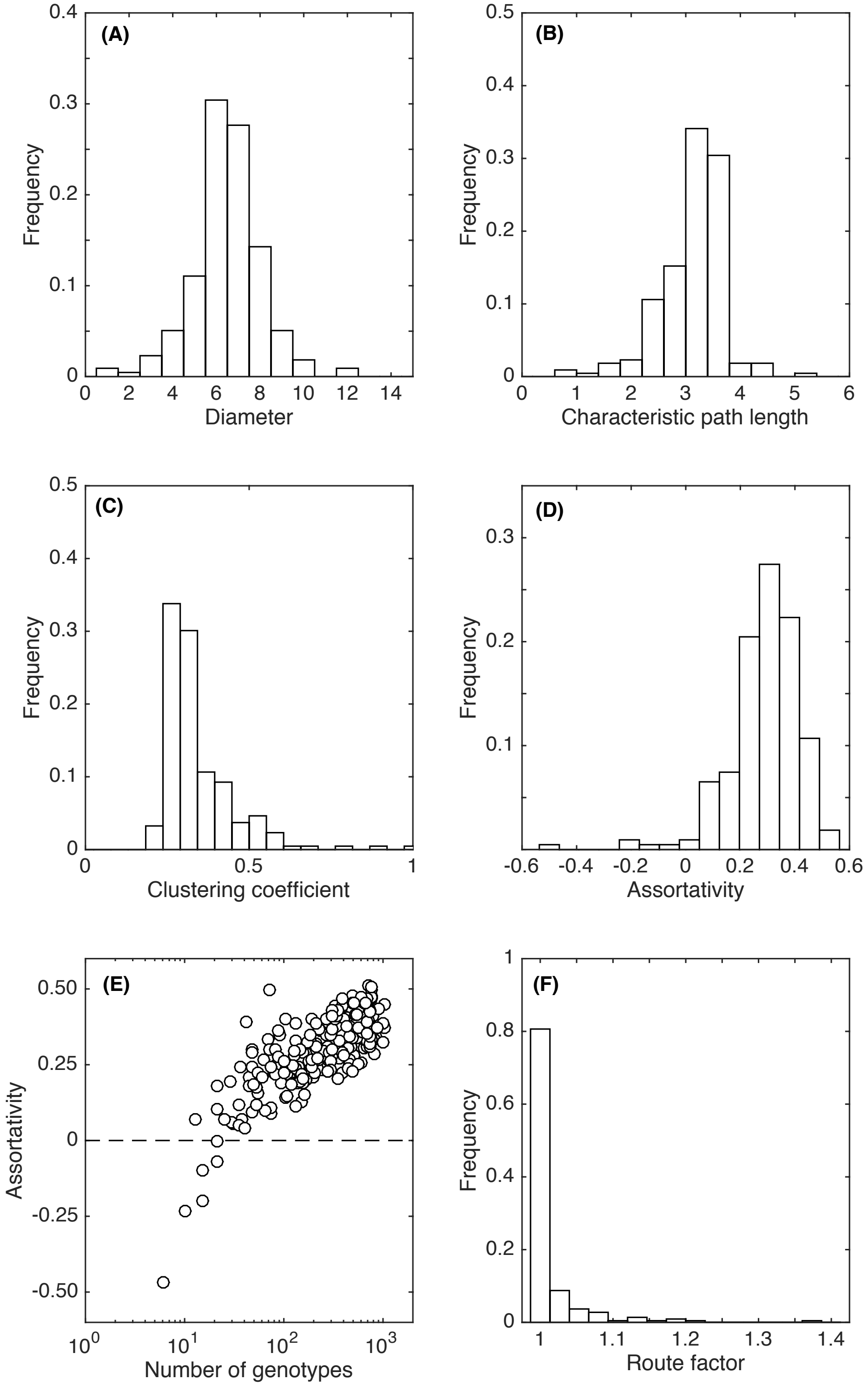

Figure S4. Intranetwork statistics for 217 TFs from *A. thaliana*. The distributions of genotype network (A) diameter, (B) characteristic path length, (C) clustering coefficient, and (D) assortativity. (E) Assortativity (vertical axis) and its relationship to the number of genotypes in the dominant genotype network (horizontal axis). The horizontal dashed line indicates an uncorrelated (non-assortative) mixing pattern. (F) The distribution of the genotype network route factor.

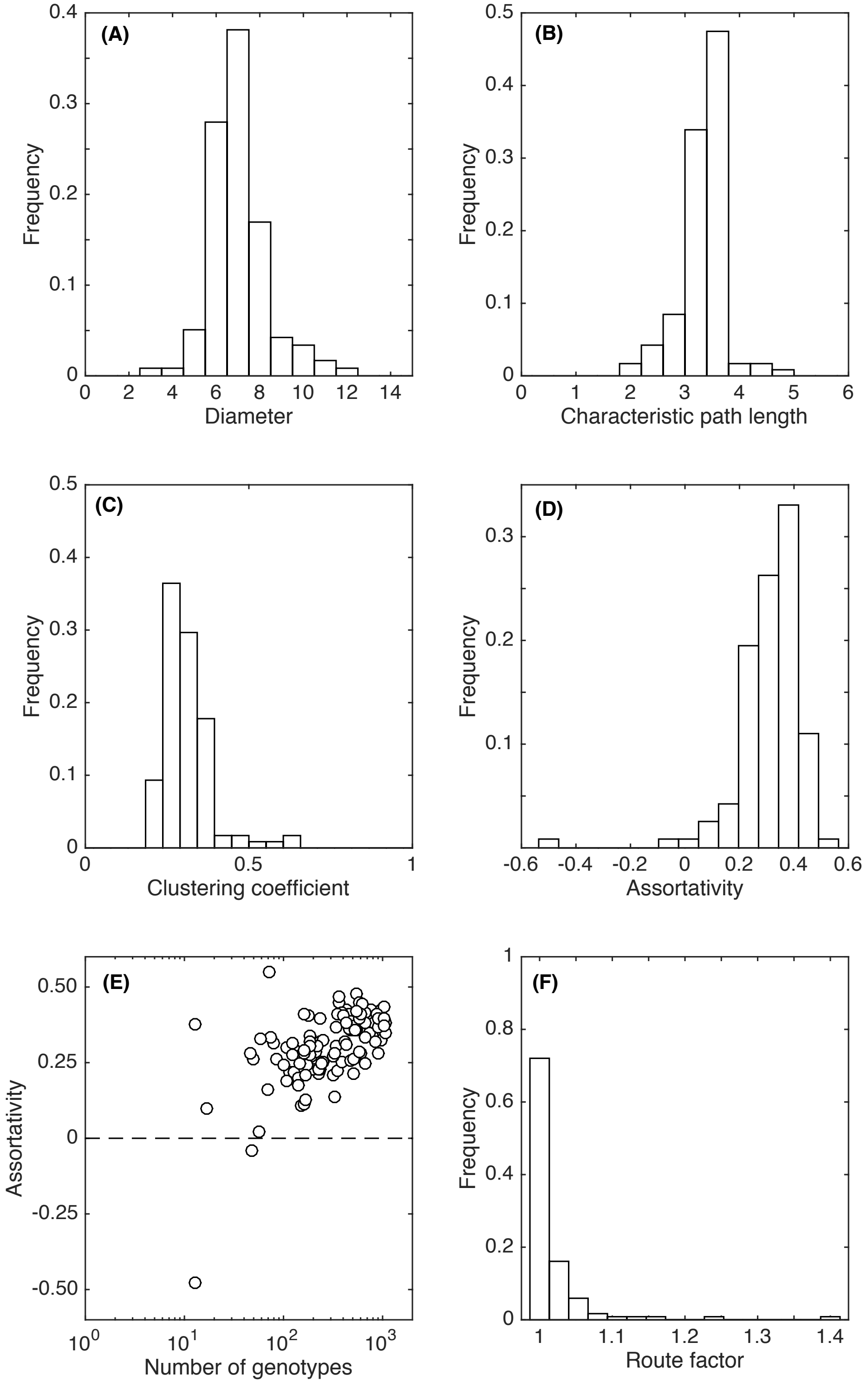

Figure S5. Intranetwork statistics for 118 TFs from *N. crassa*. The distributions of genotype network (A) diameter, (B) characteristic path length, (C) clustering coefficient, and (D) assortativity. (E) Assortativity (horizontal axis) and its relationship to the number of genotypes in the dominant genotype network (vertical axis). The horizontal dashed line indicates an uncorrelated (non-assortative) mixing pattern. (F) The distribution of the genotype network route factor.

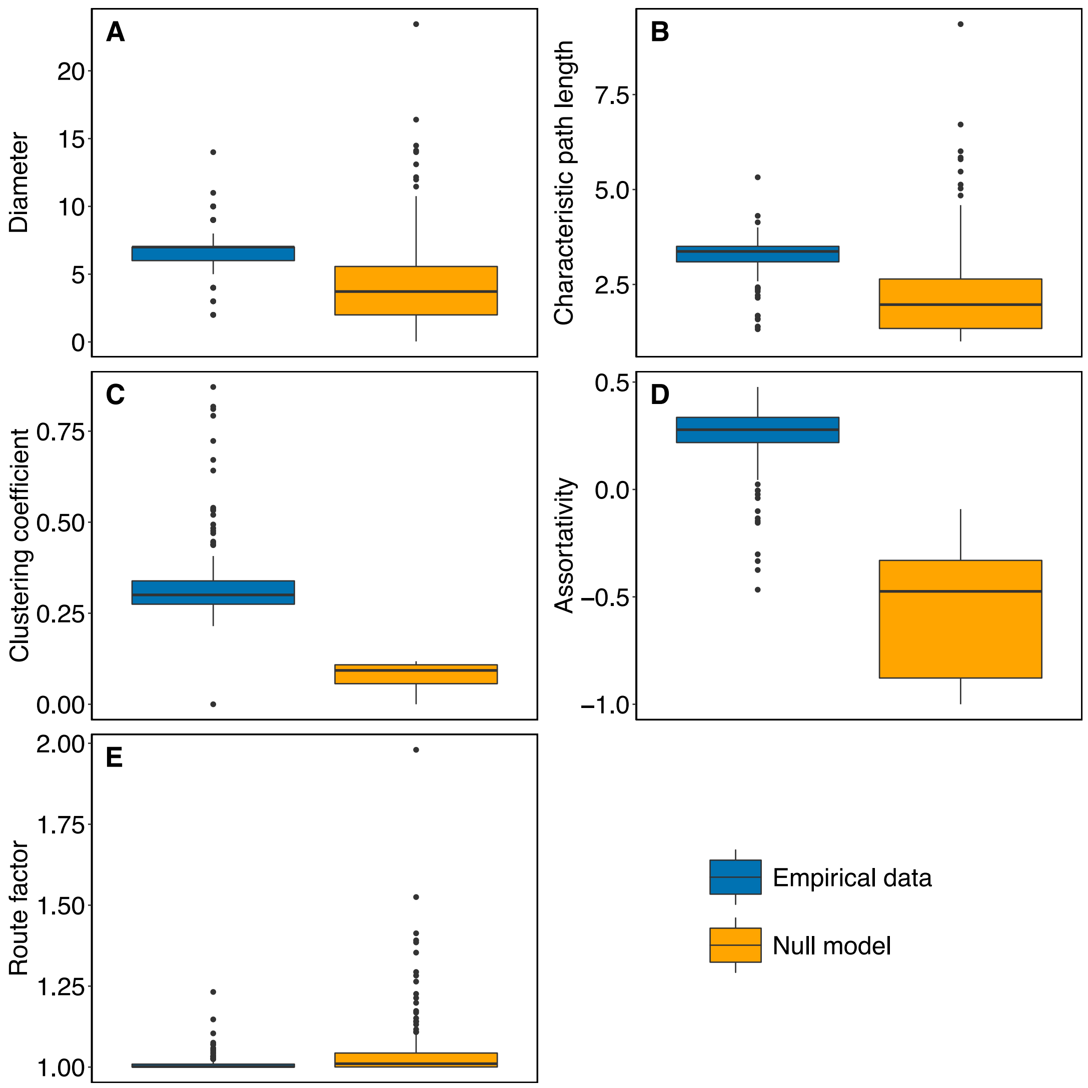

Figure S6. Comparison of intranetwork statistics to those of a null model for 190 TFs from *M. musculus*. The distributions of genotype network (A) diameter, (B) characteristic path length, (C) clustering coefficient, D) assortativity, and (E) route factor.

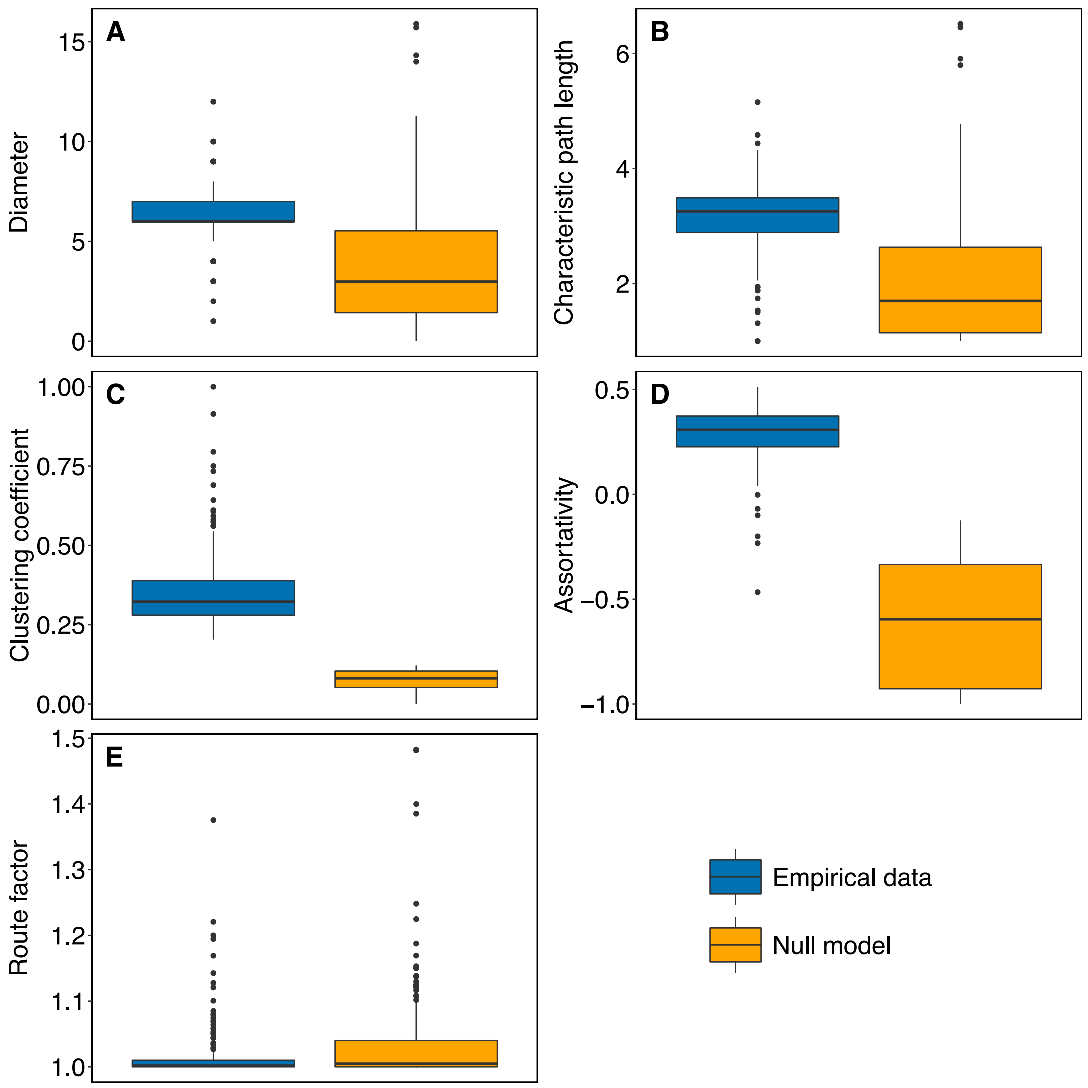

Figure S7. Comparison of intranetwork statistics to those of a null model for 217 TFs from *A. thaliana*. The distributions of genotype network (A) diameter, (B) characteristic path length, (C) clustering coefficient, assortativity, and (E) route factor.

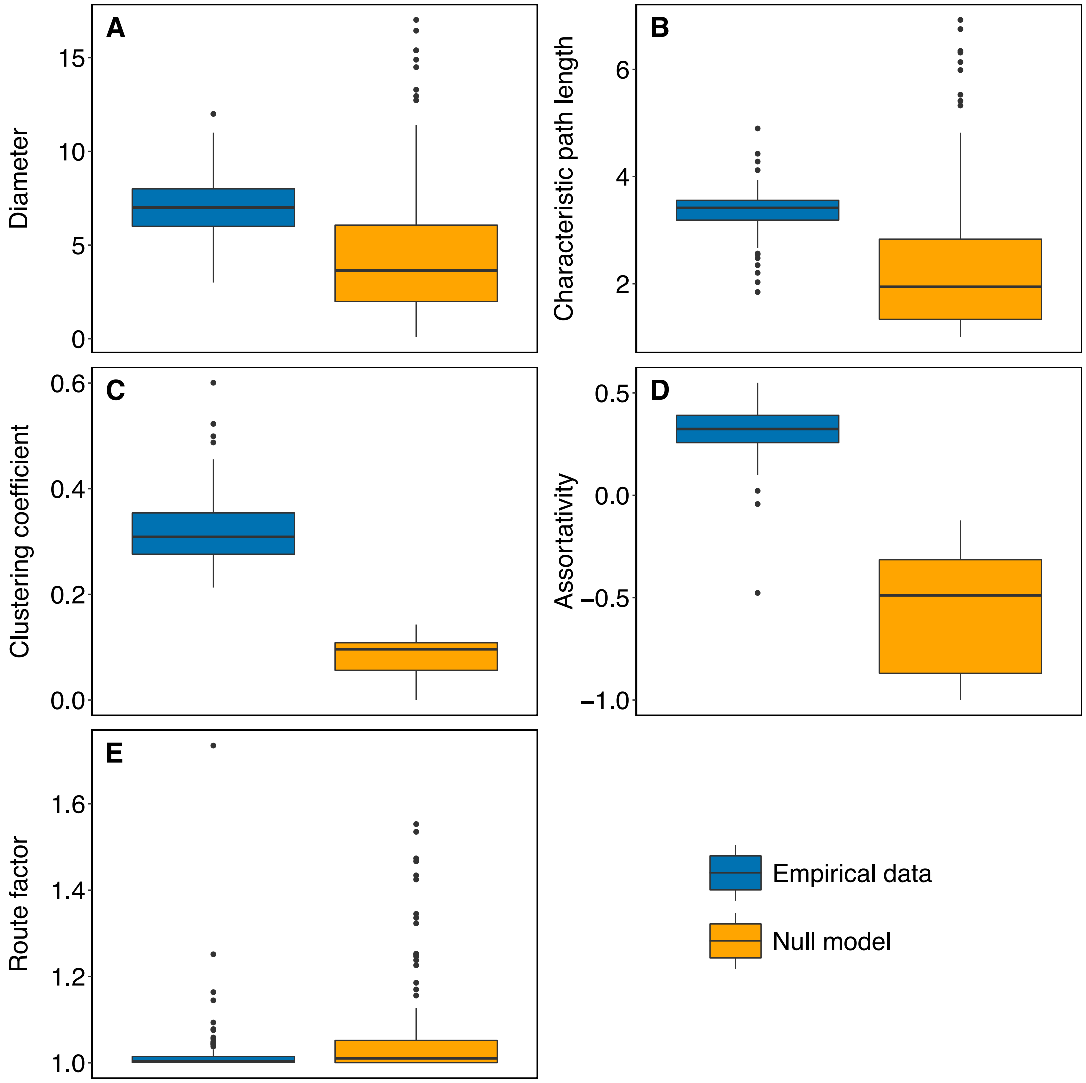

Figure S8. Comparison of intranetwork statistics to those of a null model for 118 TFs from *N. crassa*. The distributions of genotype network (A) diameter, (B) characteristic path length, (C) clustering coefficient, D) assortativity, and (E) route factor.

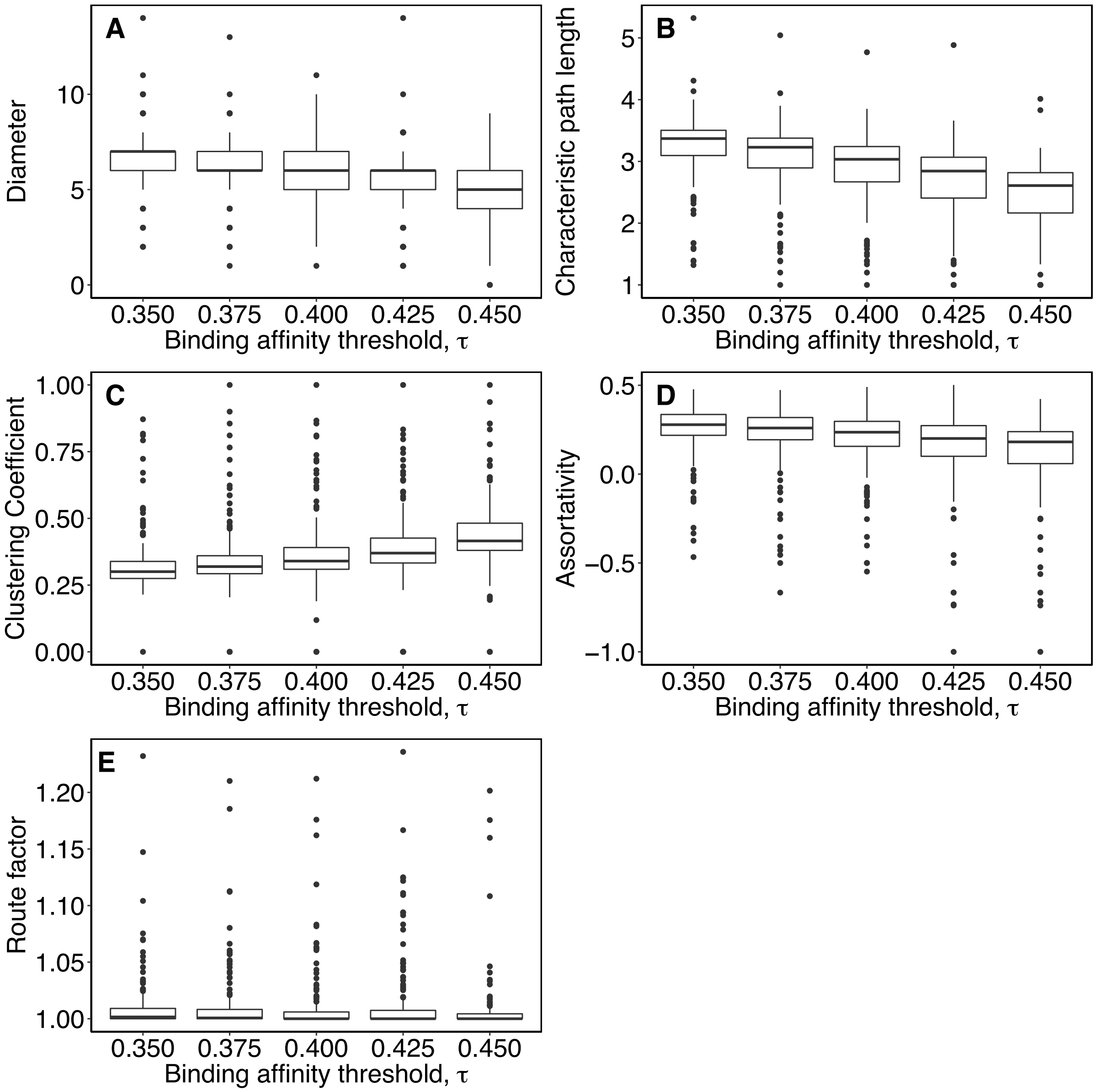

Figure S9. Intranetwork statistics with different binding affinity thresholds for 190 TFs from *M. musculus*. The distributions of genotype network (A) diameter, (B) characteristic path length, (C) clustering coefficient, D) assortativity, and (E) route factor, as a function of the binding affinity threshold. These data represent a sensitivity analysis of the results presented in Fig. 1.

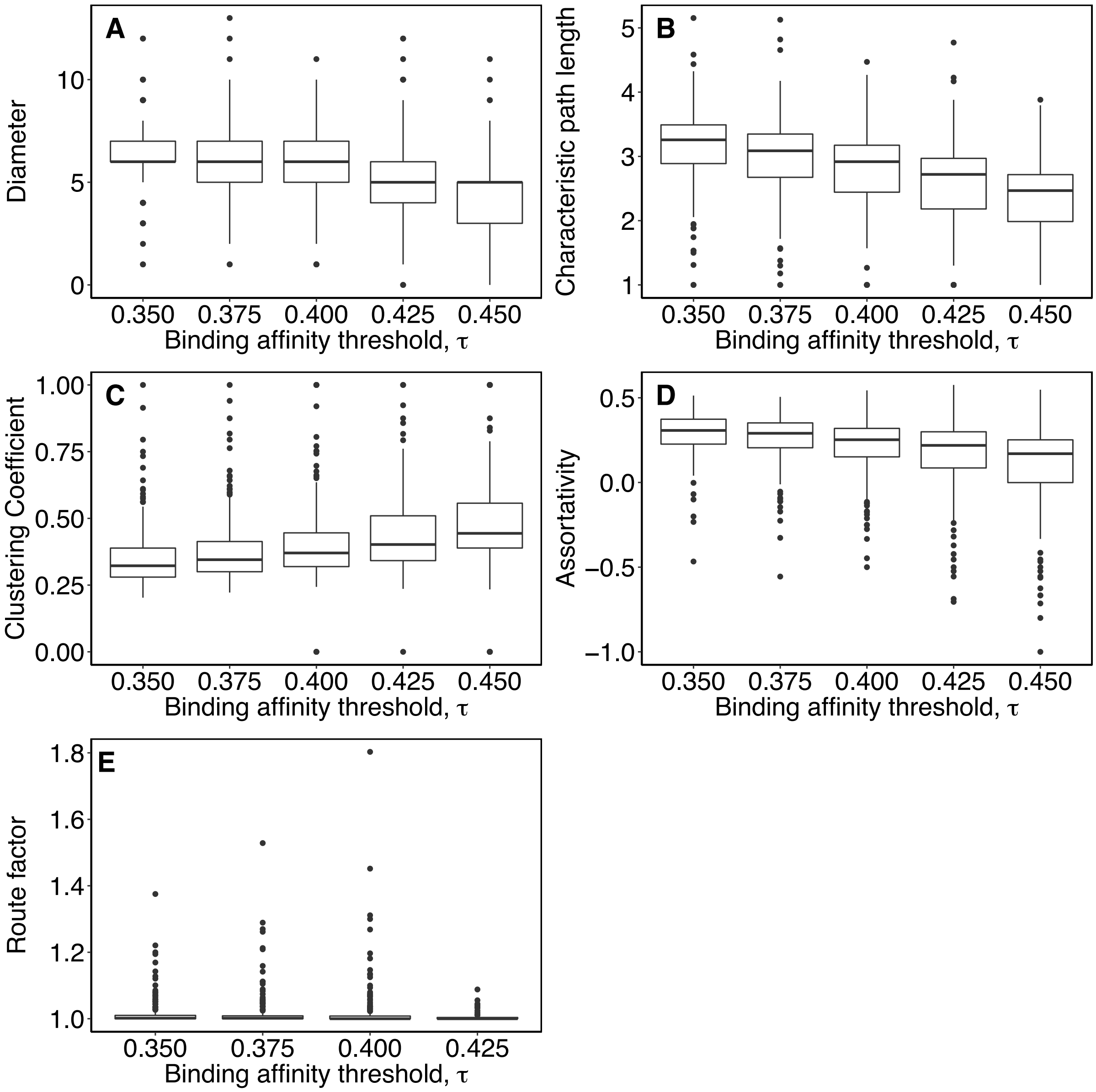

Figure S10. Intranetwork statistics with different binding affinity thresholds for 217 TFs from *A. thaliana*. The distributions of genotype network (A) diameter, (B) characteristic path length, (C) clustering coefficient, D) assortativity, and (E) route factor, as a function of the binding affinity threshold. These data represent a sensitivity analysis of the results presented in Fig. S4.

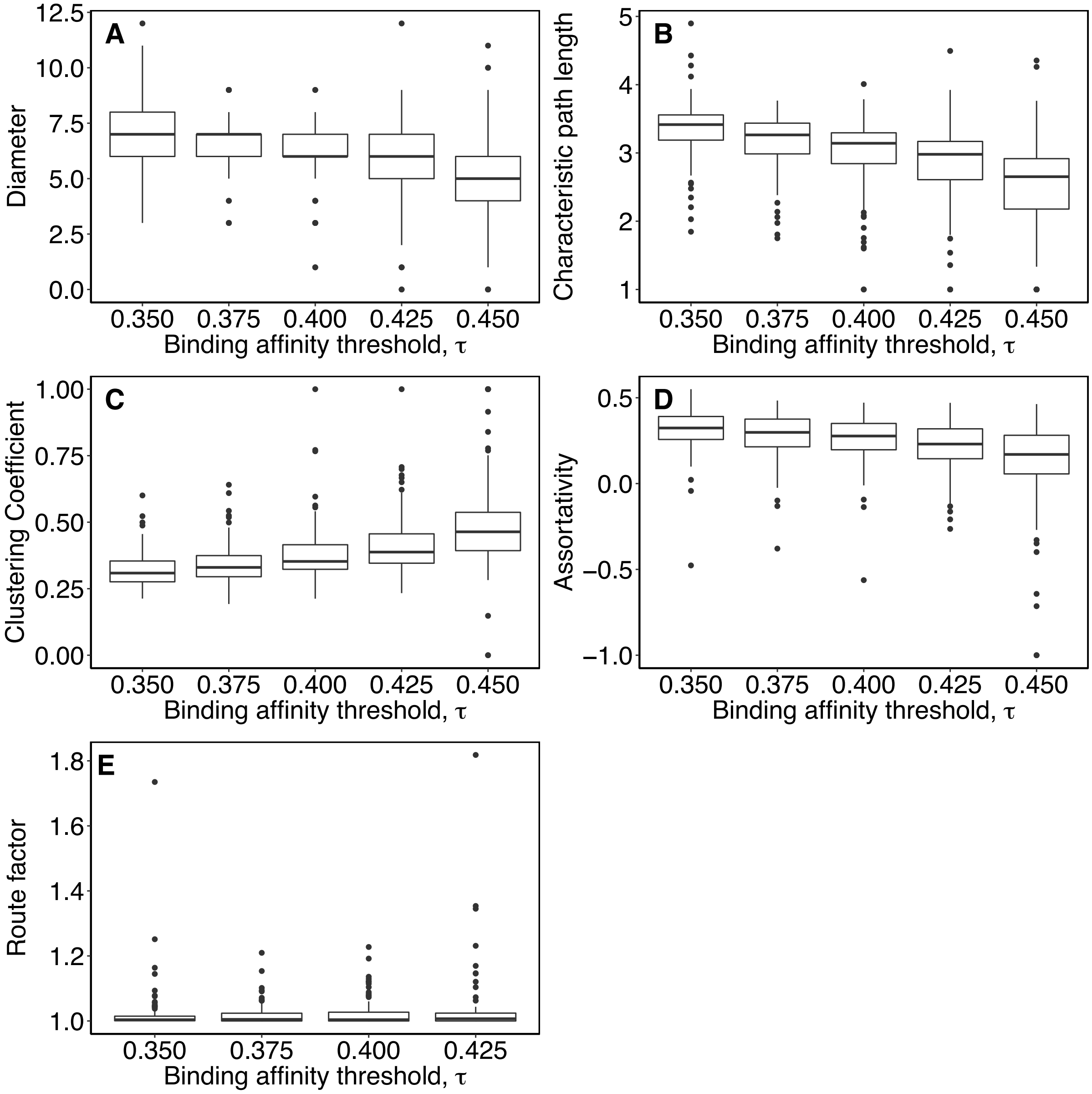

Figure S11. Intranetwork statistics with different binding affinity thresholds for 118 TFs from *N. crassa*. The distributions of genotype network (A) diameter, (B) characteristic path length, (C) clustering coefficient, D) assortativity, and (E) route factor, as a function of the binding affinity threshold. These data represent a sensitivity analysis of the results presented in Fig. S5.

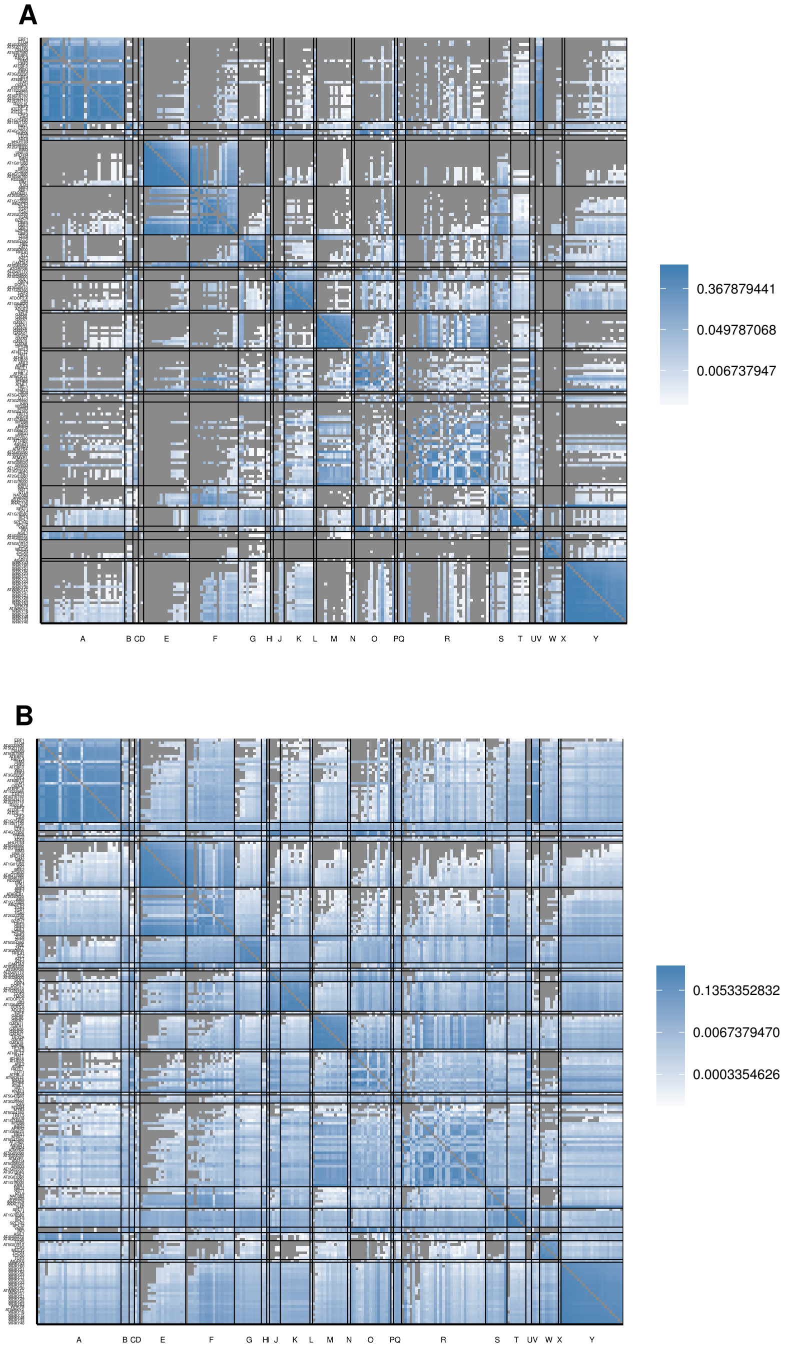

Figure S12. Matrices of internetwork relationships for the genotype networks of TF binding sites from *A. thaliana*. Heatmaps of log10-transformed (A) overlap and (B) ϕ_{qp}, the probability of mutating from the genotype network of phenotype p to the genotype network of phenotype q. The rows and columns are grouped according to binding domain, which are ordered alphabetically on the horizontal axis: A, AP2; B, AP2B3; C, AT hook; D, B3; E, bHLH; F, bZIP; G, C2H2 ZF; H, CG-1; I, CSD; J, CxC; K, Dof; L, E2F; M, GATA; N: GRAS; O, Homeodomain; P, LOB; Q, MADF; R, Myb/SANT; S, NAC/NAM; T, SBP; U, Sox; V, Storekeeper; W, TCP; X, WRC; Y, WRKY. Within the DNA-binding domain groups, the rows and columns are ordered by the size of each TF's dominant genotype network, such that network size increases from top to bottom and from left to right. Labels on the vertical axis indicate the name of the TFs, which can be read on the computer by zooming in. Cells colored in gray indicate either N/A values (on the diagonal) or values equal to zero (off-diagonal).

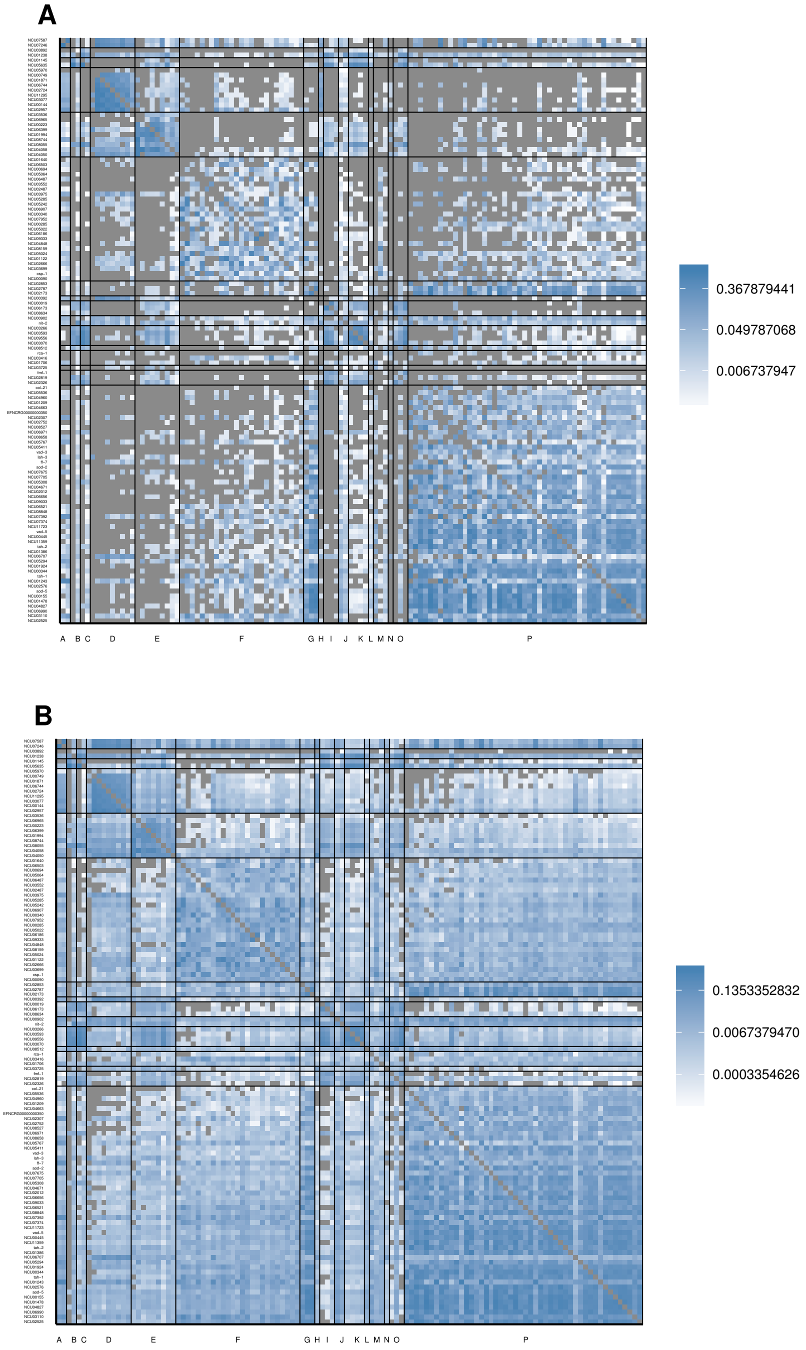

Figure S13. Matrices of internetwork relationships for the genotype networks of TF binding sites from *N. crassa*. Heatmaps of log10-transformed (A) overlap and (B) ϕ_{qp}, the probability of mutating from the genotype network of phenotype p to the genotype network of phenotype q. The rows and columns are grouped according to binding domain, which are ordered alphabetically on the horizontal axis: A, APSES; B, ARID/BRIGHT; C, AT hook; D, bHLH; E, bZIP; F, C2H2 ZF; G, C2H2 ZF + Zinc cluster; H, CENPB; I, Forkhead; J, GATA; K, Homeodomain; L, HSF; M, Myb/SANT; N, Ndt80/PhoG; O, Sox; P, Zinc cluster. Within the DNA-binding domain groups, the rows and columns are ordered by the size of the dominant genotype net=work, such that network size increases from top to bottom and from left to right. Labels on the vertical axis indicate the name of the TFs, which can be read on the computer by zooming in. Cells colored in gray indicate either N/A values (on the diagonal) or values equal to zero (off-diagonal).

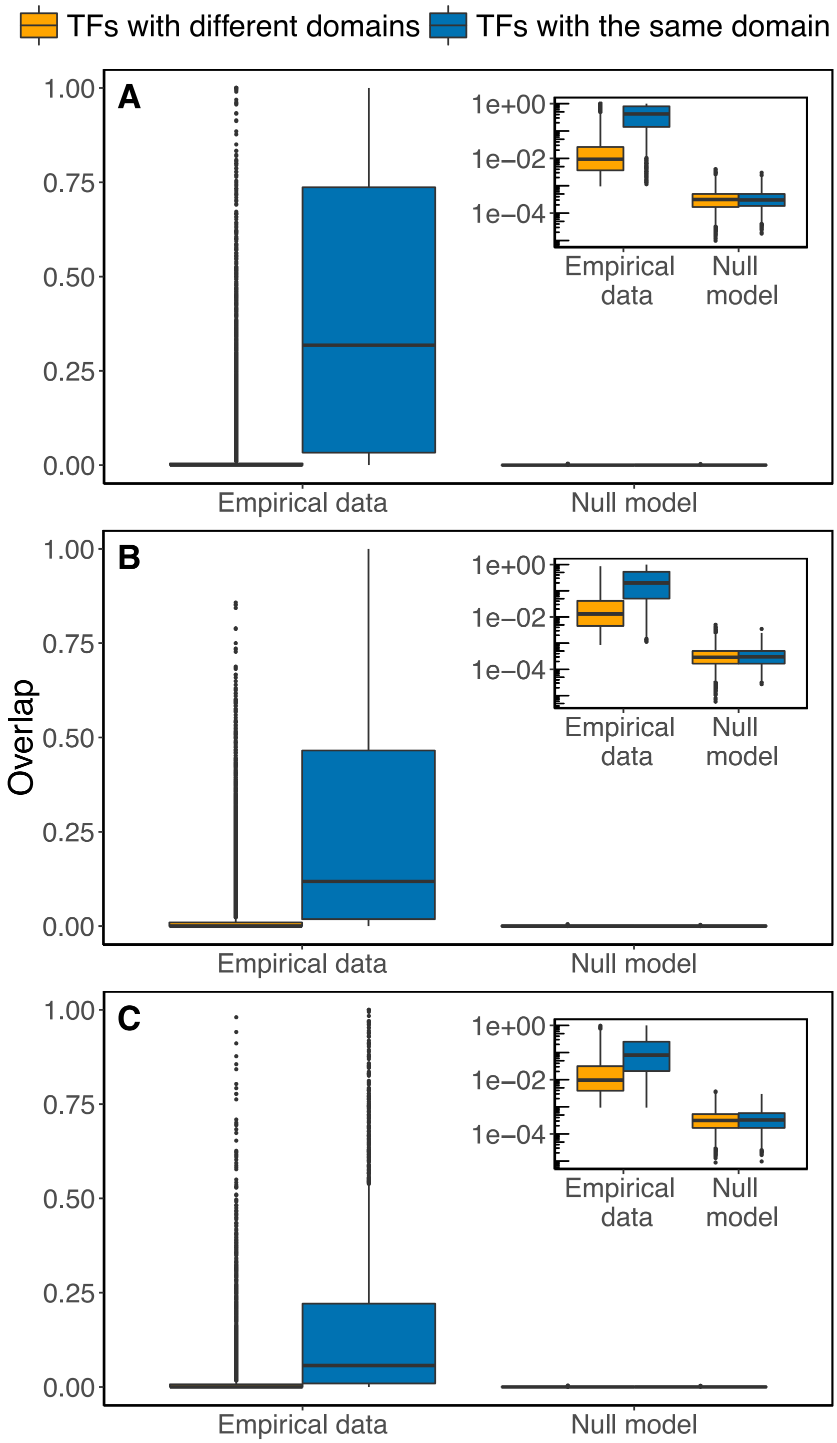

Figure S14. Comparison of overlap in the empirical data to that in the null model. The empirical data is the same as presented in Figs. 3A, S12A and S13A. TFs that belong to the same DNA-binding domain family show higher overlap than TFs belonging to different domain families, but this difference is not observed in the null model. This observation is made in all three species: (A) *A. thaliana*, (B) *M. musculus*, and (C) *N. crassa*. Insets show the same data as the main panels, but with a logarithmically-scaled y-axis.

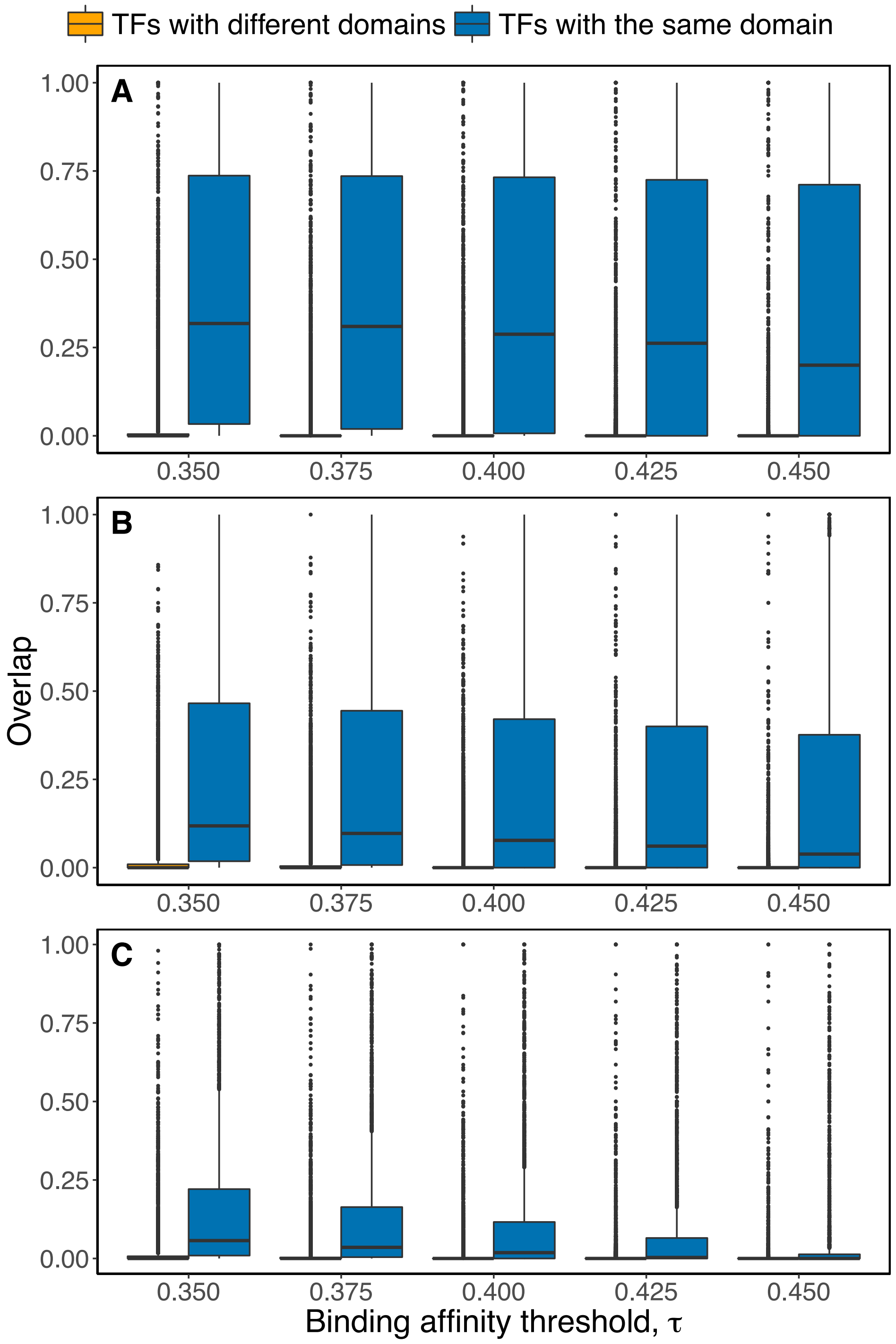

Figure S15. Genotype network overlap in relation to the binding affinity threshold. These data represent a sensitivity analysis of the results presented in Figs. 3A, S12A and S13A. TFs that belong to the same DNA-binding domain family show higher overlap than TFs belonging to different domain families. This observation does not change with binding affinity threshold in (A) *A. thaliana*, (B) *M. musculus*, and (C) *N. crassa*.

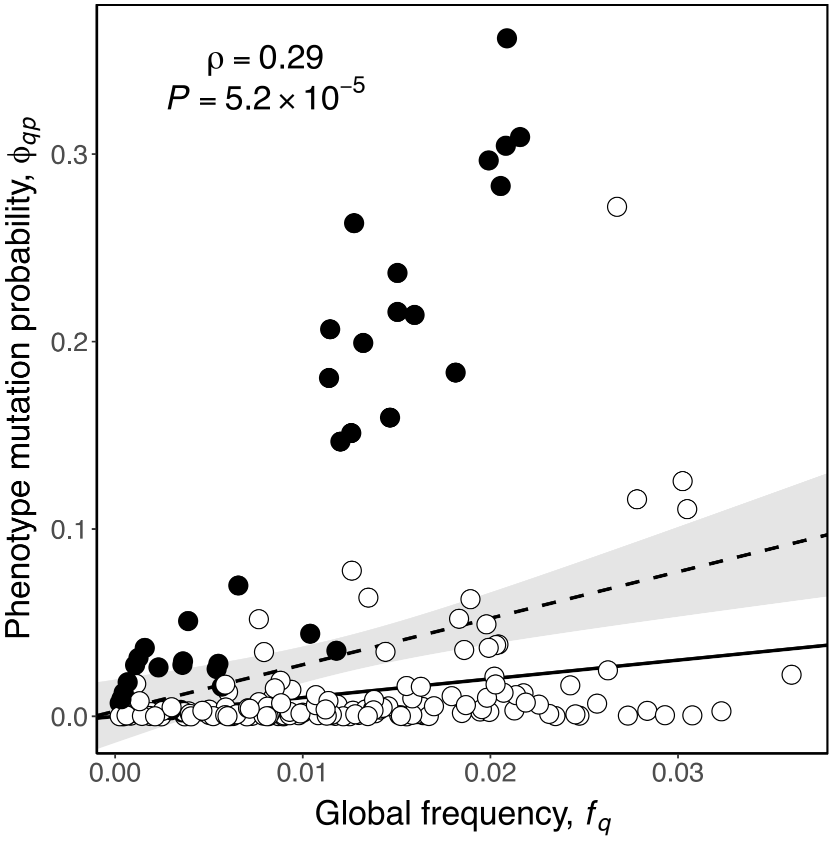

Figure S16. A simple null model does not provide a reasonable approximation to ϕ_{qp}. The probability ϕ_{qp} that a mutation to a genotype with phenotype p creates a genotype with phenotype q is shown in relation to the frequency f_q of phenotype q. The black line shows the null expectation that $\phi_{qp} = f_q$ (Greenbury et al. 2016). The dashed line shows the fitted linear regression to the data ($R^2 = 0.082$) and the shaded grey band around the line denotes 95% confidence intervals. The figure also shows the Spearman's correlation (ρ) between phenotype mutation probability and phenotype frequency, and its associated P value. Each circle represents the ϕ_{qp} of a different phenotype p, where phenotype q is always that of the murine TF Hes7. Black circles correspond to TFs with bHLH binding domain, and white circles correspond to TFs with a different binding domain. Half circles at the bottom of the panel denote pairs of phenotypes with $\phi_{qp} = 0$.

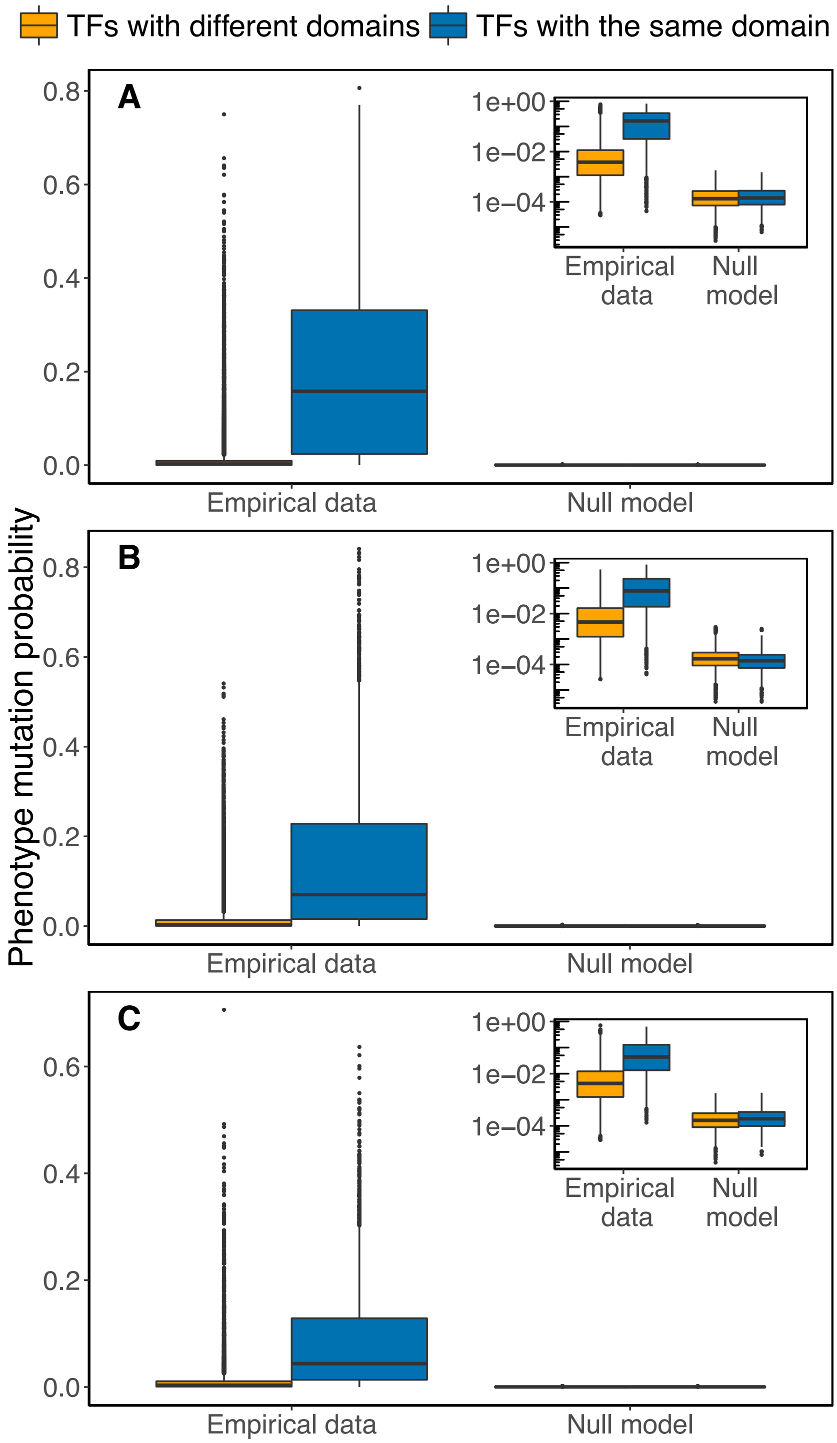

Figure S17. Comparison of phenotype mutation probabilities in the empirical data to those of a null model. The empirical data is the same as presented in Fig. 3B, S12B and S13B. TFs that belong to the same DNA-binding domain family show higher phenotype mutation probabilities than TFs belonging to different domain families, but this difference is not observed in the null model. This observation is made in all three species: (A) *A. thaliana*, (B) *M. musculus*, and (C) *N. crassa*. Insets show the same data as the main panels, but with a logarithmically-scaled y-axis.

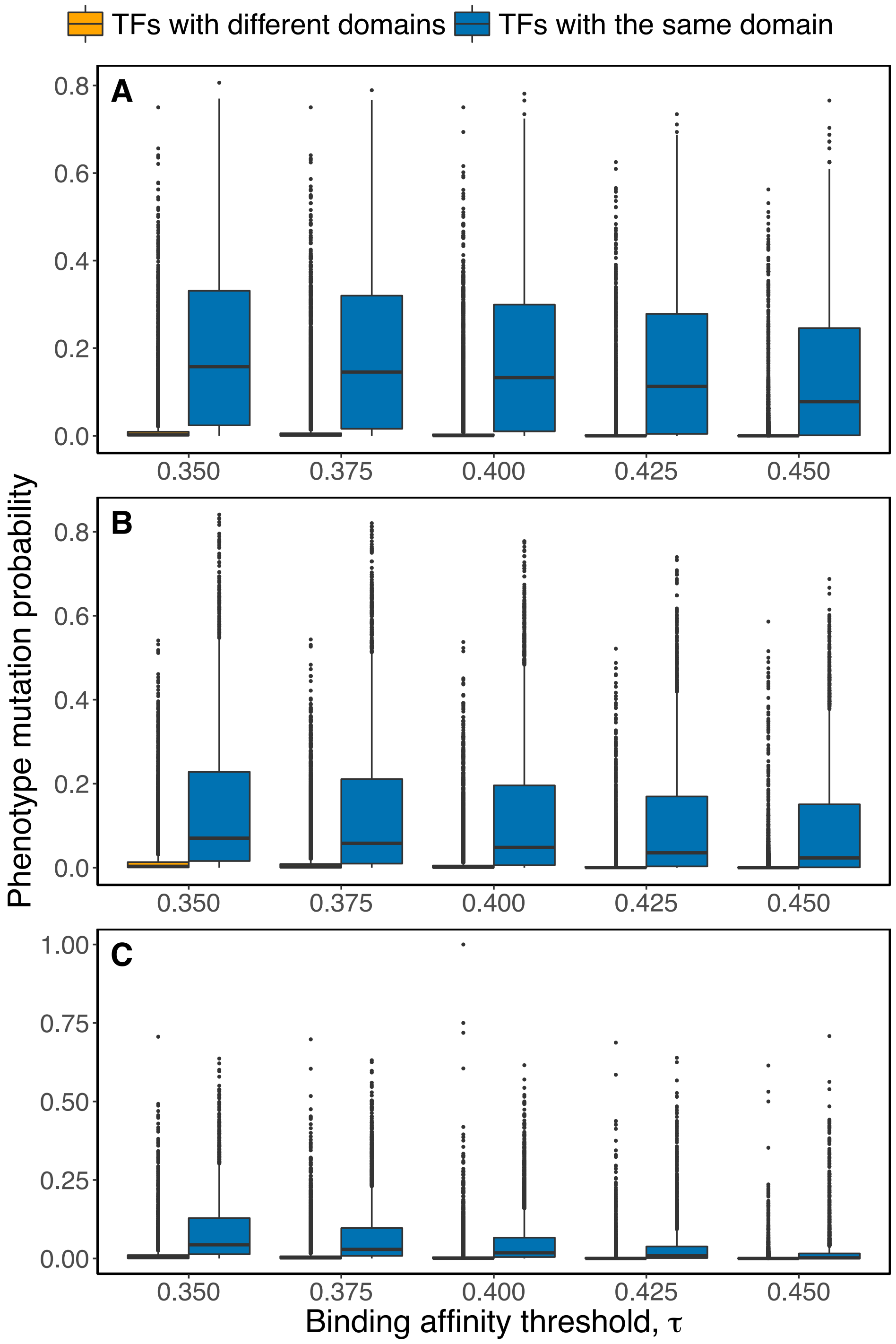

Figure S18. Phenotype mutation probabilities in relation to binding affinity threshold. These data represent a sensitivity analysis of the results presented in Fig. 3B, S12B and S13B. TFs that belong to the same DNA-binding domain family show higher phenotype mutation probability than TFs belonging to different domain families. This observation does not change with binding affinity threshold in (A) *A. thaliana*, (B) *M. musculus*, and (C) *N. crassa*.

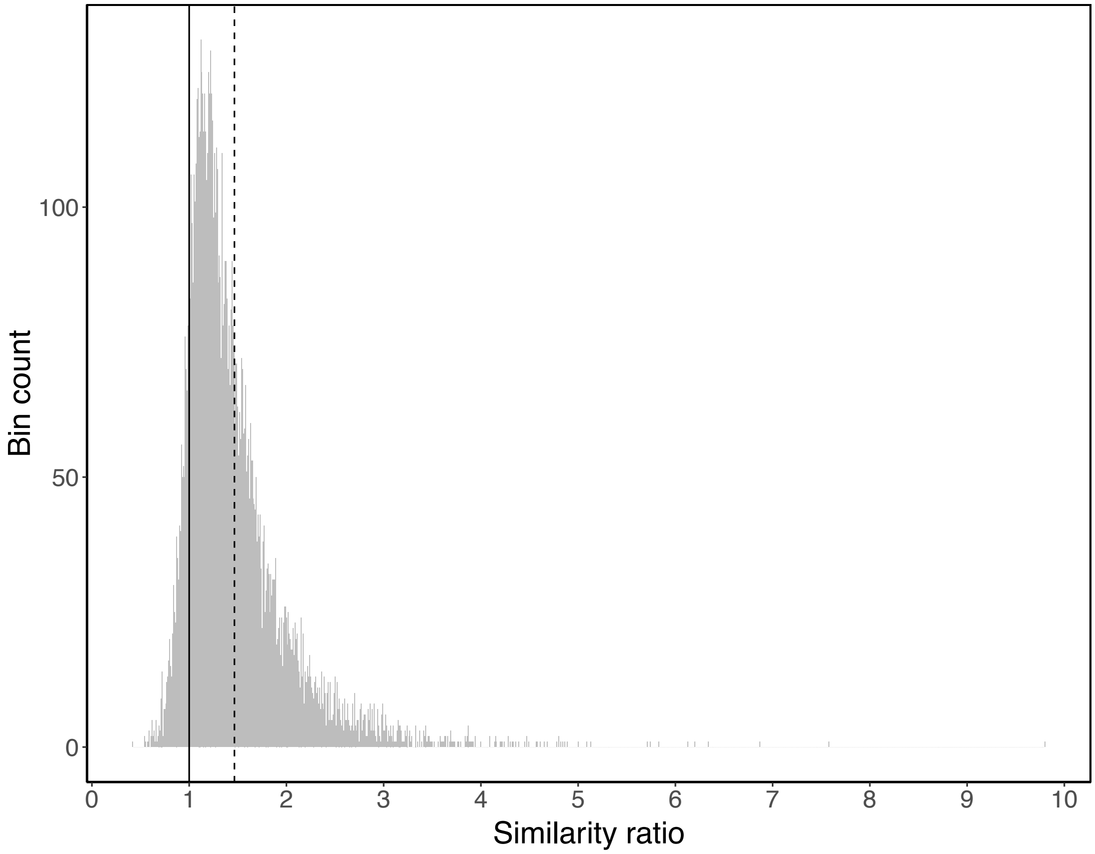

Figure S19. In *M. musculus*, the phenotypes found in the mutational neighborhoods of neutral neighbors are more similar than those of neutral pairs that are not neighbors. The distribution of the similarity ratio (Eq. 9) of the phenotype probability distributions (Eq. 6) is shown for neutral neighbors (n_1 and n_2) and neutral pairs that are not neighbors (n_1 and n_3). For this analysis, we considered all 9,207 pairs of neutral neighbors in the genotype network for Sp110, and sampled the same number of neutral pairs that are not neighbors. The mutational neighborhoods of n_1 and n_2 are more similar than those of n_1 and n_3, because the mean of the ratio (vertical dashed line) is larger than unity (vertical solid line). The standard error of this mean (0.006) is minute compared to the difference between the mean and unity (0.465).

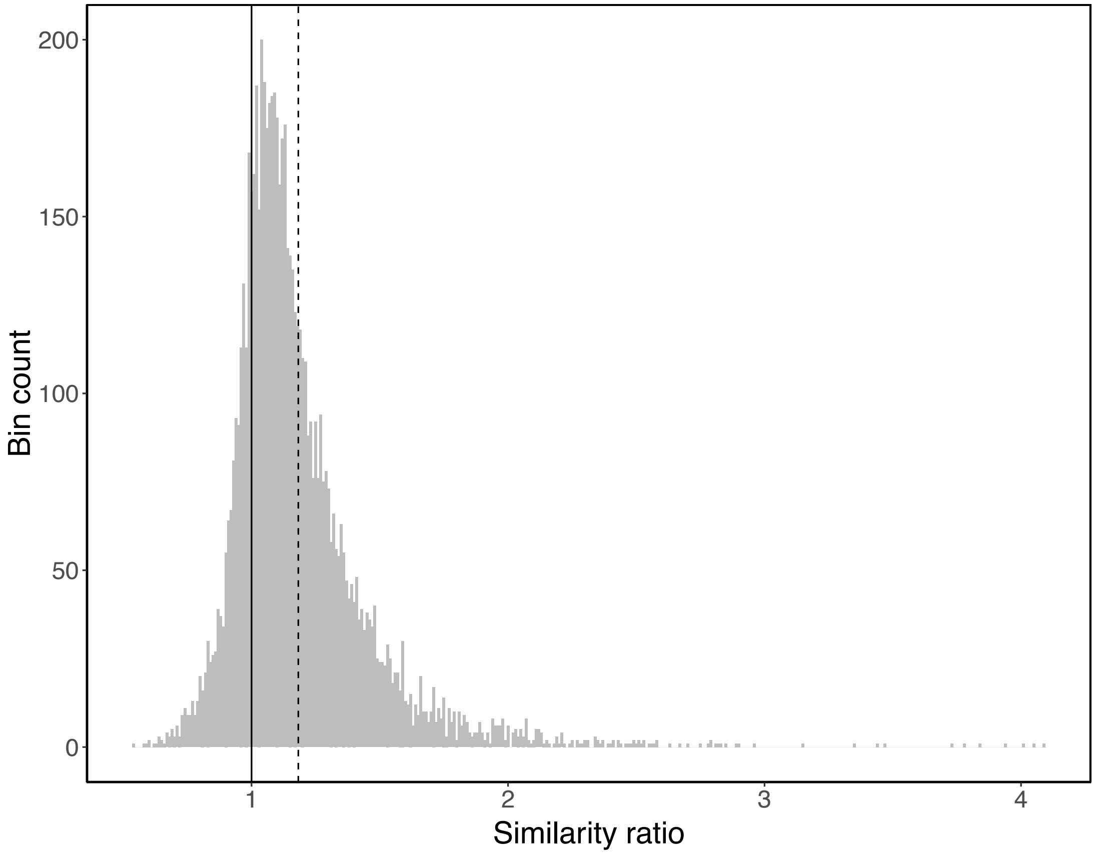

Figure S20. In *A. thaliana*, the phenotypes in the mutational neighborhoods of neutral neighbors are more similar than those of neutral pairs that are not neighbors. The distribution of the similarity ratio (Eq. 9) of the phenotype probability distributions (Eq. 6) is shown for neutral neighbors (n_1 and n_2) and for neutral pairs that are not neighbors (n_1 and n_3). For this analysis, we considered all 7,098 pairs of neutral neighbors in the genotype network for AZF2, and sampled the same number of neutral pairs that are not neighbors. The mutational neighborhoods of n_1 and n_2 are more similar than those of n_1 and n_3, because the mean of the ratio (vertical dashed line) is larger than unity (vertical solid line). The standard error of this mean (0.003) is minute compared to the difference between the mean and unity (0.182).

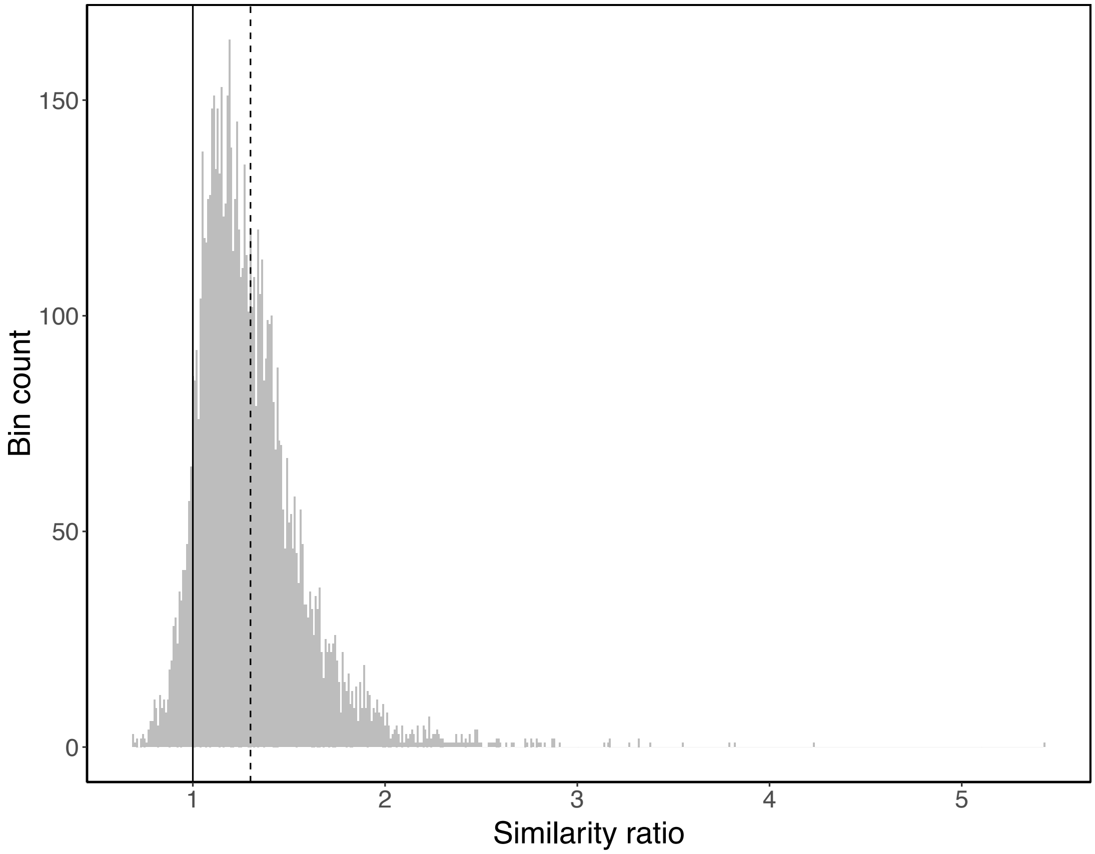

Figure S21. In *N. crassa*, the phenotypes in the mutational neighborhoods of neutral neighbors are more similar than those of neutral pairs that are not neighbors. The distribution of the similarity ratio (Eq. 9) of the phenotype probability distributions (Eq. 6) is shown for neutral neighbors (n_1 and n_2) and for neutral pairs that are not neighbors (n_1 and n_3). For this analysis, we considered all 7,379 pairs of neutral neighbors in the genotype network for NCU02525, and sampled the same number of neutral pairs that are not neighbors. The mutational neighborhoods of n_1 and n_2 are more similar than those of n_1 and n_3, because the mean of the ratio (vertical dashed line) is larger than unity (vertical solid line). The standard error of this mean (0.003) is minute compared to the difference between the mean and unity (0.3).

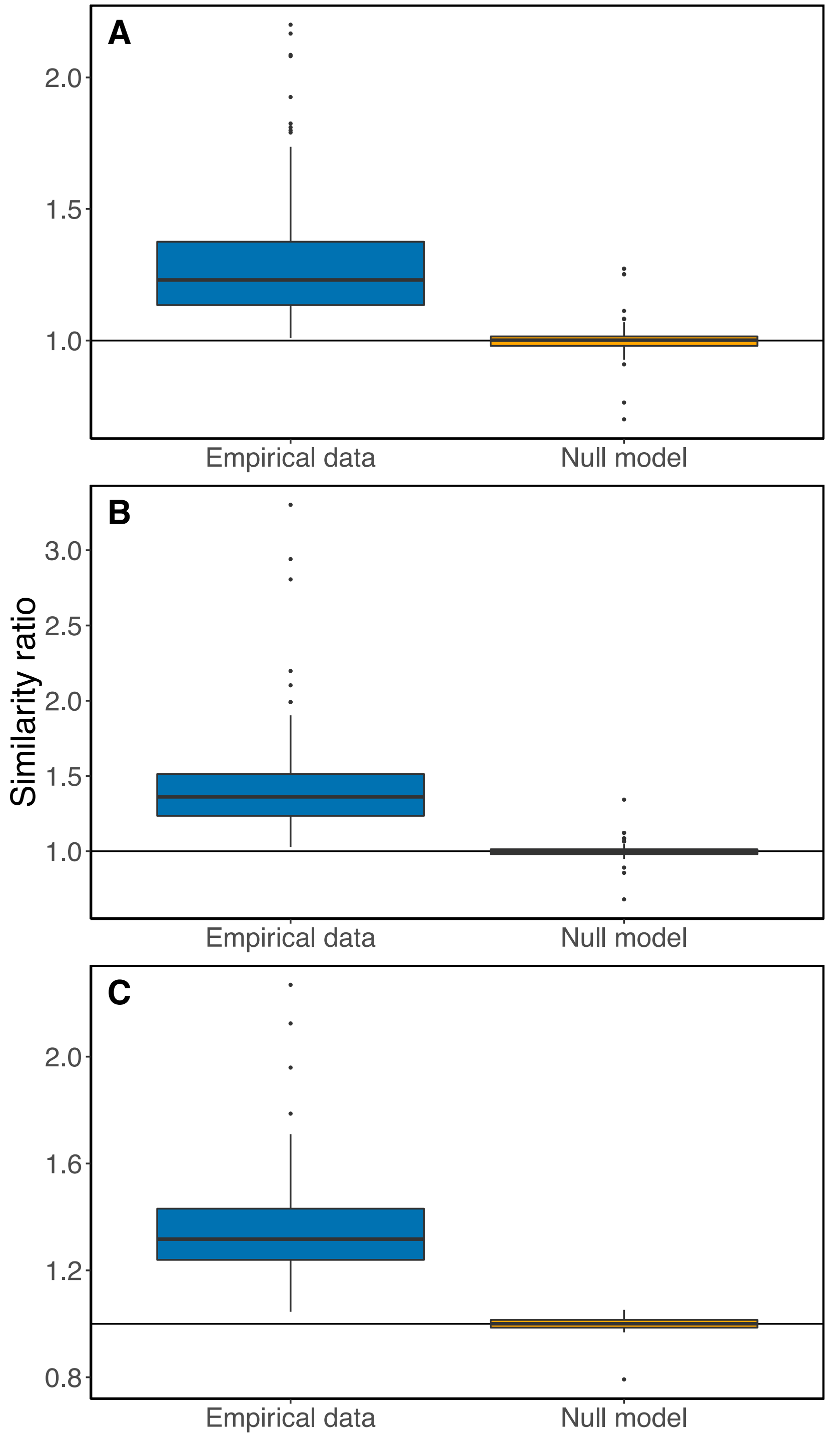

Figure S22. Comparison of similarity ratios in the empirical data to those of a null model. The phenotypes in the mutational neighborhoods of neutral neighbors are more similar than those of neutral pairs that are not neighbors: The median similarity ratio is larger than unity (horizontal solid line). This does not happen with the null model in any of the three species: (A) *A. thaliana*, (B) *M. musculus*, or (C) *N. crassa*. The empirical data is the same as presented in Figs. S19A, S20A, and S21A.

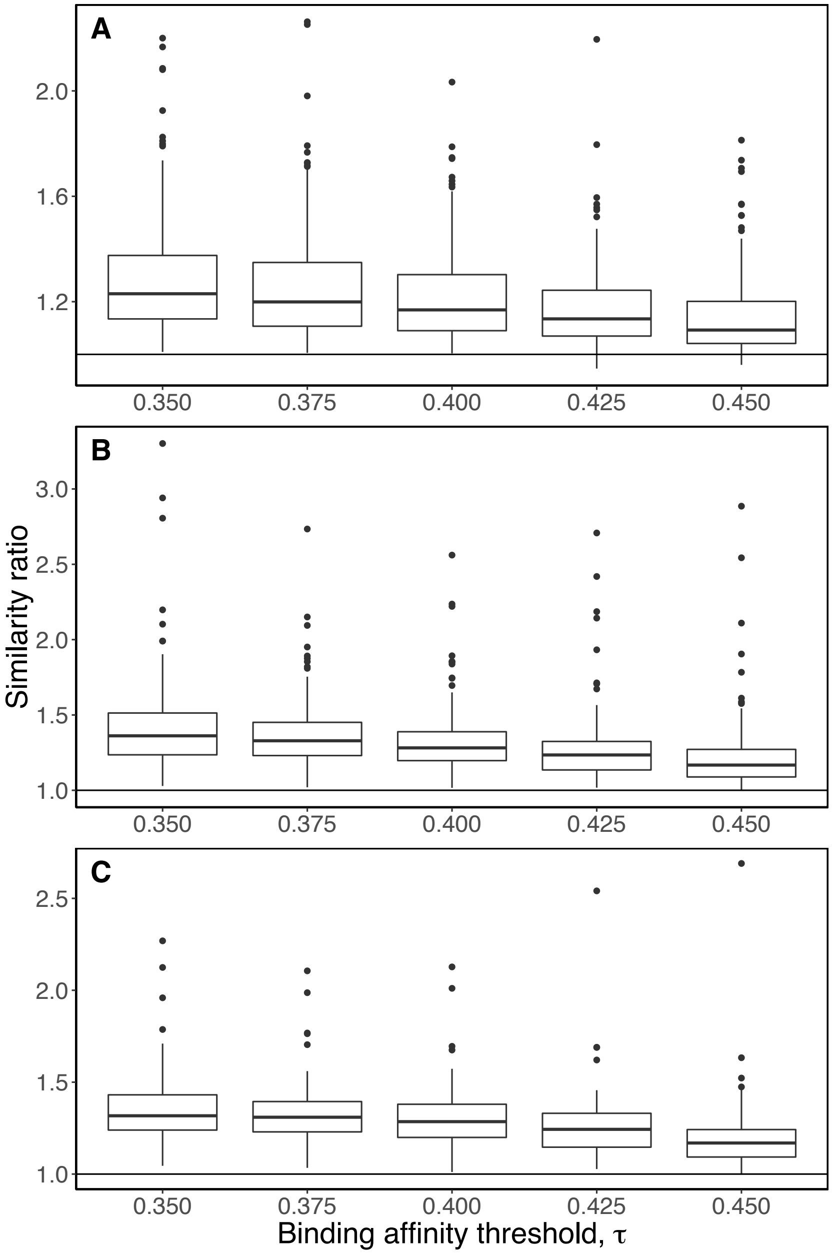

Figure S23. Similarity ratios with different binding affinity thresholds. The phenotypes in the mutational neighborhoods of neutral neighbors are more similar than those of neutral pairs that are not neighbors: The median similarity ratio is larger than unity (horizontal solid line) for all binding affinity thresholds. This observation does not change with binding affinity threshold in (A) *A. thaliana*, (B) *M. musculus*, and (C) *N. crassa*. These data represent a sensitivity analysis of the results presented in Figs. S19A, S20A, and S21A.

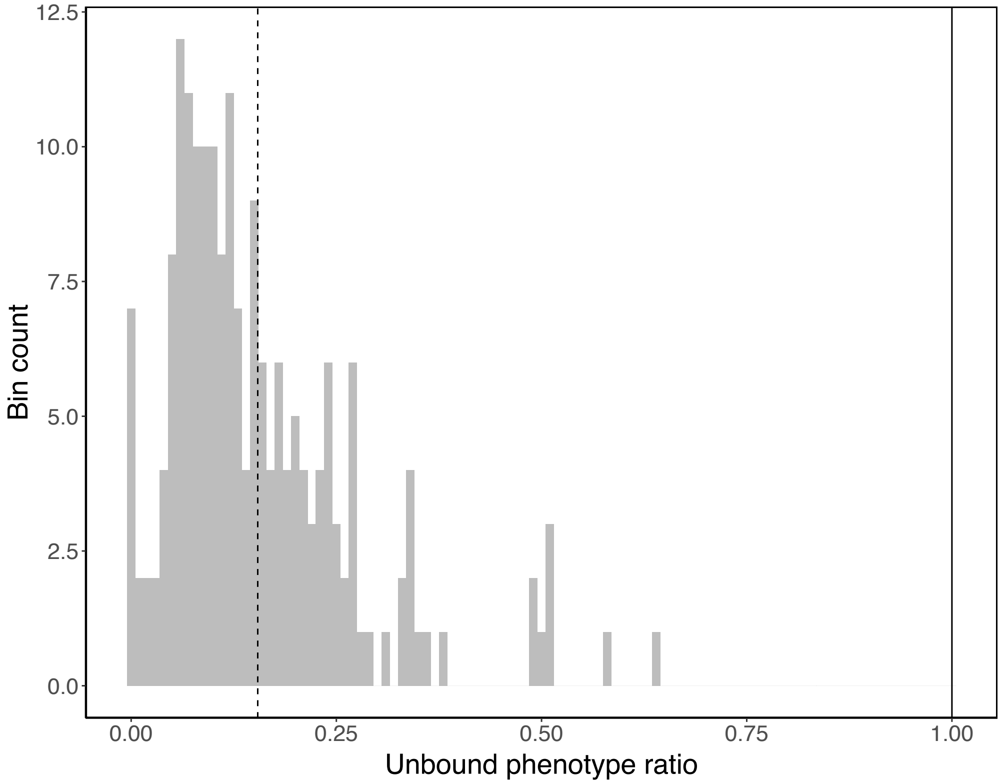

Figure S24. In *M. musculus*, unbound sites are underrepresented in the neighborhoods of bound sites. The distribution of the ratio $\phi_{\text{unbound},p}/f_{\text{unbound}}$, which is the probability of mutating from a sequence bound by TF p to an unbound sequence, divided by the null expectation of the frequency of unbound DNA sequences. The distribution is clearly skewed to values smaller than one, as shown by the distance of the distribution (vertical dashed line) to unity (vertical solid line).

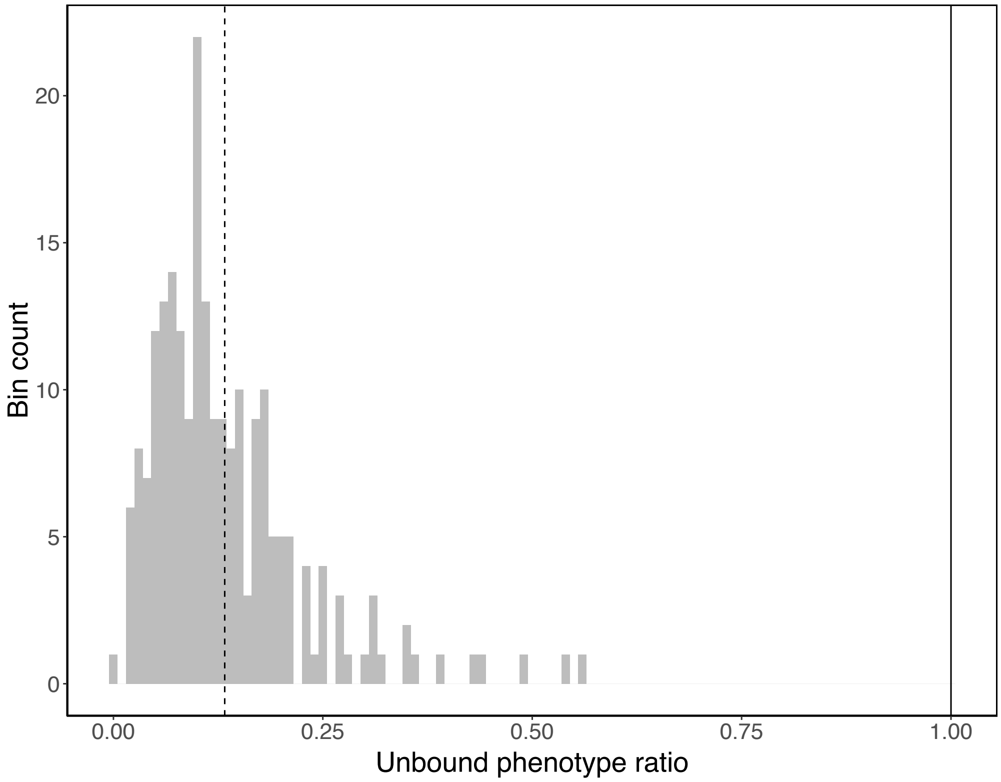

Figure S25. In *A. thaliana*, unbound sites are underrepresented in the neighborhoods of bound sites. The distribution of the ratio $\phi_{\mathrm{unbound},p}/f_{\mathrm{unbound}}$, which is the probability of mutating from a sequence bound by TF p to an unbound sequence, divided by the null expectation of the frequency of unbound DNA sequences. The distribution is clearly skewed to values smaller than one, as shown by the distance of the distribution (vertical dashed line) to unity (vertical solid line).

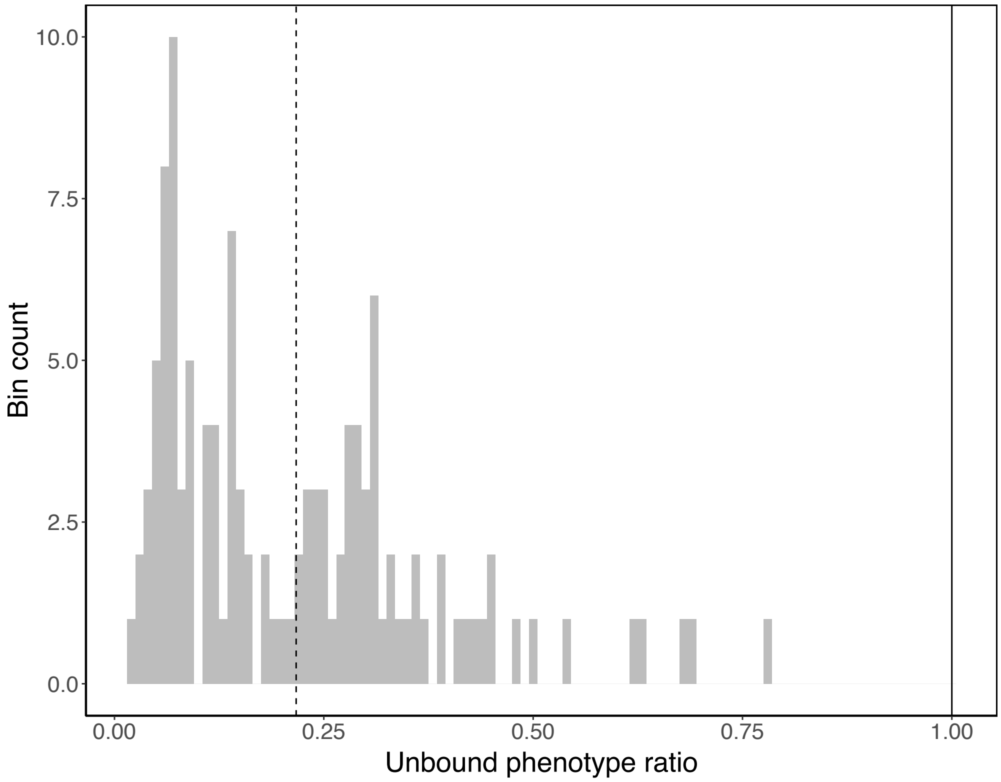

Figure S26. In *N. crassa*, unbound sites are underrepresented in the neighborhoods of bound sites. The distribution of the ratio $\phi_{\text{unbound},p}/f_{\text{unbound}}$, which is the probability of mutating from a sequence bound by TF p to an unbound sequence, divided by the null expectation of the frequency of unbound DNA sequences. The distribution is clearly skewed to values smaller than one, as shown by the distance of the distribution (vertical dashed line) to unity (vertical solid line).

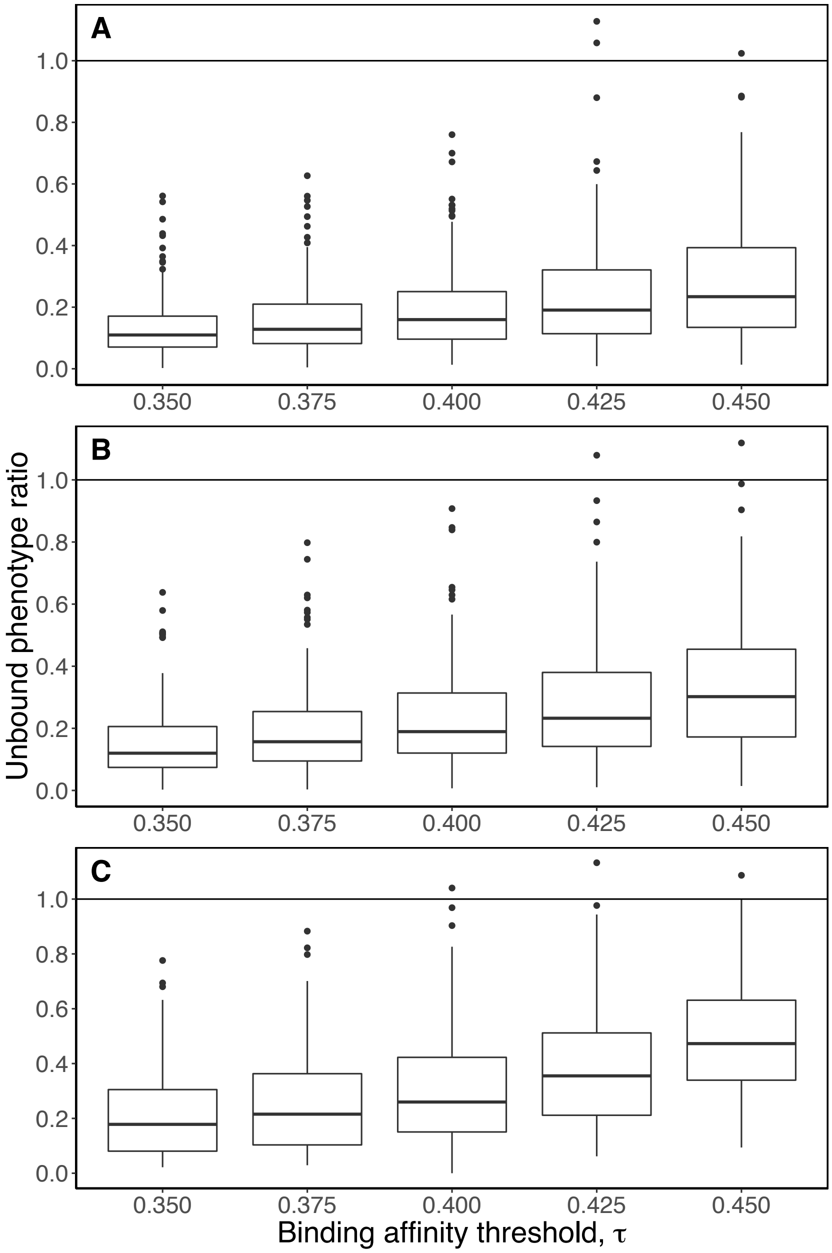

Figure S27. Unbound sites are underrepresented in the neighborhoods of bound sites for all binding affinity thresholds. The distribution of the ratio $\phi_{\mathrm{unbound},p}/f_{\mathrm{unbound}}$, which is the probability of mutating from a sequence bound by TF p to an unbound sequence, divided by the null expectation of the frequency of unbound DNA sequences. The distribution is skewed toward values smaller than one, as shown by the distance of the median of the distribution to unity (horizontal solid line). This observation does not change with binding affinity threshold in (A) *A. thaliana*, (B) *M. musculus*, and (C) *N. crassa*. These data represent a sensitivity analysis of the results presented in Figs. S24A, S25A, and S26A.

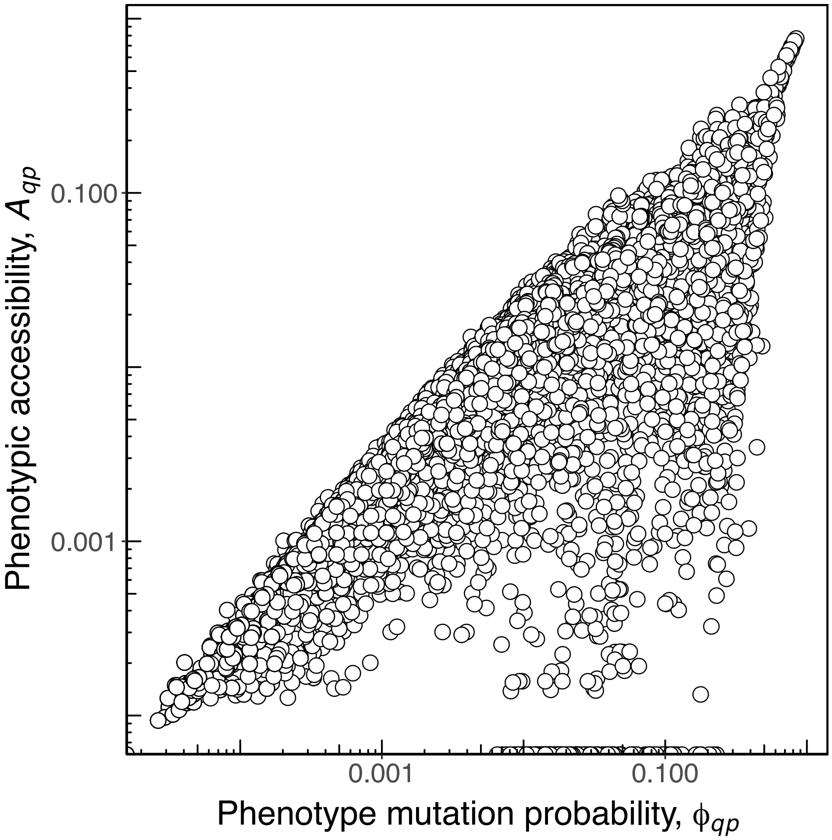

Figure S28. Phenotypic accessibility A_{qp} is strongly correlated with ϕ_{qp} (Spearman's $r = 0.95$, $p < 10^{-6}$). Each circle represents one of the 35,910 pairs of TFs from *M. musculus*. Half circles at the bottom of the panel denote pairs of phenotypes with phenotypic accessibility = 0. Note the logarithmic scale on both axes.

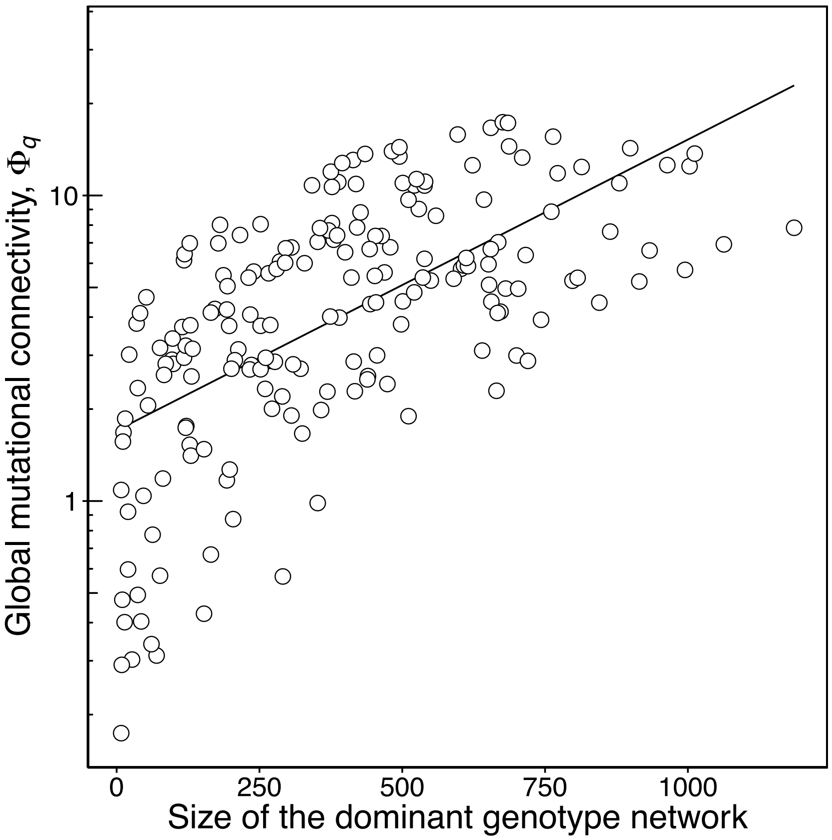

Figure S29. In *M. musculus*, the global mutational connectivity of a phenotype increases with the size of its dominant genotype network. Each circle shows the global mutational connectivity Φ_q of one of the 190 *M. musculus* TFs, as a function of the number of binding sites in its dominant genotype network. The solid line is the best linear fit to the data and is provided as a visual guide.

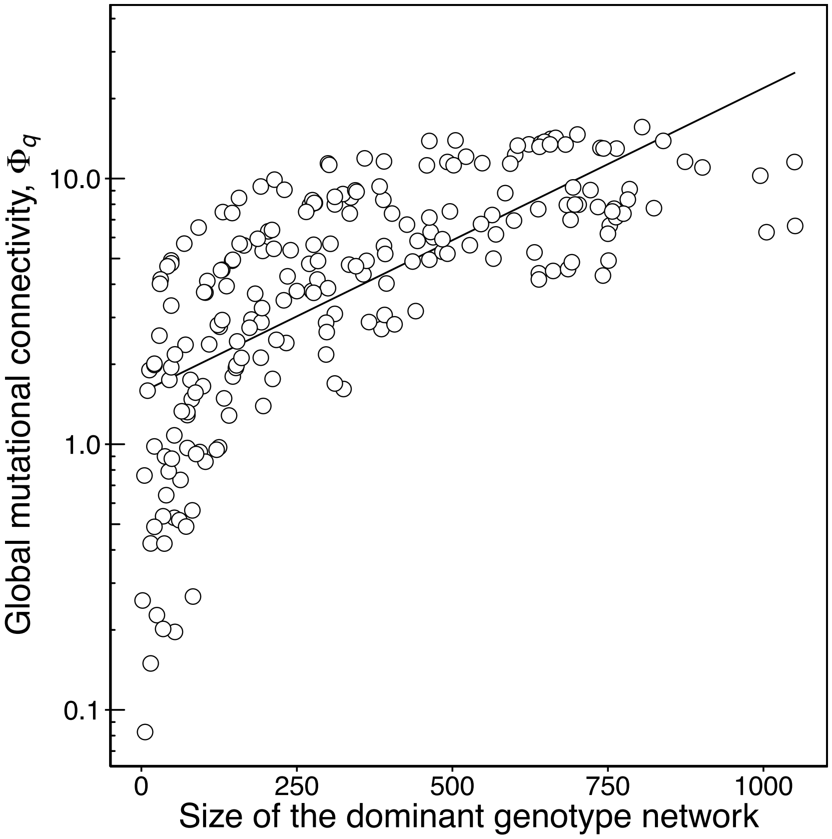

Figure S30. In *A. thaliana*, the global mutational connectivity of a phenotype increases with the size of its dominant genotype network. Each circle shows the global mutational connectivity Φ_q of one of the 217 *A. thaliana* TFs, as a function of the number of binding sites in its dominant genotype network. The solid line is the best linear fit to the data and is provided as a visual guide.

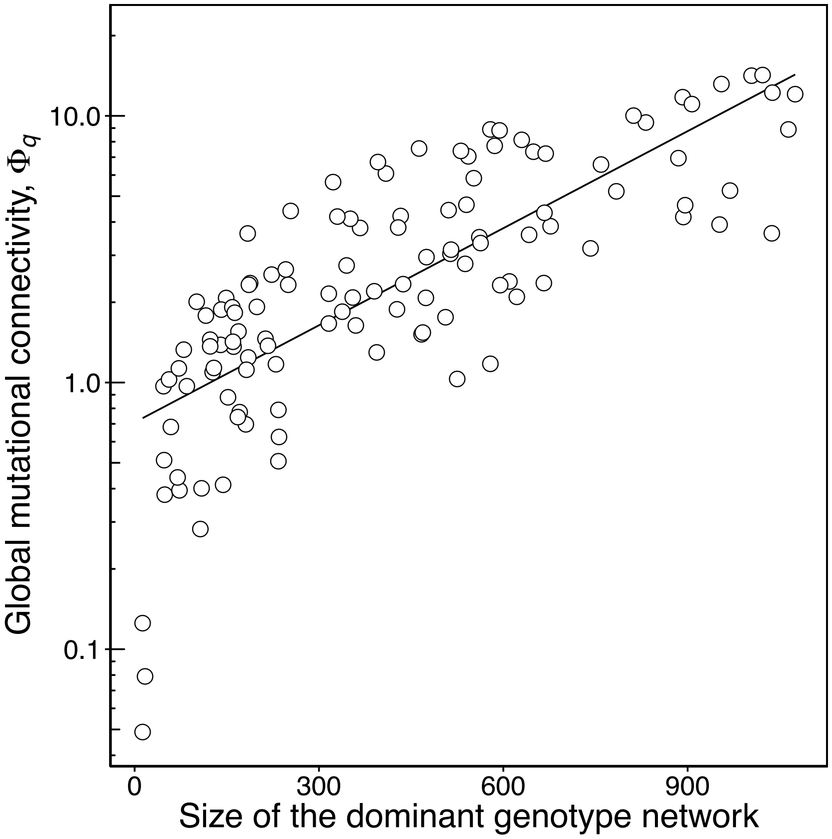

Figure S31. In *N. crassa*, the global mutational connectivity of a phenotype increases with the size of its dominant genotype network. Each circle shows the global mutational connectivity Φ_q of one of the 118 *N. crassa* TFs, as a function of the number of binding sites in its dominant genotype network. The solid line is the best linear fit to the data and is provided as a visual aid.

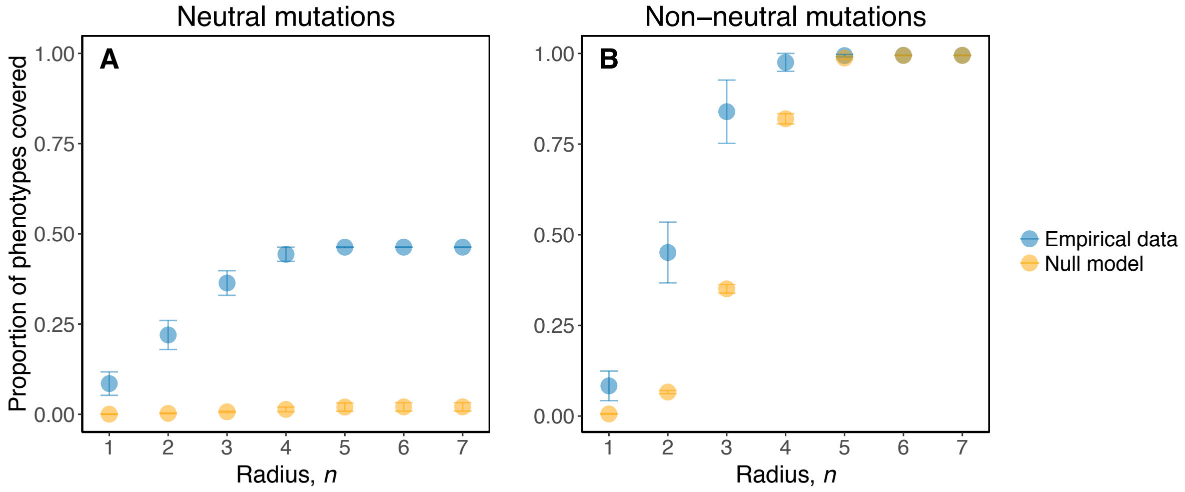

Figure S32. Comparison of phenotype space covering in the empirical data to that in the null model. The proportion of phenotypes covered as a function of the mutational radius n from a given binding site, averaged across all binding sites of the murine TF Sp110. The comparison between empirical data and the null model is made for both (A) neutral mutations and (B) non-neutral mutations. Error bars are the standard deviations of the mean. The empirical data is the same as presented in Fig. 4A.

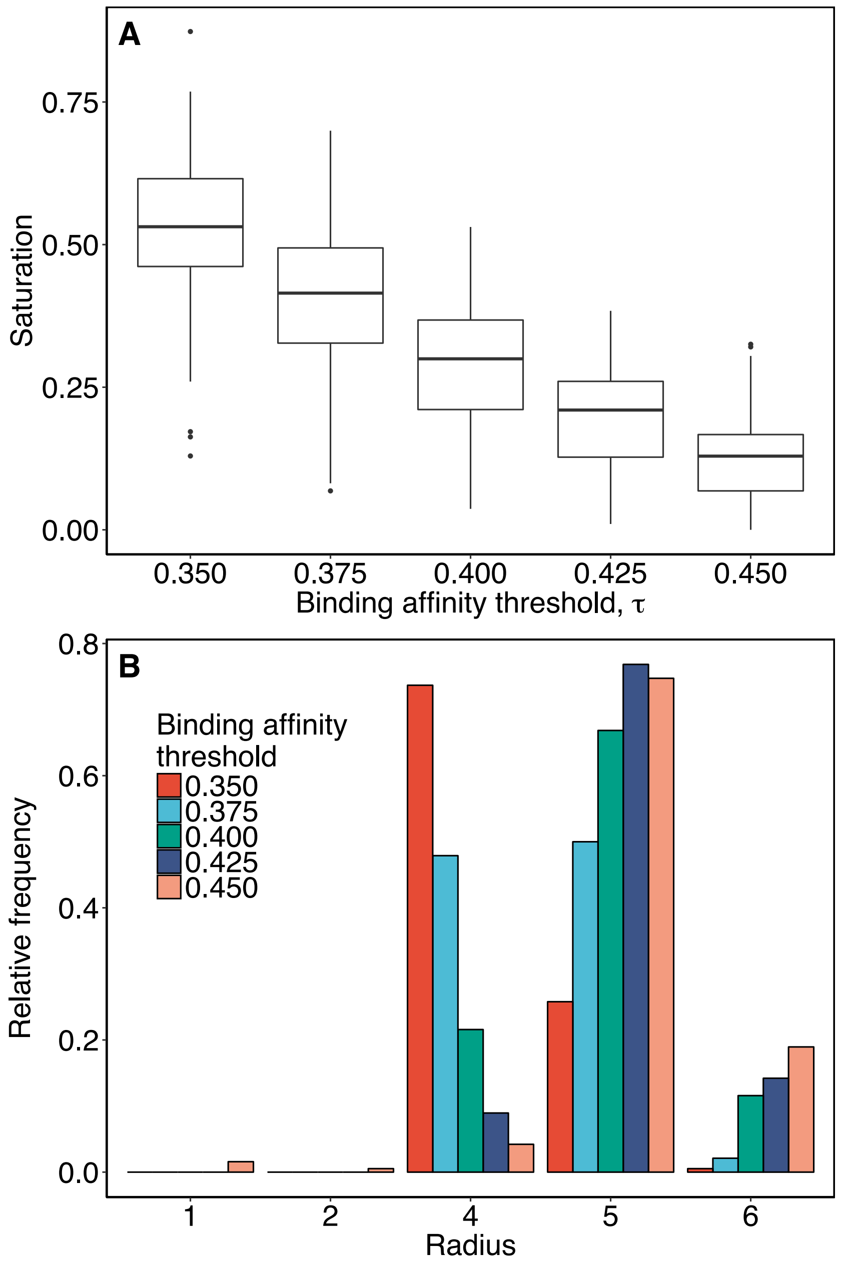

Figure S33. Phenotype space covering with different binding affinity thresholds for 190 TFs from *M. musculus.* (A) The maximum proportion of phenotypes covered by neutral mutations as a function of the binding affinity threshold, for all 190 murine TFs. (B) The distribution of the average mutational radius n that covers all phenotypes in genotype space for different binding affinity thresholds.

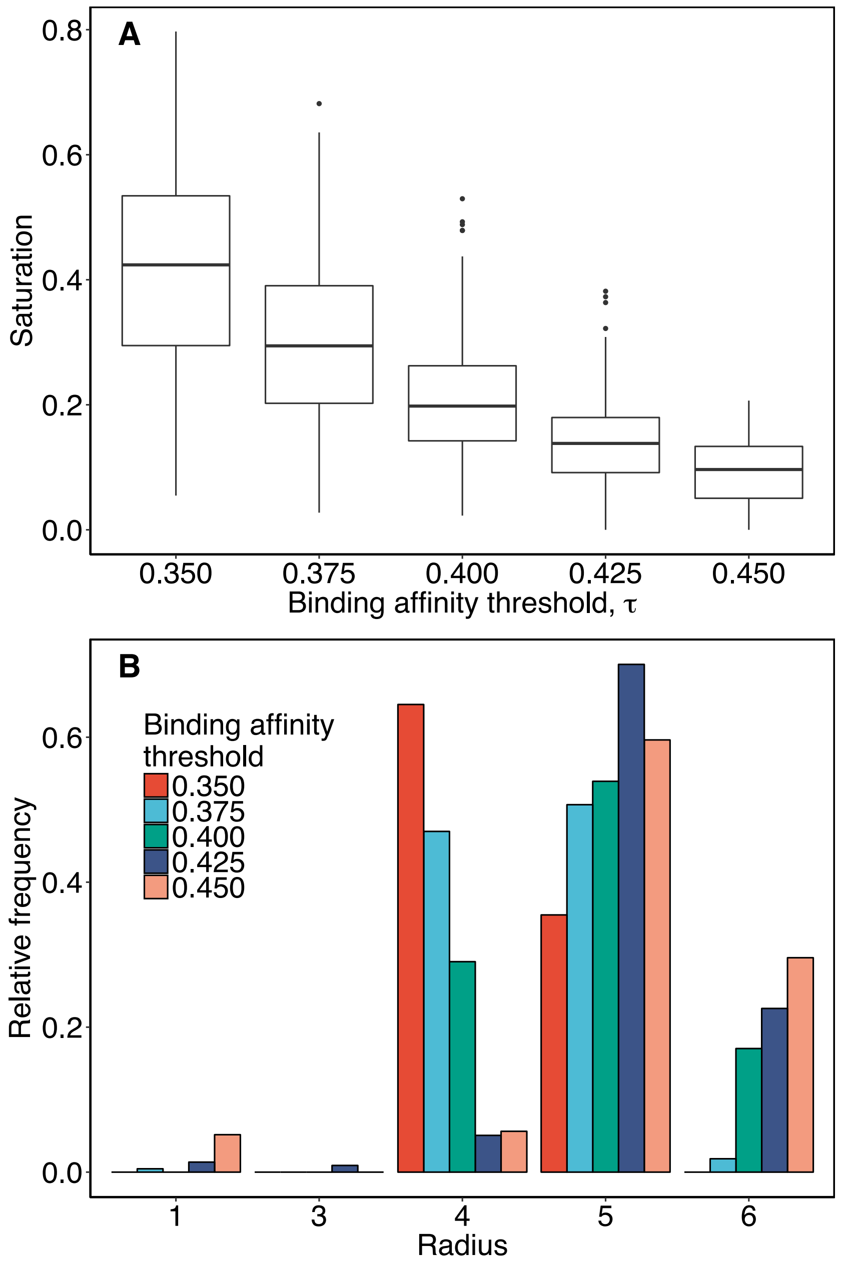

Figure S34. Phenotype space covering with different binding affinity thresholds for 217 TFs from *A. thaliana.* (A) The maximum proportion of phenotypes covered by neutral mutations as a function of the binding affinity threshold, for all 217 plant TFs. (B) The distribution of the average mutational radius n that covers all phenotypes in genotype space for different binding affinity thresholds.

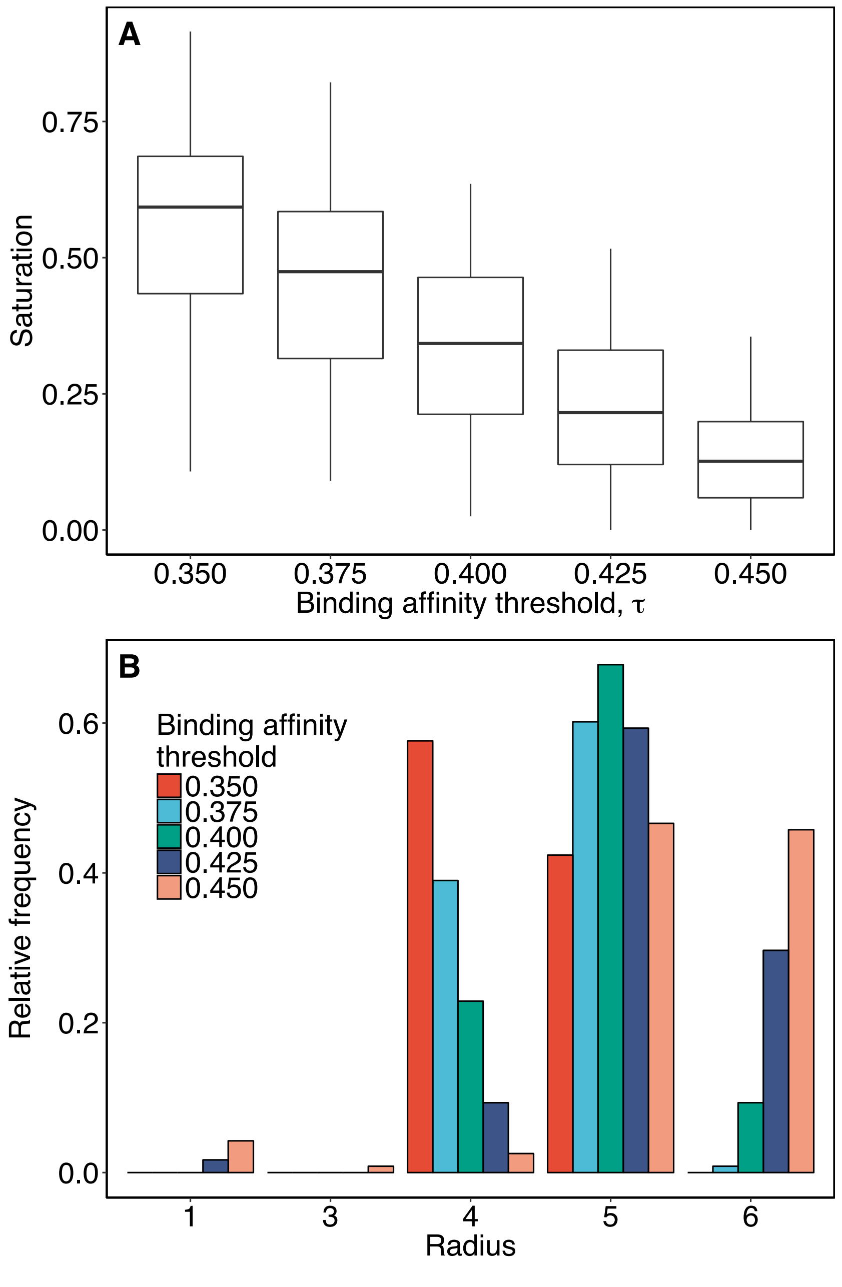

Figure S35. Phenotype space covering with different binding affinity thresholds for 118 TFs from *N. crassa*. (A) The maximum proportion of phenotypes covered by neutral mutations as a function of the binding affinity threshold, for all 118 fungal TFs. (B) The distribution of the average mutational radius n that covers all phenotypes in genotype space for different binding affinity thresholds.

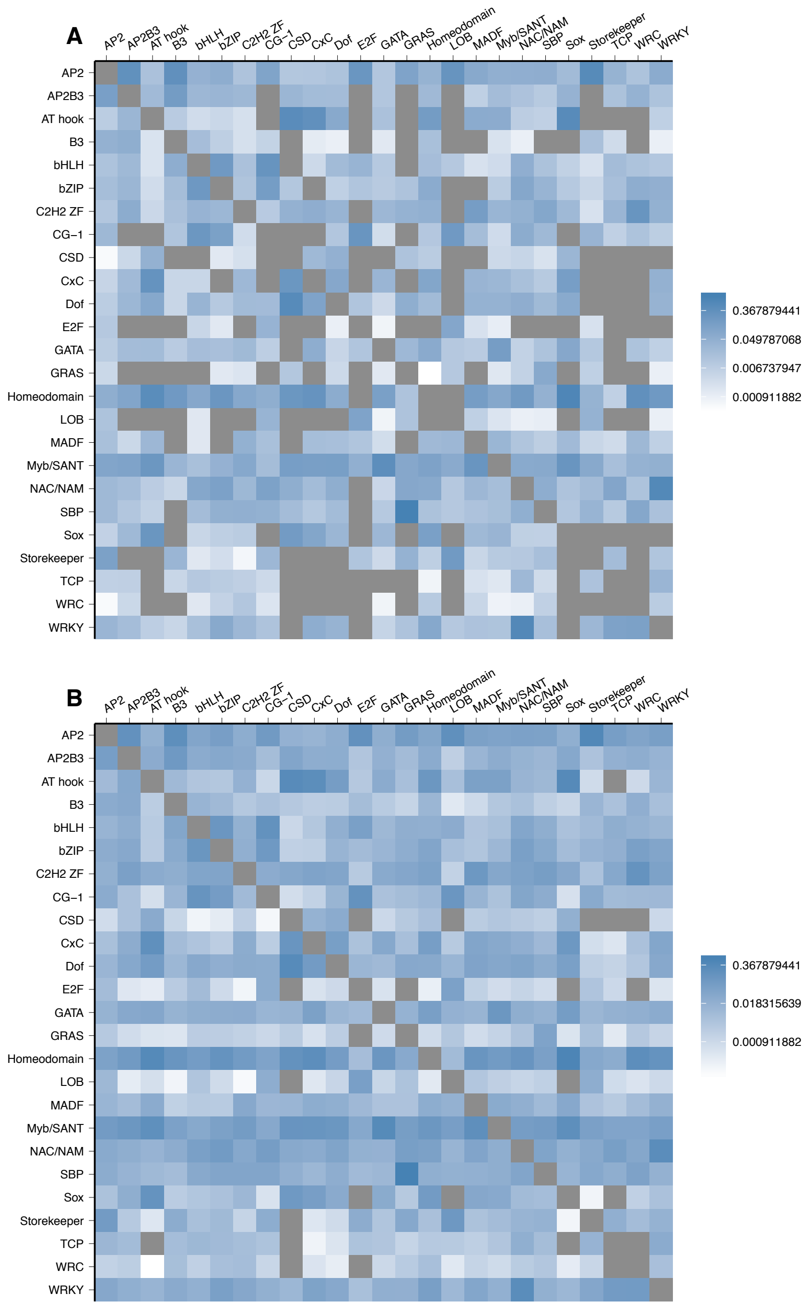

Figure S36. Matrices of internetwork relationships for the genotype networks of DNA-binding domains from *A. thaliana*. Heatmaps of log10-transformed (A) overlap and (B) ϕ_{qp}, the probability of mutating from the genotype network of phenotype p to the genotype network of phenotype q. Each row and column represents a different DNA-binding domain genotype network. Domains are ordered alphabetically. Cells colored in gray indicate either N/A values (on the diagonal) or values equal to zero (off-diagonal).

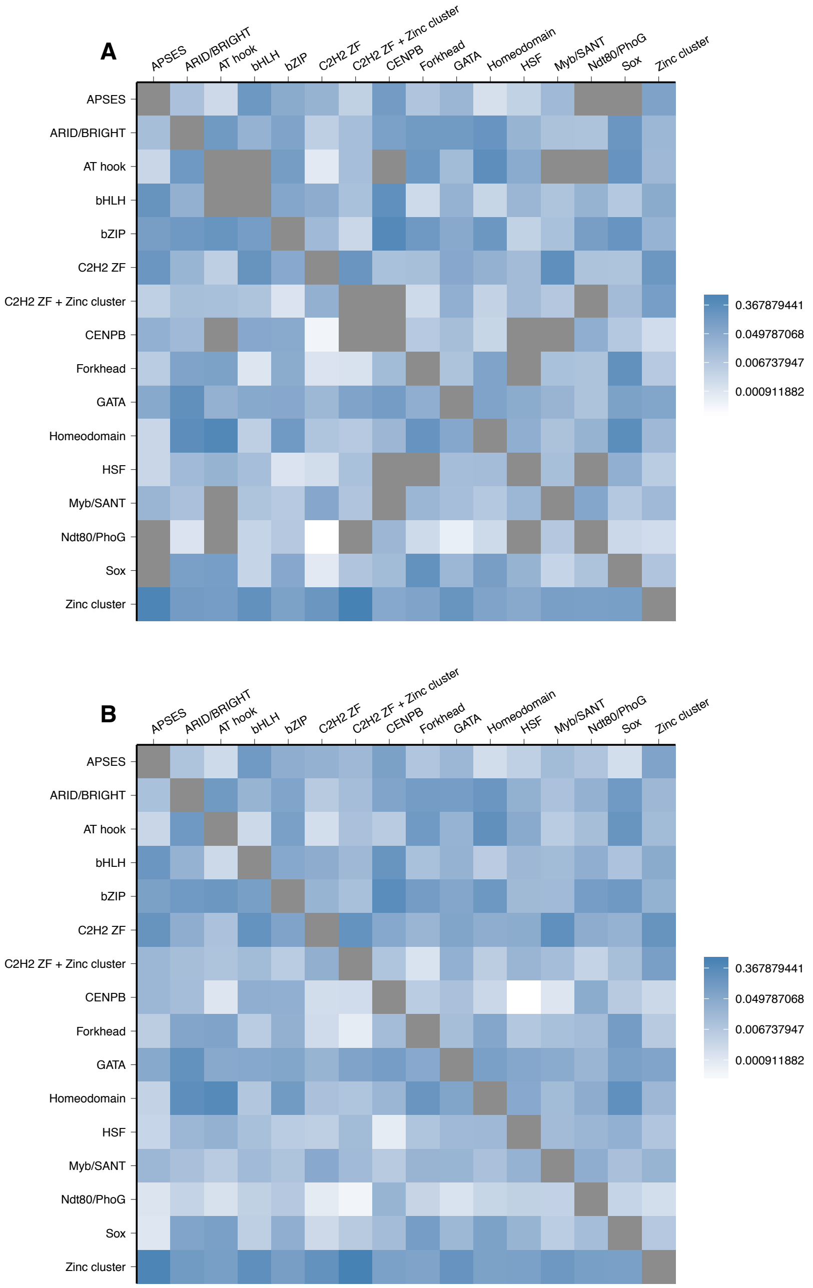

Figure S37. Matrices of internetwork relationships for the genotype networks of DNA-binding domains from *N. crassa*. Heatmaps of log10-transformed (A) overlap and (B) ϕ_{qp}, the probability of mutating from the genotype network of phenotype p to the genotype network of phenotype q. Each row and column represents a different DNA-binding domain genotype network. Domains are ordered alphabetically. Cells colored in gray indicate either N/A values (on the diagonal) or values equal to zero (off-diagonal).

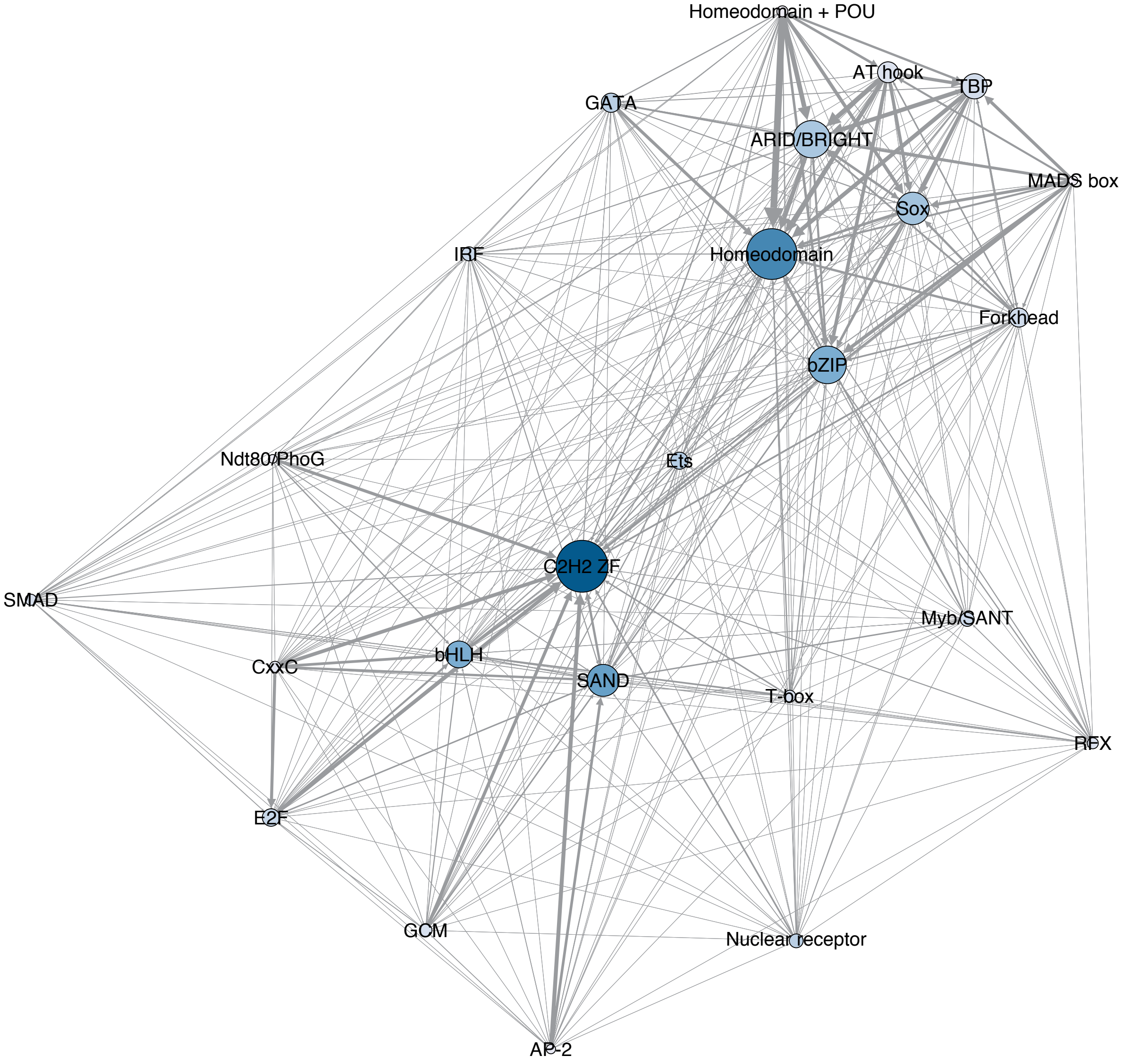

Figure S38. Phenotype network for 25 DNA-binding domains from *M. musculus*. The nodes in this network represent the dominant genotype networks of DNA binding domains, and edges connect nodes if their corresponding genotype networks are connected by at least one non-neutral mutation. The size of the edges is proportional to the ϕ_{qp} among domains. Node size is proportional to the size of the associated genotype network. Node color represents the global mutational connectivity Φ_q of each domain (darker nodes have larger Φ_q).

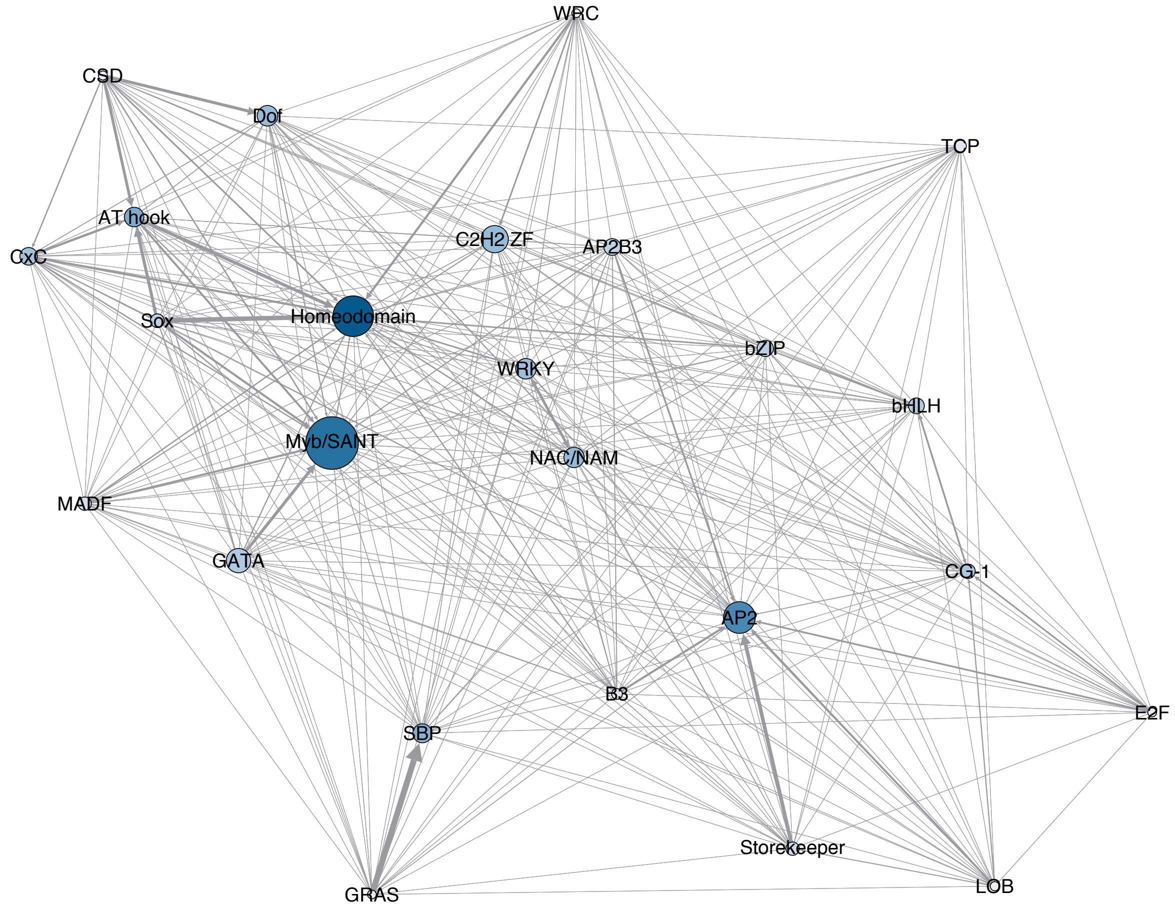

Figure S39. Phenotype network for 25 DNA-binding domains from *A. thaliana*. The nodes in this network represent the dominant genotype networks of DNA binding domains, and edges connect nodes if their corresponding genotype networks are connected by at least one non-neutral mutation. The size of the edges is proportional to the ϕ_{qp} among domains. Node size is proportional to the size of the associated genotype network. Node color represents the global mutational connectivity Φ_q of each domain (darker nodes have larger Φ_q).

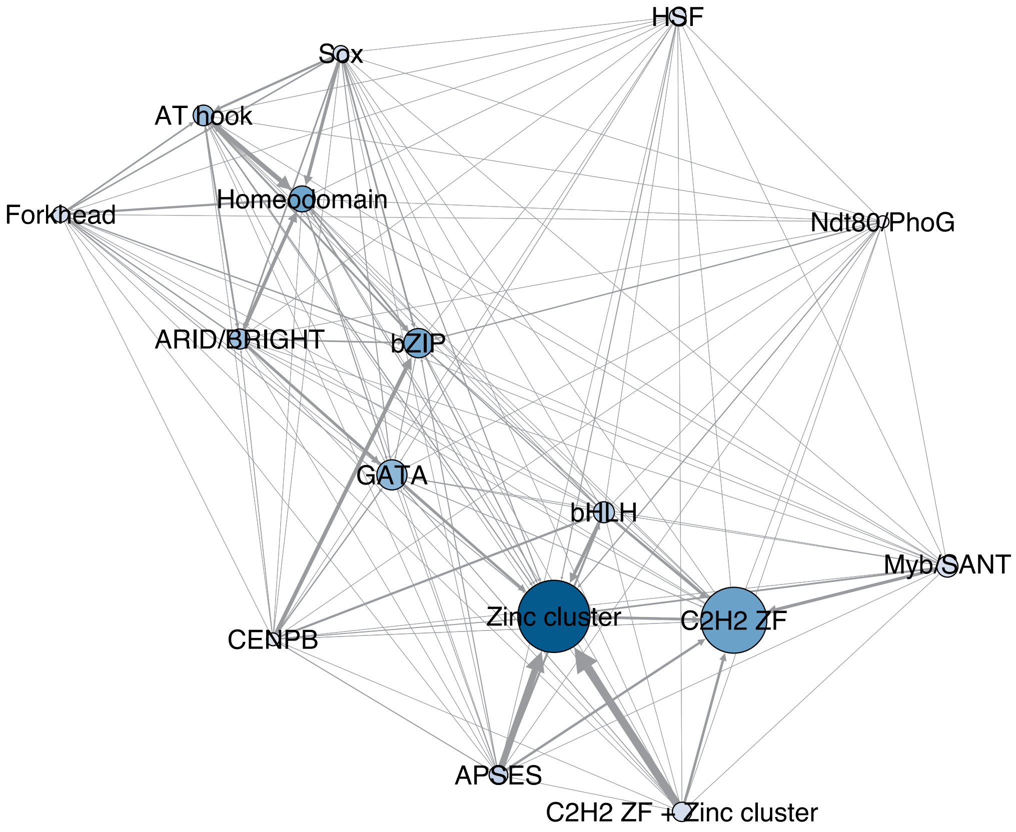

Figure S40. Phenotype network for 16 DNA-binding domains from *N. crassa*. The nodes in this network represent the dominant genotype networks of DNA binding domains, and edges connect nodes if their corresponding genotype networks are connected by at least one non-neutral mutation. The size of the edges is proportional to the ϕ_{qp} among domains. Node size is proportional to the size of the associated genotype network. Node color represents the global mutational connectivity Φ_q of each domain (darker nodes have larger Φ_q).

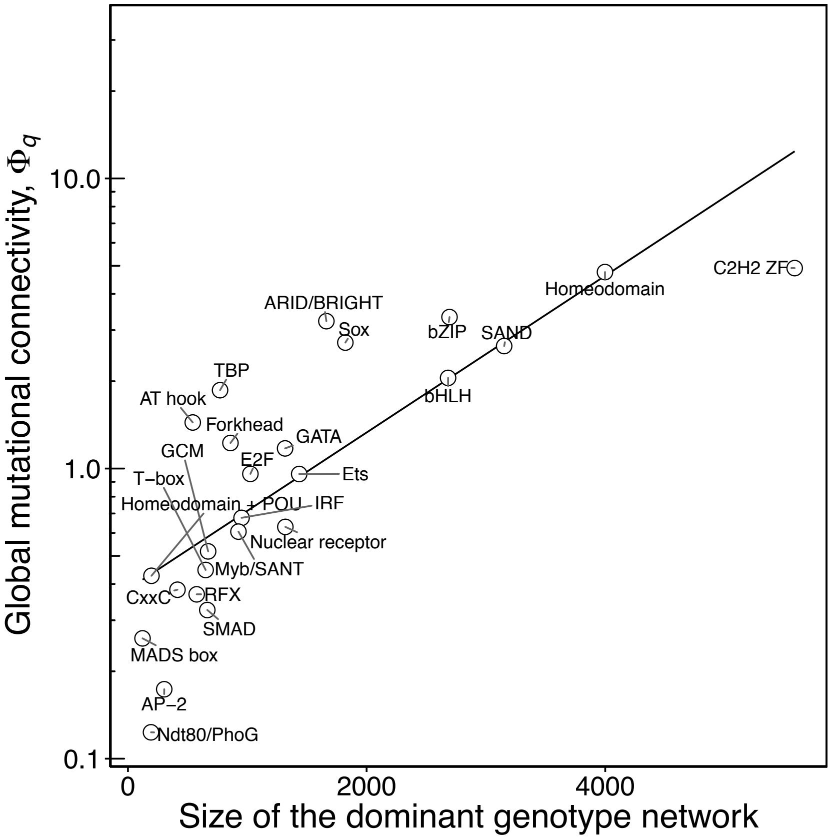

Figure S41. In *M. musculus*, the global mutational connectivity of a phenotype increases with the size of its dominant genotype network. Each circle shows the global mutational connectivity Φ_q of one of the 25 *M. musculus* DNA binding domains, as a function of the number of binding sites in its dominant genotype network. The solid line is the best linear fit to the data and is provided as a visual aid.

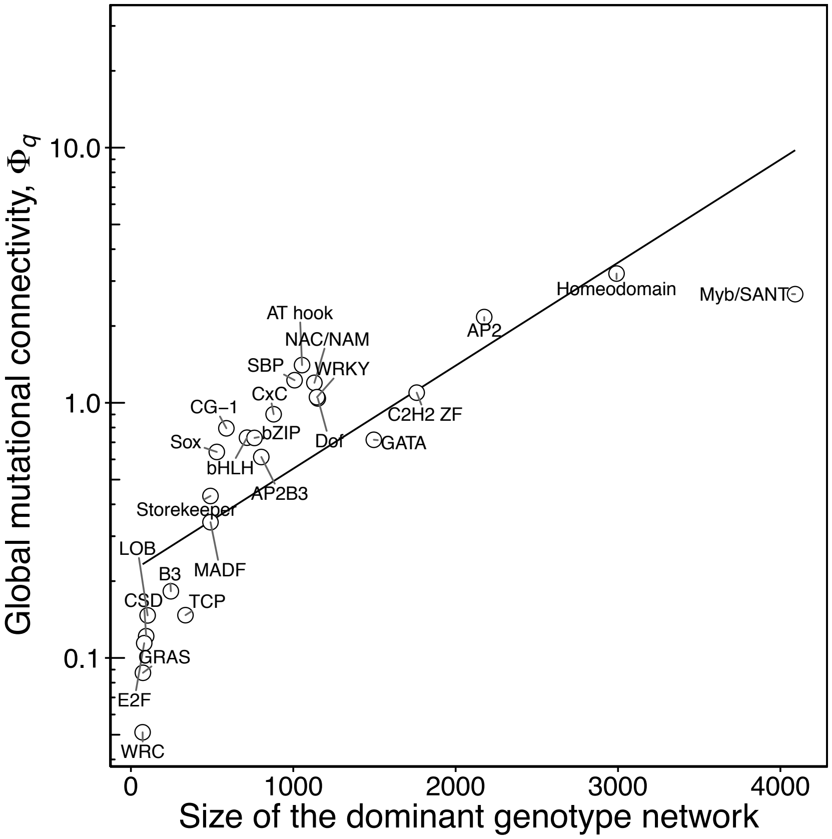

Figure S42. In *A. thaliana*, the global mutational connectivity of a phenotype increases with the size of its dominant genotype network. Each circle shows the global mutational connectivity Φ_q of one of the 25 *A. thaliana* DNA binding domains, as a function of the number of binding sites in its dominant genotype network. The solid line is the best linear fit to the data and is provided as a visual aid.

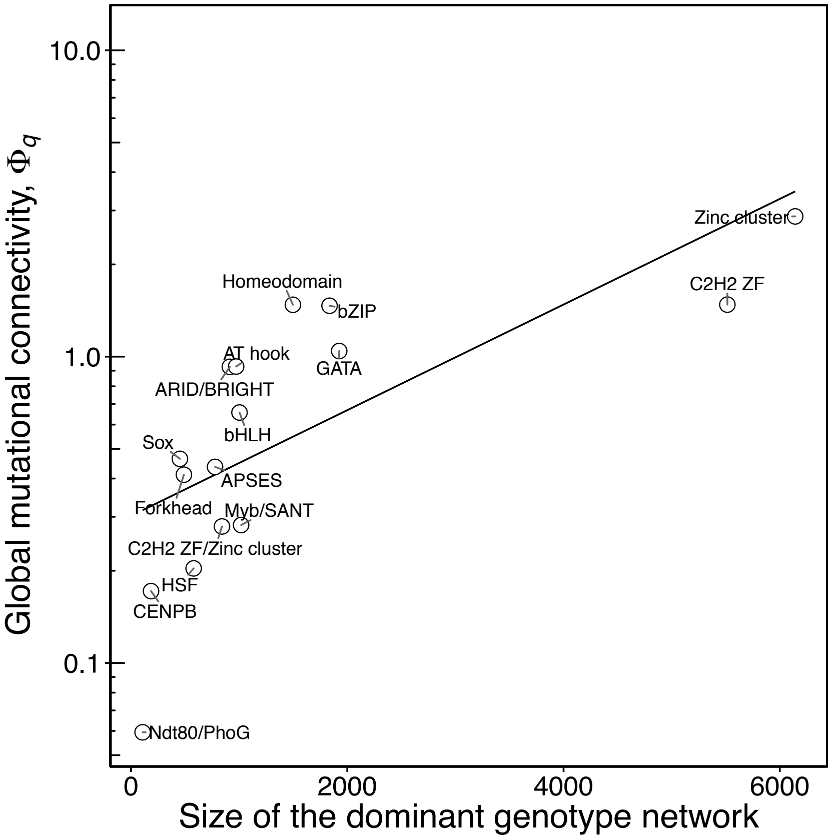

Figure S43. In *N. crassa*, the global mutational connectivity of a phenotype increases with the size of its dominant genotype network. Each circle shows the global mutational connectivity Φ_q of one of the 16 *N. crassa* DNA binding domains, as a function of the number of binding sites in its dominant genotype network. The solid line is the best linear fit to the data and is provided as a visual aid.

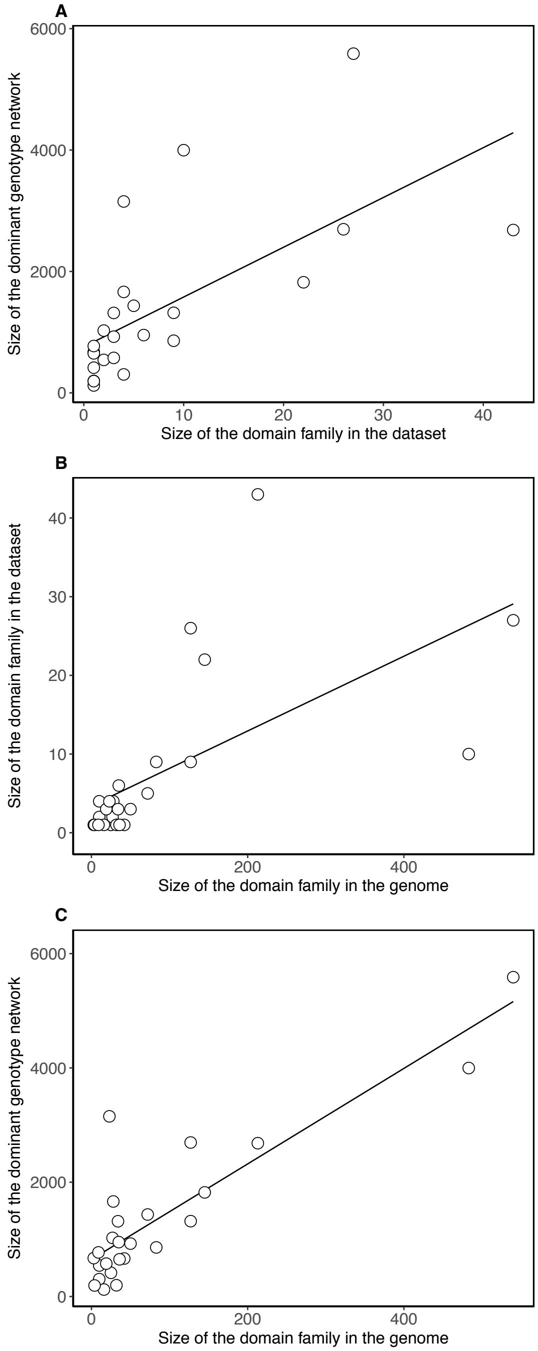

Figure S44. Binding domains with more TFs have larger genotype networks in *M. musculus*. (A) The relationship between the size of a binding domain's dominant genotype network and the number of TFs per domain in our dataset (Spearman's $r = 0.8$, $p = 2 \times 10^{-6}$). (B) The relationship between the number of TFs per binding domain in our dataset and the number of TFs per binding domain in the *M. musculus* genome (Spearman's $r = 0.75$, $p = 1.4 \times 10^{-5}$). (C) The relationship between the size of a binding domain's dominant genotype network and the number of TFs per binding domain in the *M. musculus* genome (Spearman's $r = 0.7$, $p = 9.6 \times 10^{-5}$). In each panel, each circle represents one of the 25 *M. musculus* binding domains in our dataset.

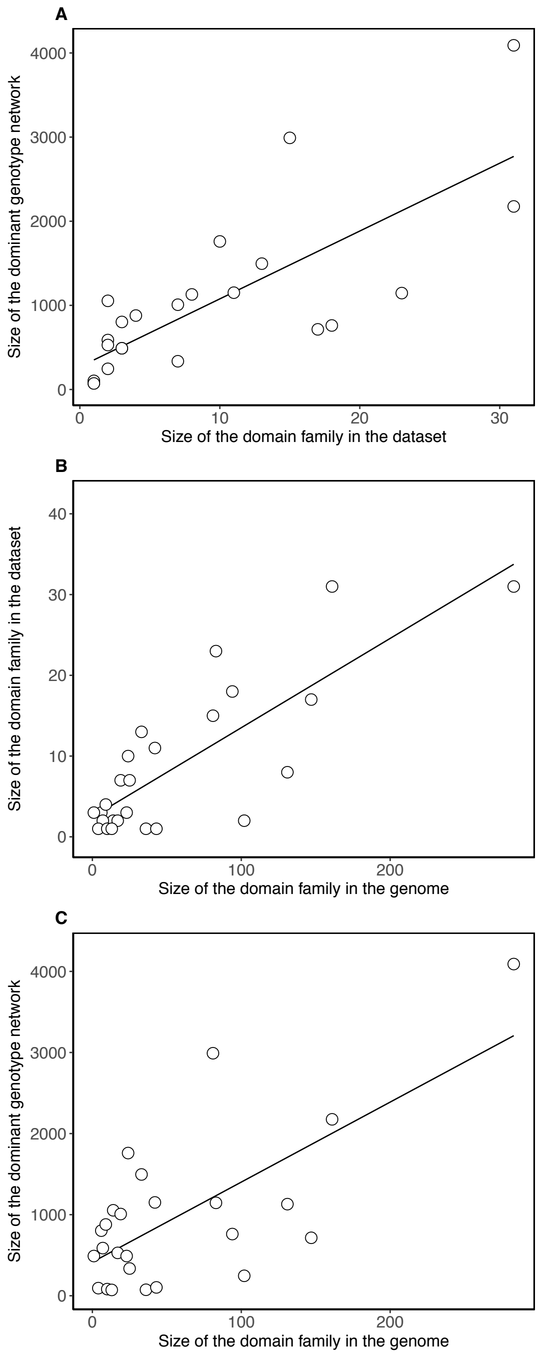

Figure S45. Binding domains with more TFs have larger genotype networks in *A. thaliana*. (A) The relationship between the size of a binding domain's dominant genotype network and the number of TFs per domain in our dataset (Spearman's $r = 0.83$, $p = 2.8 \times 10^{-7}$). (B) The relationship between the number of TFs per binding domain in our dataset and the number of TFs per binding domain in the *A. thaliana* genome (Spearman's $r = 0.64$, $p = 5.8 \times 10^{-4}$). (C) The relationship between the size of a binding domain's dominant genotype network and the number of TFs per binding domain in the *A. thaliana* genome (Spearman's $r = 0.44$, $p = 2.9 \times 10^{-2}$). In each panel, each circle represents one of the 25 *A. thaliana* binding domains in our dataset.

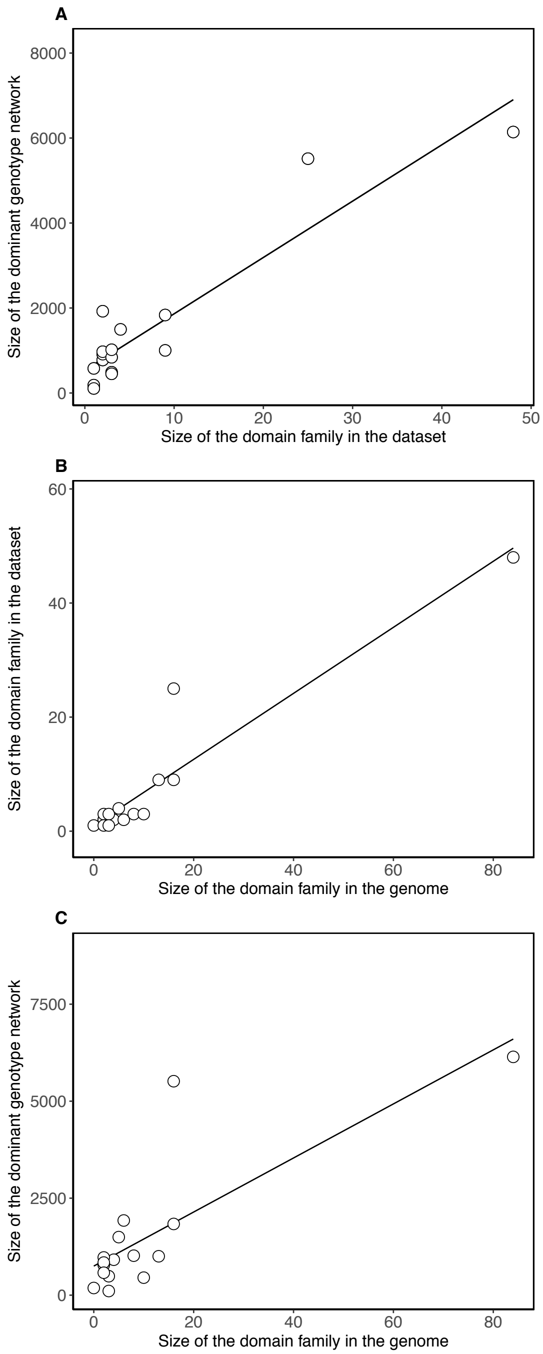

Figure S46. Binding domains with more TFs have larger genotype networks in *N. crassa*. (A) The relationship between the size of a binding domain's dominant genotype network and the number of TFs per domain in our dataset (Spearman's $r = 0.93$, $p = 2 \times 10^{-7}$). (B) The relationship between the number of TFs per binding domain in our dataset and the number of TFs per binding domain in the *N. crassa* genome (Spearman's $r = 0.94$, $p = 4.8 \times 10^{-8}$). (C) The relationship between the size of a binding domain's dominant genotype network and the number of TFs per binding domain in the *N. crassa* genome (Spearman's $r = 0.8$, $p = 2.3 \times 10^{-4}$). In each panel, each circle represents one of the 16 *N. crassa* binding domains in our dataset.
